# Nondestructive cellular-level 3D observation of mouse kidney using laboratory-based X-ray microscopy with paraffin-mediated contrast enhancement

**DOI:** 10.1038/s41598-022-13394-9

**Published:** 2022-06-08

**Authors:** Naoki Kunishima, Raita Hirose, Yoshihiro Takeda, Koichiro Ito, Kengo Furuichi, Kazuhiko Omote

**Affiliations:** 1 X-Ray Research Laboratory Rigaku Corporation, 3-9-12 Matsubara-cho, Akishima, Tokyo 196-8666 Japan; 2 New Market Development Office, Rigaku Corporation, 3-9-12 Matsubara-cho, Akishima, Tokyo 196-8666 Japan; 3 Department of Nephrology, School of Medicine, Kanazawa Medical University, 1-1 Daigaku, Uchinada, Kahoku, Ishikawa 920-0293 Japan

**Keywords:** Nephrology, Imaging

## Abstract

For three-dimensional observation of unstained bio-specimens using X-ray microscopy with computed tomography (CT), one main problem has been low contrast in X-ray absorption. Here we introduce paraffin-mediated contrast enhancement to visualize biopsy samples of mouse kidney using a laboratory-based X-tray microscope. Unlike conventional heavy-atom staining, paraffin-mediated contrast enhancement uses solid paraffin as a negative contrast medium to replace water in the sample. The medium replacement from water to paraffin effectively lowers the absorption of low-energy X-rays by the medium, which eventually enhances the absorption contrast between the medium and tissue. In this work, paraffin-mediated contrast enhancement with 8 keV laboratory X-rays was used to visualize cylindrical renal biopsies with diameters of about 0.5 mm. As a result, reconstructed CT images from 19.4 h of data collection achieved cellular-level resolutions in all directions, which provided 3D structures of renal corpuscles from a normal mouse and from a disease model mouse. These two structures with and without disease allowed a volumetric analysis showing substantial volume differences in glomerular subregions. Notably, this nondestructive method presents CT opacities reflecting elemental composition and density of unstained tissues, thereby allowing more unbiased interpretation on their biological structures.

## Introduction

Medical care for nephropathy is a central issue in medicine since the global prevalence of chronic kidney disease is estimated to be 9.1%^1^. For the clinical diagnosis of human kidney diseases, histological cellular- to subcellular-level 2D observation of renal biopsy samples using optical (visible-light) microscopy and electron microscopy is performed routinely^2^, which requires a destructive/laborious sectioning of samples. The biopsy is a cylindrical piece of tissue with a diameter of 0.5–1.0 mm and a length of a few millimeters. In the current procedure for the observation of a biopsy sample, only a limited number of sections for diagnosis are selected without reliable prior knowledge and remaining major parts are discarded without evaluation. As a result, significant information in the renal biopsy is lost, which is a big problem in the conventional 2D method. Laboratory-based X-ray microscopy adopting computed tomography (CT) enables non-destructive 3D visualization at cellular-level resolutions^3,4^. With the goal of developing more efficient diagnoses of human renal biopsies, we used a compact commercial laboratory-based X-ray microscope, nano3DX, (Rigaku Corporation, Tokyo, Japan) to observe unstained/paraffin-embedded mouse renal biopsies.

In the observation of bio-specimens using X-ray microscopy, one problem has been low contrast in X-ray absorption. For instance, although laboratory-based X-ray microscopy with Mo-target X-rays (17.5 keV) allowed observation of unstained/paraffin-embedded human lung biopsies, its spatial resolution was limited as tissue level due to low absorption contrast^5,6^. To enhance the X-ray absorption contrast, heavy-atom staining was the first choice to date. However, unstained bio-specimens are preferable to preserve their native structures. Phase contrast methods are known to be effective in improving this contradicting problem. For instance, propagation-based phase retrieval^7–9^ was used to visualize paraffin-embedded unstained brain cells^10^, and Zernike phase-contrast optics^3^ were used to visualize paraffin-embedded unstained organ tissues^11^. However, these cellular- to subcellular-level observations required synchrotron X-rays which are not always accessible to general users. Although speckle-based X-ray phase tomography at tissue-level resolution was reported to visualize a whole, unstained, hydrated mouse kidney^12^, that technique also required synchrotron experiments. For that reason, attempts were made to combine phase contrast methods with laboratory-based X-ray microscopy^3,13,14^ and use them to observe various bio-specimens at cellular-level resolutions^4,15–19^. However, propagation-based phase retrieval is not necessarily applicable to all samples, and Zernike phase contrast has a limited field-of-view of less than 100 μm. Another phase contrast method using a grating interferometer combined with laboratory X-ray sources^20^ has limited spatial resolutions in the range of 40–50 μm. Therefore, because the applicability of phase contrast methods at cellular-level resolutions is still limited, another method with better versatility is desirable.

Here we show that paraffin-mediated enhancement of absorption contrast in X-ray microscopy with 8 keV rotating-anode X-rays can achieve cellular-level resolutions for a biopsy-sized unstained bio-specimen, without using heavy-atom staining or phase contrast methods. In paraffin-mediated contrast enhancement, solid paraffin is used as a negative contrast medium which replace water in the sample. The medium replacement from water to paraffin effectively lowers absorption by the medium so as to enhance the absorption contrast between the medium and tissue. Although previous reports^5,6,10,11,15,17,18^ commonly used paraffin-embedded samples, they did not consider the paraffin as the negative contrast medium. Although the concept of negative contrast enhancement is known in gastroenterology for use in diagnostic X-ray CT where a low-density liquid material is used to visualize intestinal tracts^21^, it was not explicitly considered in X-ray microscopy to date.

The functional unit of the kidney, a vertebrate’s fundamental urine-producing organ, is called the “nephron”^22^ comprising a renal corpuscle and a renal tubule. The renal corpuscle is composed of a tuft of capillary arterioles called the “glomerulus” and surrounding origin of the renal tubule called “Bowman’s capsule.” The 3D structure of a nephron can be reconstructed by serial sectioning based on many consecutive 2D images by electron microscopy^23–25^ or optical microscopy^26–28^. Although serial sectioning contributed significantly to kidney research, this method is destructive and shows anisotropy in spatial resolution due to limited information along the direction of the sample thickness. To solve these problems, X-ray microscopy was adopted to observe an osmium-stained kidney sample using synchrotron radiation^29^, which successfully demonstrated the applicability of X-ray microscopy for visualization of single nephrons. However, heavy-atom staining is difficult to control because it depends on many factors, such as reagent type and condition, reaction time, sample size, and so on (Supplementary Fig. S1). Furthermore, it tends to emphasize materials that have high affinity for the heavy-atom reagent, which may mislead interpretation. The method presented here using paraffin-mediated contrast enhancement may resolve these problems. In this work, we focused on the observation of the glomerulus as a major target of clinical diagnosis, and found that lower energy X-rays had better contrast in paraffin-embedded tissues, suggesting its potential in 3D histopathology imaging.

## Results

### Paraffin-mediated contrast enhancement

**Figure 1:**
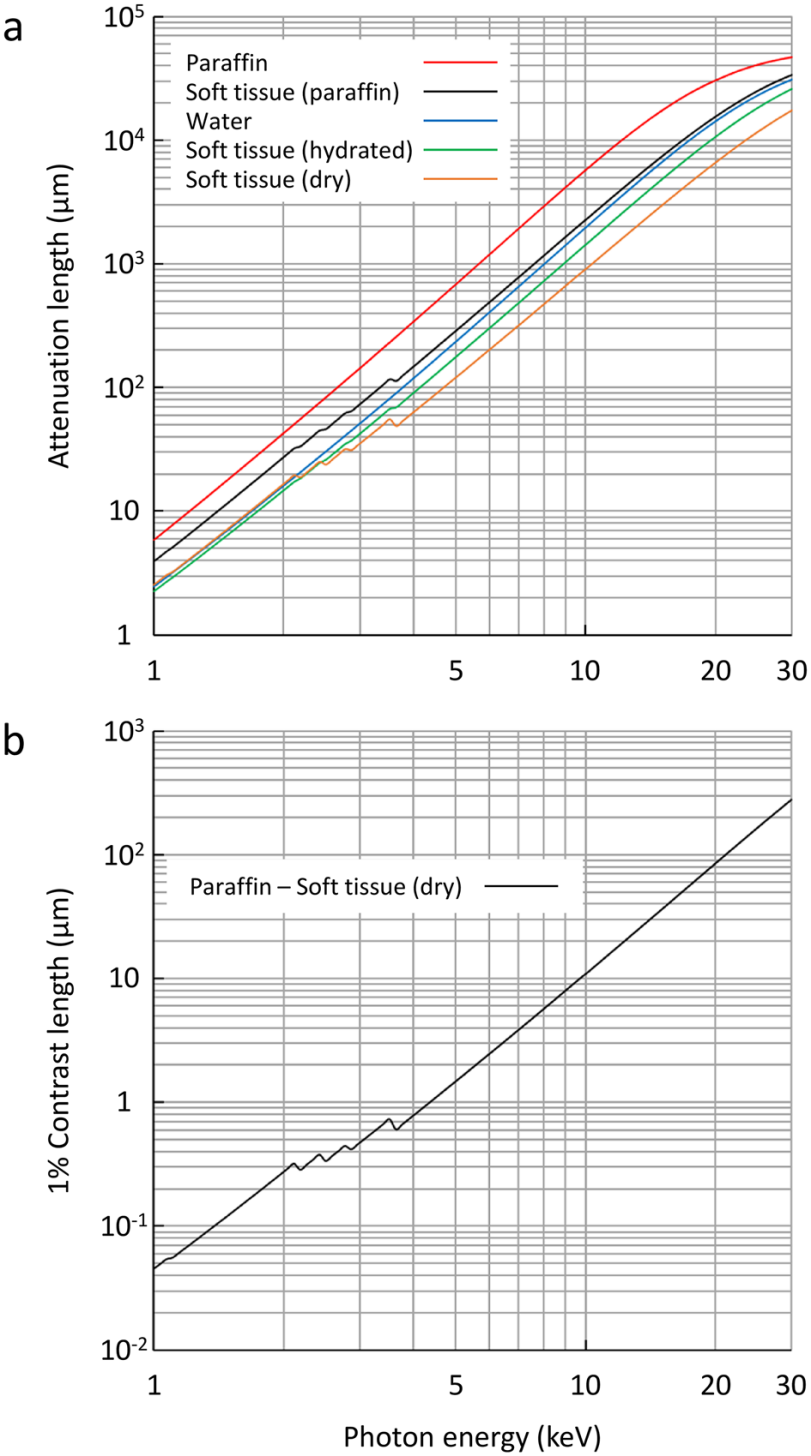
Curves of X-ray absorption versus photon energy. (**a**) X-ray attenuation length in μm. Color codes used were: red for paraffin (A_p_), black for paraffin-embedded soft tissue (A_pt_), blue for water (A_w_), green for hydrated soft tissue (A_ht_), and orange for dehydrated (dry) soft tissue (A_dt_). Gain in the medium:sample ratio of attenuation length upon the medium replacement from water to paraffin can be calculated as (A_p_/A_dt_)/(A_w_/A_dt_) = A_p_/A_w_ when A_w_ ≥ A_dt_, or (A_p_/A_dt_)/(A_dt_/A_w_) when A_w_ < A_dt_. Water content by weight, volumetric mass density, and elemental composition for the hydrated soft-tissue (without adipose tissue) were taken from literature^37^ as 70%, 1.06, and H_10.2_C_14.3_N_3.4_O_70.8_Na_0.2_P_0.3_S_0.3_Cl_0.2_K_0.3_, respectively, which were used to calculate density and elemental composition for the dehydrated soft-tissue as 1.23 and H_8.1_C_47.6_N_11.3_O_28.6_Na_0.7_P_1_S_1_Cl_0.7_K_1_, respectively. Similarly, density and elemental composition for the paraffin-embedded soft-tissue were calculated as 0.99 (0.986) and H_5.3_C_80.4_N_3.7_O_9.3_Na_0.2_P_0.3_S_0.3_Cl_0.2_K_0.3_, respectively. Attenuation length was calculated from density and elemental composition using a web tool (https://henke.lbl.gov/optical_constants/atten2.html)^38^; densities for paraffin (H_1_C_2_) and water (H_2_O_1_) were set as 0.90 and 1.00, respectively. (**b**) 1% Contrast length in μm. Length giving 1% difference in X-ray transmission between paraffin and dehydrated soft tissue was calculated as A_p_ × A_dt_ × ln(0.99)/(A_dt_-A_p_).

The principle of paraffin-mediated contrast enhancement in X-ray microscopy is explained as follows. In a wide energy range of 2–15 keV, the X-ray attenuation length of paraffin is about three times longer than that of water, which is the source of contrast enhancement upon medium replacement (Fig. 1a). The lower limit of X-ray energy for the observation of a paraffin-embedded soft-tissue is around 4 keV, if the sample can be trimmed to be 50–100 μm in size. On the other hand, the length giving 1% difference in X-ray transmission (1% contrast length) between paraffin and dehydrated soft tissue is calculated as 5.6 μm at 8 keV, indicating that this energy is suitable for cellular-level observation of a paraffin-embedded renal biopsy (Fig. 1b). If we assume that the cellular size of about 20 μm is unchanged upon medium replacement from water to paraffin, the upper limit of cellular-level observations is around 12 keV. Collectively, the range of X-ray energies acceptable for cellular- to subcellular-level observations of bio-specimens with paraffin-mediated contrast enhancement would be 4–12 keV.

### Observation of unstained mouse kidney

**Figure 2:**
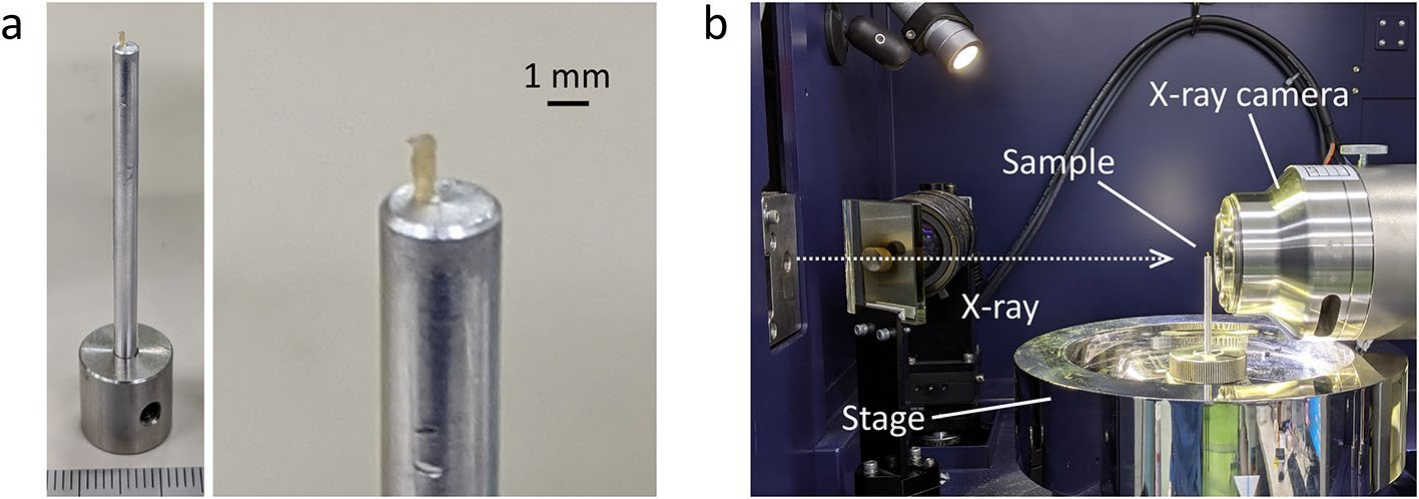
Experiment setup of laboratory-based X-ray microscopy. (**a**) Photograph of direct-mounted kidney sample. Entire image (left) and magnified part around the sample (right) are shown. (**b**) Configuration of X-ray scanning. Representative parts of the apparatus are labeled with names.

**Figure 3:**
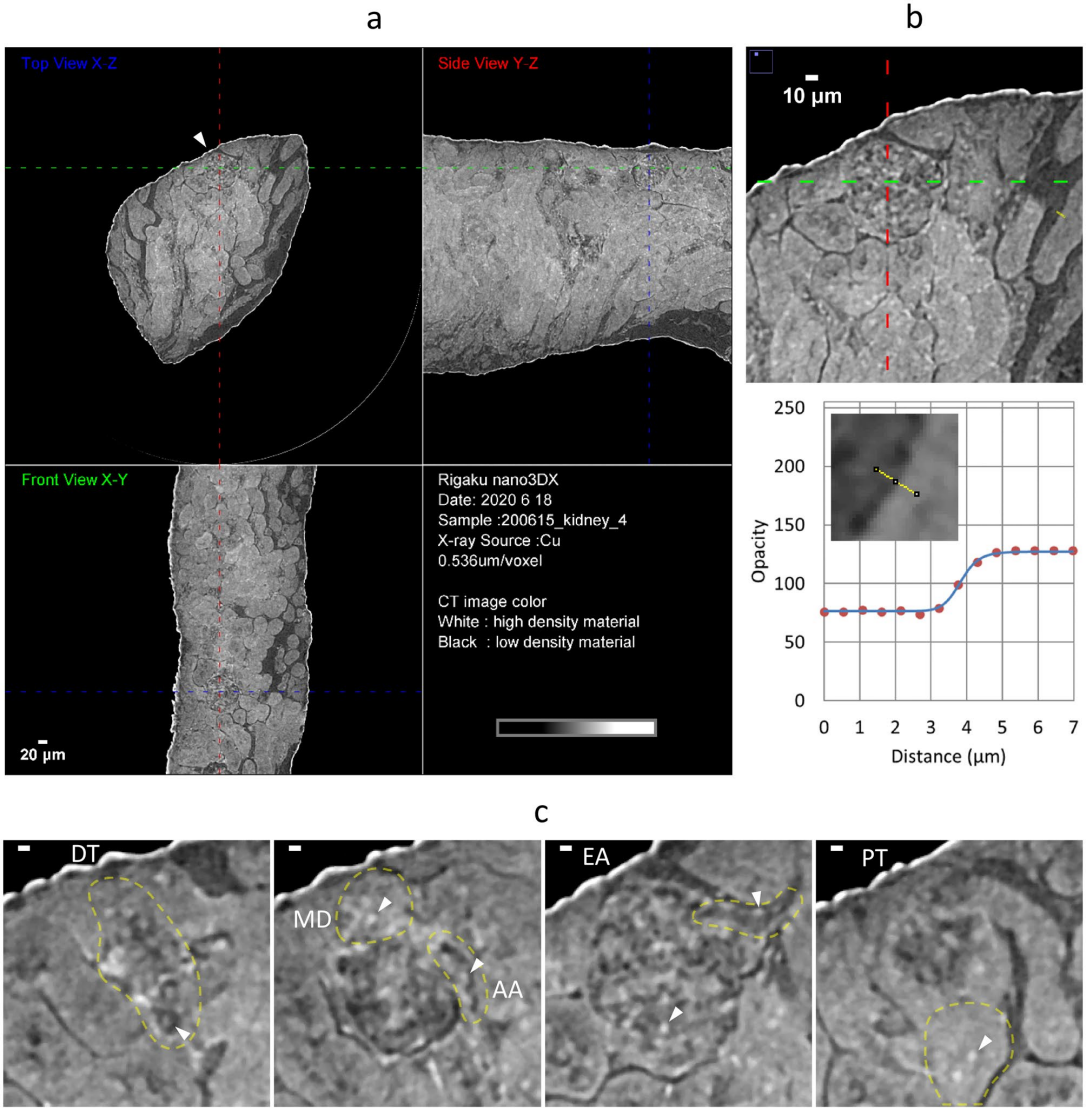
CT images at cellular-level resolution from laboratory-based X-ray microscopy with paraffin-mediated contrast enhancement. (**a**) Orthogonal CT slices from three different perspectives. These images are derived from the sample shown in Fig. 2a and in Table 1 (normal mouse). Conditions for the data collection are described in Methods. A renal corpuscle is indicated by an arrowhead. (**b**) Measurement of SBE. Magnified part of (**a**) around the renal corpuscle is represented with an opacity profile along the yellow line (about 7 μm). A further magnified part of CT slice around the yellow line is shown as an inset of the line profile. In the profile, red circles represent observed values of opacity. Obtained SBE from the curve fitting (blue line) was 0.56 μm. (**c**) Parts of renal corpuscle. Presented is the single renal corpuscle marked in (**a**). Magnified CT slices are shown from left to right around distal tubule (DT; section 826th/1222), afferent arteriole (AA; section 854th/1222) with macula densa (MD), efferent arteriole (EA; section 905th/1222), and proximal tubule (PT; section 969th/1222). Relevant parts are encircled by yellow broken lines. Putative nuclei are visible in all parts as white dots with sizes in the range of 3–5 μm; some of them were indicated by arrowheads. Scale bars: 5 μm.

We observed unstained/paraffin-embedded mouse renal biopsies using a laboratory-based X-ray microscope. To improve the low contrast in X-ray absorption, paraffin-mediated contrast enhancement was applied according to the procedure described in Methods. The cylindrical biopsy piece, with a diameter of about 0.5 mm, was placed on the sample stage of the microscope and irradiated by 8 keV rotating-anode X-rays from a Cu-target for 19.4 h to collect projection images for CT reconstruction (Fig. 2). The reconstructed CT image successfully provided cellular-level spatial resolutions in all directions and high paraffin-sample CNRs (Fig. 3a, b, Table 1), which allowed us to identify major components of a renal corpuscle from a normal mouse, including afferent/efferent arterioles, proximal/distal tubules, and macula densa (Fig. 3c). As pointed out in a previous report using synchrotron X-ray CT with phase contrast optics^11^, bright particles seen in renal tubules are likely to be cell nuclei, although this assignment should be confirmed elsewhere by a rigorous method. In the present study, these spherical putative nuclei with sizes in the range of 3–5 μm were also observed in other components of the nephron.

**Table 1:** Observation statistics.

Sample	Transmission^a^	SBE (μm)^b^	CNR^c^	
			Air-paraffin	Paraffin-sample
Normal	0.828	0.63 ± 0.06	14.0 ± 0.5	8.0 ± 0.4
Disease model	0.824	0.67 ± 0.08	12.2 ± 1.0	8.7 ± 0.6

A value with range denotes an average with 95% confidence interval from five independent measurements.

^a^Calculated value from the opacity histogram of a projection image at 0° of each data set; the peak opacity of sample was divided by the peak opacity of air.

^b^Size of blurring at edges of paraffin-sample boundary; it was obtained based on Eq. 1 from a 7 μm line profile.

^c^Calculated value from an opacity comparison between two selected regions (15 pixels × 15 pixels) in CT slices; indicated materials were compared based on Eq. 2.

### Segmentation and 3D rendering of renal corpuscle

**Figure 4:**
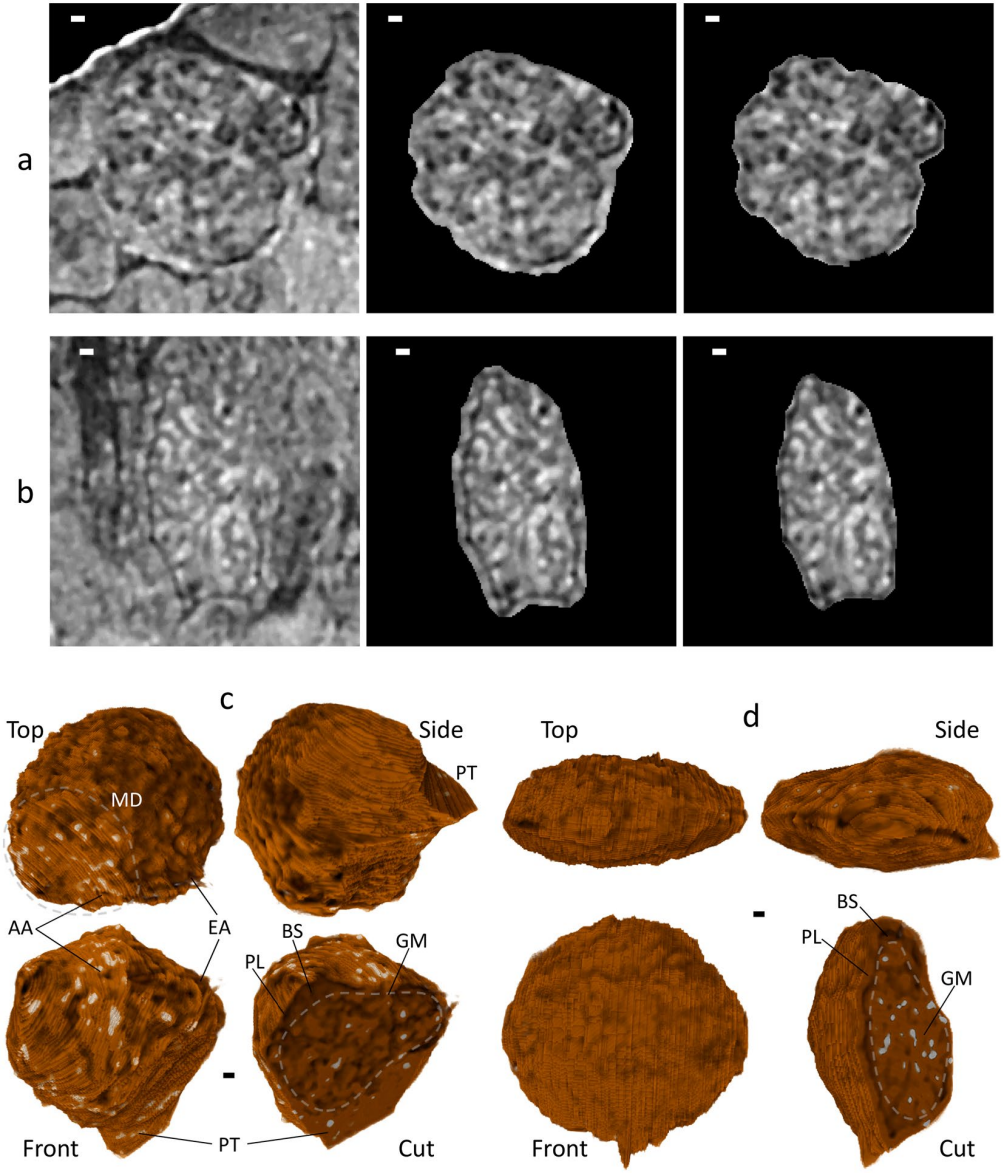
Segmentation and 3D rendering of renal corpuscle. Scale bars: 5 μm. Procedure of segmentation for (**a**) normal mouse (section 896th/1222) and from (**b**) disease model mouse (section 512nd/1222). The procedure is shown in chronological order: original CT slice (left), segmented renal corpuscle (center), and segmented glomerulus (right). The segmentation was performed manually using the program *ImageJ*^33^ for each CT slice. 3D rendering of renal corpuscle from (**c**) normal mouse and from (**d**) disease model mouse. A 3D rendering model was produced from CT slices using the program *Drishti*^34^. Presented are three models from orthogonal perspectives (front, top, side) and a cut model from similar perspective as that of the front model. Important parts are labeled: glomerulus (GM), Bowman’s space (BS), parietal layer (PL), afferent arteriole (AA), efferent arteriole (EA), macula densa (MD), proximal tubule (PT). Some parts could not be identified for the disease model mouse. Clear, brown and white colorings indicate low-, medium-, and high-density regions, respectively. Parallel lines on the model surface are artifacts due to a technical limitation of the manual segmentation.

To characterize the kidney structure in the CT image, we tried manual segmentation of a single renal corpuscle from a normal mouse. The renal corpuscle and the glomerulus were delineated on each CT section (Fig. 4a, Supplementary Figs. S2, S3) to reconstruct an isolated 3D image. Then, the isolated renal corpuscle was represented as a 3D rendering model to grasp its whole perspective (Fig. 4c, Supplementary Video S1). The normal renal corpuscle is roughly spherical in shape with a diameter of about 100 μm. It is composed of the parietal layer, Bowman’s space, and the glomerulus, and is surrounded by peripheral structures, including afferent/efferent arterioles, proximal/distal tubules, and macula densa. Spatial relationships between these parts were clarified. The parietal layer contains squamous epithelium cells and connects to the proximal tubule in the urinary pole and contacts with the afferent/efferent arterioles in the vascular pole. The Bowman’s space was obvious only in a major part shown in the rendering model, and the other minor parts could not be distinguished from glomerular regions. Inside the glomerulus, low-density regions probably representing capillary lumens were continuous in many places, although a complete tracing of them was unsuccessful due to the uncertainty in Bowman’s space.

To examine whether the present 3D method can provide useful information about kidney diseases, we observed another renal biopsy sample from a disease model mouse (Fig. 4b, d, Supplementary Figs. S4, S5, Supplementary Video S2). In contrast with the overall spherical shape of the normal one, the disease-model renal corpuscle looks like an oblate spheroid, with major and minor diameters of about 110 and 55 μm, respectively; generality of this phenomenon should be examined further elsewhere. It is noteworthy that this overall morphological change is recognized clearly in the 3D rendering, which may be an advantage of the method of X-ray microscopy because the overall shape is not necessarily clear in conventional 2D methods that depend upon the orientation of sectioning. Regarding constituents of the renal corpuscle, peripheral structures could not be found, although the parietal layer, Bowman’s space, and the glomerulus were identified. The interior of the glomerulus looks more crowded than the normal one. The low-density regions are fragmented and look smaller, and the bright particles in the other regions look denser. These characteristics may be relevant to pathological changes caused by disease.

### Volumetric analysis

**Figure 5:**
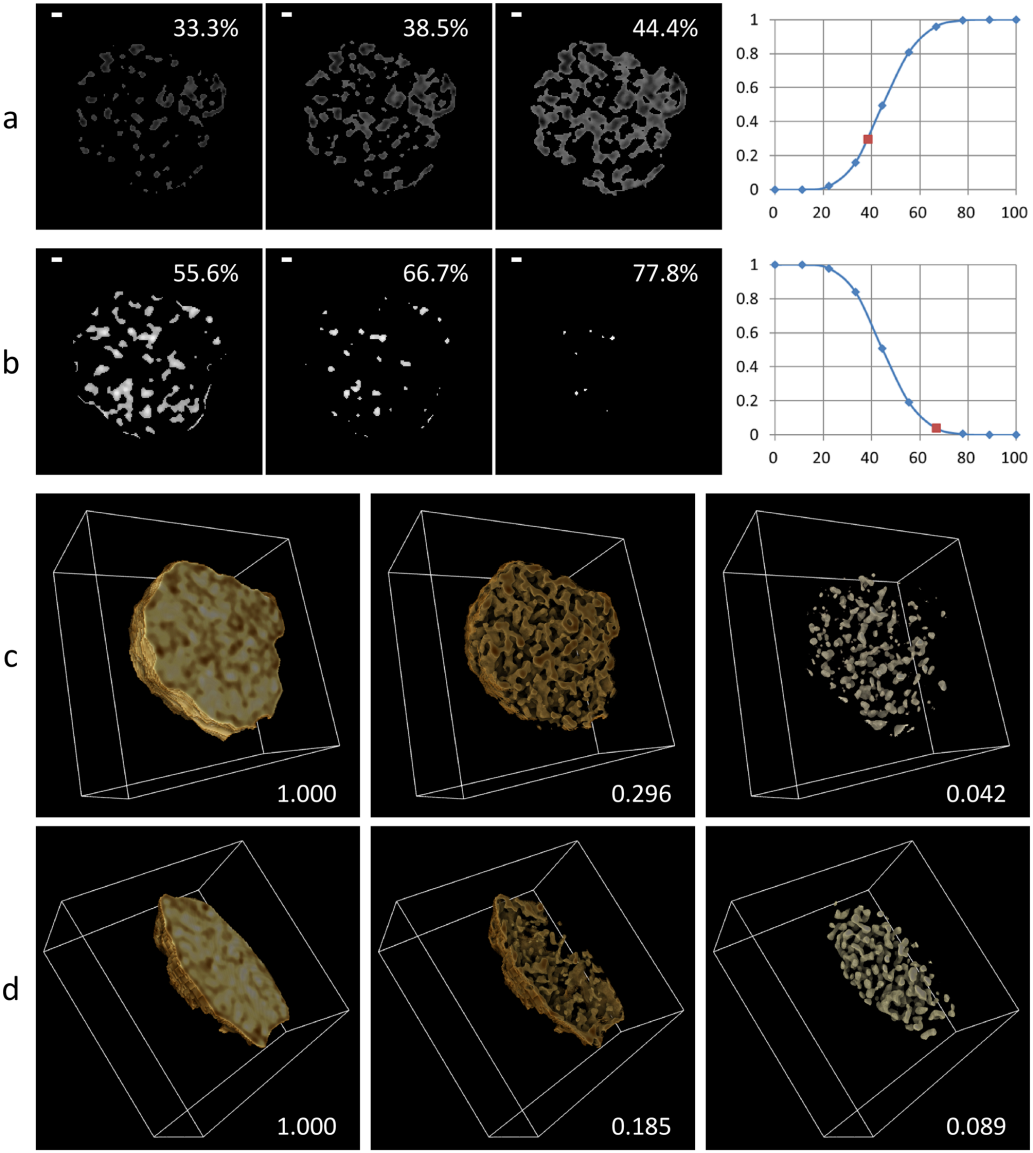
Volumetric analysis of glomerulus. This figure corresponds to Table 2. (**a**,**b**) Preliminary analysis using a CT slice with segmented regions of a glomerulus from normal mouse. Scale bars: 5 μm. The glomerulus regions were subdivided into (**a**) low-density regions including putative vessels and (**b**) high-density regions including putative nuclei, based on a relative threshold (RT) value indicated (left three panels), using the program *ImageJ*^33^. See Methods for the definition of RT value. Ratio of selected voxels was plotted versus RT (right panel). Determined suitable RT values for the subregions are indicated by red squares: 38.5% for the low-density regions and 66.7% for the high-density regions. 3D rendering of subdivided glomerulus from (**c**) normal mouse and from (**d**) disease model mouse. The subdivided CT slices were reconstructed using the program *Drishti*^34^. Original CT image (left), subdivided low-density regions (center), and subdivided high-density regions (right) are shown with calculated ratio of non-zero volume. Color code used was a linear gradient from brown to white indicating the lowest and the highest opacities, respectively. Box size: (107 × 107 × 56) μm^3^ for (**c**) and (107 × 107 × 53) μm^3^ for (**d**).

As an application of laboratory-based X-ray microscopy, we tried a volume measurement of the glomerulus to quantify the structural changes observed (Fig. 5, Table 2). In this method, the volume of an object is calculated by counting voxels with opacities satisfying a defined threshold condition (see Methods in detail). First, volumes of the components were measured by counting non-zero voxels (Table 2). Total volumes of renal corpuscles from normal and disease model mice were calculated as 2.8 × 10^5^ μm^3^ and 1.9 × 10^5^ μm^3^, respectively. The glomerulus occupies 63–71% of the total volume. Bowman’s space seems larger in the disease-model renal corpuscle. Then, the threshold conditions to extract low- and high-density regions of the glomerulus were searched on a CT slice from the normal renal corpuscle (Fig. 5a, b). Measurement of low-density regions aims to evaluate the glomerular vessel content (volume of vessel lumen) which reflects an important diagnostic factor “vessel abnormalities in glomerulosclerosis^2^.” Because glomerulosclerosis is defined as a deposition of collagen within the glomerulus, and this collagen deposition makes the vessel narrow. We searched for the threshold conditions and found that 38.5% of the relative threshold could extract the most continuous and the least overlapping voids, probably representing lumens of the glomerular capillary. On the other hand, measurement of high-density regions aims to evaluate glomerular cell deposition which reflects another important diagnostic factor “glomerular hypercellularity^2^.” We searched for threshold conditions and found that 66.7% of the relative threshold could extract the least overlapping and the largest particles, probably representing the putative nuclei of the glomerular cells.

**Table 2:** Volumetric analysis of glomerulus.

Regions^a^	Volume (μm^3^)^b^	Ratio versus		RT (%)^c^	Opacity	
		RC	GM		Mean^d^	Range
**Normal**						
RC	276,300	1	–	–	131.1 (14.7)	68–192
GM	197,200	0.714	1	–	128.4 (14.0)	76–192
PL	73,600	0.266	–	–	140.5 (11.2)	87–192
BS	5400	0.020	–	–	106.4 (10.4)	68–164
LDR	58,300	–	0.296	38.5	112.4 (6.4)	76–120
HDR	8200	–	0.042	66.7	159.9 (5.6)	154–192
**Disease model**						
RC	189,600	1	–	–	103.2 (11.8)	66–149
GM	119,100	0.628	1	–	106.6 (10.4)	66–149
PL	43,400	0.229	–	–	104.9 (8.4)	71–141
BS	27,100	0.143	–	–	86.0 (6.3)	66–138
LDR	220,00	–	0.185	38.1	92.1 (4.5)	66–97
HDR	106,00	–	0.089	66.7	126.3 (3.8)	122–149

This table corresponds to Fig. 5.

^a^Abbreviations used are: renal corpuscle (RC), glomerulus (GM), parietal layer (PL), Bowman’s space (BS), low-density regions in the glomerulus (LDR), high-density regions in the glomerulus (HDR).

^b^Multiplied value of the voxel volume (0.154 μm^3^) by the number of voxels.

^c^Relative threshold calculated based on Eq. 3.

^d^Population standard deviation is given in parenthesis.

To measure the volume of regions of interest, the threshold conditions determined were applied to the CT images of segmented glomeruli, both from the normal and the disease-model mice (Fig. 5c, d, Table 2). The continuity/overlapping of low-density regions and the size/overlapping of high-density particles were comparable to those on the CT slice used for the threshold determination. As a result, about a 40% decrease in low-density regions and about a 110% increase in high-density particles by disease were observed. This result agrees well with the known pathology of kidney diseases^2^ due to the vessel abnormalities in glomerulosclerosis and the glomerular hypercellularity, thereby suggesting the potential for cellular-level 3D observation using laboratory-based X-ray microscopy for more effective diagnosis of human renal biopsies. However, because this result is based only on one pair of renal corpuscles, its generality should be confirmed elsewhere using many renal corpuscles from various organisms. In addition, it should be noted that voxels with exceptional opacity values due to certain artifacts will disturb the extraction, which can be resolved by median filtering for instance; there were no such artifacts in the present case.

## Discussion

In this study, we report the observation of an unstained/paraffin-embedded mouse kidney using a laboratory-based X-ray microscope. Paraffin-mediated contrast enhancement was essential to achieve cellular-level imaging. Using an X-ray energy of 8 keV may be suitable for cellular-level observation of a paraffin-embedded cylindrical renal biopsy with a diameter of 0.5–1.0 mm, because the attenuation length of dehydrated soft tissue and that of paraffin-embedded soft tissue are estimated to be 470 μm and 1160 μm, respectively (Fig. 1a), and because the 1% contrast length between paraffin and dehydrated soft tissue is calculated to be 5.6 μm (Fig. 1b). Importantly, this nondestructive method presents CT opacities reflecting elemental composition and density of unstained tissues, thereby allowing an unbiased interpretation of kidney structure. Noteworthy, putative nuclei were observed at various parts of the nephron. It is conceivable that the stronger X-ray absorption of nucleic acids provides higher opacity of cell nuclei in the CT image compared to other parts of tissue. There may be other life sciences applications for this technique. The range of X-ray energies acceptable for cellular- to subcellular-level observations of bio-specimens with paraffin-mediated contrast enhancement would be 4–12 keV. In addition, the contrast “negative” is true in wider energy range of 1–30 keV (Fig. 1a), suggesting further potential of this approach.

To examine the medical applicability of this method, a pair of single renal corpuscles—one from a normal mouse and one from a disease model mouse—were segmented and compared by making 3D rendering models. The 3D rendering clearly revealed an overall morphological change from a sphere to an oblate spheroid upon disease, thereby suggesting an advantage of X-ray microscopy compared to other 2D methods. Furthermore, volumetric analysis revealed remarkable volume changes among the glomerular constituents upon disease. The changes were consistent with the known pathology of kidney diseases. Therefore, the observation of renal corpuscles at cellular-level resolutions using laboratory-based X-ray microscopy with paraffin-mediated contrast enhancement may have a potential for more effective on-site diagnosis of human renal biopsies. Although manual segmentation was adopted in this work to extract renal corpuscles, automatic segmentation using a technique such as the active contour method^30^ may be available in the near future; automatic segmentation at a much lower CNR—around 1.5—was reported in the case of maize embryo^31^.

In addition to the contrast enhancement ability, another notable advantage of paraffin may be its compatibility with optical microscopy, because the use of unstained/paraffin-embedded sample is established in histological observation protocols. Thus, the same unstained/paraffin-embedded sample can be shared between optical and X-ray microscopies without changing standard procedures for sample preparation. Furthermore, the nondestructive characteristic of laboratory-based X-ray CT may be suitable for its complementary use with other observation modalities, including electron microscopy. This multi-modality correlative microscopy will allow a wide range of visualization from millimeter to nanometer scales, thereby contributing significantly to structural biology and medicine in the future. However, direct comparison of images between X-ray microscopy and the conventional 2D modalities is still difficult to date. Further improvements in resolution/contrast of laboratory-based X-ray microscopy will be required to realize the correlative microscopy.

## Methods

### Mouse kidney material

Experiments were performed on 6- to 8-week-old male C57BL/6 mice that were housed under controlled environmental conditions and maintained with standard food and water. Renal ablation was performed in a procedure similar to the one described previously^32^. Briefly, the anesthetized mice were placed on a heating pad to maintain a constant body temperature (37 °C). Right flank incisions were made, and two-thirds of the mass of the left kidney was ablated. Seven days later, the right kidney was removed. After the renal ablation, the flanks were closed in two layers with silk sutures. The kidney samples from 5/6 nephrectomy mice were used 8 weeks after ablation. All procedures used in the animal experiments complied with the standards set out in the guidelines for the care and use of laboratory animals of Kanazawa Medical University and were approved by the Research Center Ethics committee for Animal Life Science of Kanazawa Medical University (Approval number: 2020–25). All animals were also maintained and used under the ARRIVE guidelines.

Paraffin-embedded mouse kidney tissues prepared by standard procedures were used for analysis. Procedures for preparation of paraffin-embedded mouse kidney are as follows. Kidney samples from normal and 5/6 nephrectomy mice were taken using biopsy needles (ACECUT, TSK Laboratory, Tochigi, Japan). The kidney specimens were fixed in enough (more than 10 times the tissue volume) 10% buffered formalin fixatives for over 24 h at room temperature. For dehydration of tissues, a series of ethanol (70, 80, 90, 95, and 100% ethanol) and xylene were used. Finally, the tissues were embedded into paraffin blocks. For the pathological analysis, the paraffin blocks were cut at 4 μm and stained with periodic acid Schiff’s reagent (PAS). The PAS-stained tissues were digitally captured (Nano Zoomer C9600-03, Hamamatsu Photonics K.K, Hamamatsu, Japan), and used for further digital image analysis.

### Direct mounting

The unstained/paraffin-embedded mouse renal biopsy was processed for the X-ray microscopy observation as follows. First, a paraffin block containing a deformed cylindrical biopsy specimen with a diameter of about 0.5 mm (0.2–0.6 mm depending on location) was trimmed to remove excess paraffin around the specimen using a disposable razor or a diamond wire saw (Musashino Denshi Inc., Tokyo, Japan; CS-203). Then, the trimmed paraffin block of about (1.5 × 1.5 × 3) mm^3^ in size was put on the top plane of a cylindrical metal stick (diameter of 3 mm and height of 55 mm), and the bottom of the stick was heated with a water bath (disposable paper cup) at about 90 °C for less than 5 min so the paraffin in the sample melted. The specimen was protected against vapor from the hot water by penetrating the metal stick through a sheet of aluminum foil that covered the paper cup. Cellophane tape was used to attach the stick to the aluminum foil. After removal of the water bath and just before beginning the re-solidification of the paraffin, the biopsy piece was placed on the metal stick so that the long axes of the specimen and the metal stick were aligned. The alignment may be adjusted by touching with the tip of a disposable pipette chip (Molecular Bioproducts, Inc., CA, USA; 0.1–10 μl, cat#103) or with a hair shaft. After solidification was completed, the metal stick was fixed on a cylindrical metal jig (diameter of 12 mm and height of 12 mm) using a hexagon socket setscrew. This melting-solidifying treatment aims a free paraffin removal that reduces extra X-ray absorption and also aims a sample surface smoothing that prevents artifacts during CT reconstruction.

### Data collection

The metal jig on which the renal biopsy was mounted was placed on the sample stage of an X-ray microscope apparatus, nano3DX, (Rigaku Corporation, Tokyo, Japan) with a scintillator-based lens and with a 16-bit 3296 × 2472 (5.5 μm/pixel) CCD detector. To reduce the influence of a drifting light source, a quasi-parallel X-ray beam setting was adopted, where the sample-to-detector distance was set much shorter than the source-to-sample distance (260 mm). The sample was scanned by laboratory 8 keV X-rays from a rotating anode Cu-target (40 kV, 30 mA; spot size of 70 μm; unfiltered characteristic X-ray) to collect 1700 projection images with a pixel size of 0.54 μm (L0270 (20x) lens, bin 2) in step scan mode with an exposure of 40 s per frame (19.4 h in total) and with a sample-to-detector distance of 4 mm. The field-of-view used was 0.88 mm × 0.66 mm. Conventional median/Gaussian-based noise filter (denoise; radius of 1 pixel for median, radius of 1 sigma for Gaussian) was applied.

### Image processing and analysis

CT reconstruction (8 bit) was performed based on a conventional filtered-back-projection method (convolution back-projection). Segmentation of a renal corpuscle from 200 CT slices (801st–1000th/1222; z = 429.1–536.3 μm; trimmed as a square 200 pixels on a side) for the normal mouse or from 220 CT slices (391st–610th/1222; z = 209.8–327.2 μm; trimmed as a square of 200 pixels on a side) for the disease model mouse was performed manually as described^19^, except for using the polygon selection tool of *ImageJ*^33^ and making a mask. For each CT slice, the boundary of the renal corpuscle was delineated and a mask for the selected area was prepared using the “Clear” tool of *ImageJ*. Then, the mask and the original CT slice was multiplied to produce isolated regions of the renal corpuscle using the “Image calculator” tool. Consecutive slices with the isolated regions were combined to reconstruct a 3D model. The boundary was redrawn when positional differences were detected. The 3D rendering was performed using the program *Drishti*^34^.

To evaluate the spatial resolution of a CT image, an index referred to as “size of blurring at edges (SBE)” was defined and measured. A logistic curve-fitting technique^35^ against a line opacity profile across a well-defined edge in the image was used to obtain SBE, as described^19^:

1 $$y = A - \frac{A - B}{{1 + \left( {{\raise0.7ex\hbox{$x$} \!\mathord{\left/ {\vphantom {x C}}\right.\kern-\nulldelimiterspace} \!\lower0.7ex\hbox{$C$}}} \right)^{D} }}$$

where variables *x* and *y* represent the position and the value of a voxel, respectively, and the parameters *A* to *D* represent the maximum asymptote value, the minimum asymptote value, the inflection position, and the Hill’s slope, respectively. The distance between two positions giving values *A*-0.25(*A*-*B*) and *A*-0.75(*A*-*B*) was defined as SBE; only distances greater than voxel size were accepted. This SBE has the same definition as “spatial resolution” in our previous work^19^; the notation was changed to avoid confusion. According to ASTM E1695-20 that describes international standard for CT performance^36^, SBE may be relevant to “basic spatial resolution” that is greater than voxel size. Contrast-to-noise ratio (CNR) between two regions of CT slices was calculated as described^19^:

2 $$CNR = \frac{{\left| {\mu_{1} - \mu_{2} } \right|}}{{\sqrt {\sigma_{1}^{2} + \sigma_{2}^{2} } }}$$

where *μ*_1_ and *μ*_2_ represent the average opacity values of the two regions and *σ*_1_ and *σ*_2_ represent their corresponding standard deviations. This CNR has the same definition as “signal-to-noise ratio (SNR)” in our previous work^19^; we changed the notation considering a terminological preference. For each subregion (air, paraffin, sample) to be used for the CNR measurement in a CT slice, the value of the corresponding peak in an opacity histogram of the CT slice was used as a guide to select the region. To take possible anisotropy into account, objects for SBE/CNR analyses were selected evenly from three orthogonal CT slices. The volumetric analysis was performed using the “Threshold” tool of *ImageJ*. The relative threshold of for a relevant CT region was calculated as:

3 $$RT = 100 \times \frac{{\left( {I_{thr} - I_{min} } \right)}}{{\left( {I_{max} - I_{min} + 1} \right)}}$$

where *I*_thr_, *I*_min_, and *I*_max_ represent the threshold, the minimum, and the maximum opacities in the CT region, respectively. A voxel in the low-density subregions has an opacity less than *I*_thr_, whereas that in the high-density subregions has an opacity equal to or greater than *I*_thr_.

## Supplementary Information

Supplementary Information 1.

Supplementary Information 2.

Supplementary Information 3.

Supplementary Information 4.

Supplementary Information 5.

Supplementary Information 6.

Supplementary Video 1.

Supplementary Video 2.
