# Supplementary figures and images for: Nondestructive cellular-level 3D observation of mouse kidney using laboratory-based X-ray microscopy with paraffin-mediated contrast enhancement (part 1 of 9)
Source: Sci Rep. 2022 Jun 8;12:9436. doi: 10.1038/s41598-022-13394-9 (PMC9177607; doi:10.1038/s41598-022-13394-9)

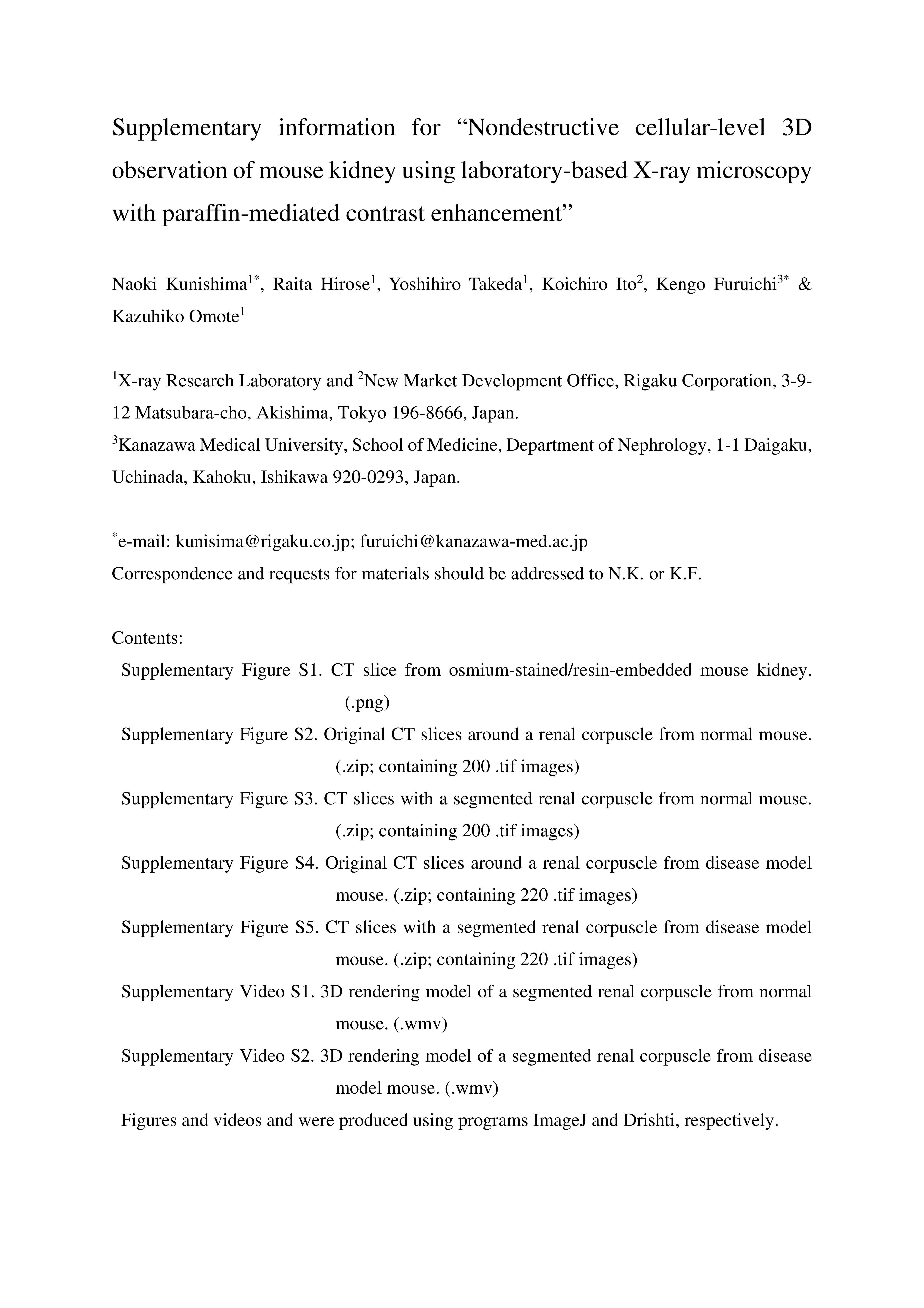

Supplement: Supplementary file 1 — Supplementary Information 1. [file 41598_2022_13394_MOESM1_ESM.png]

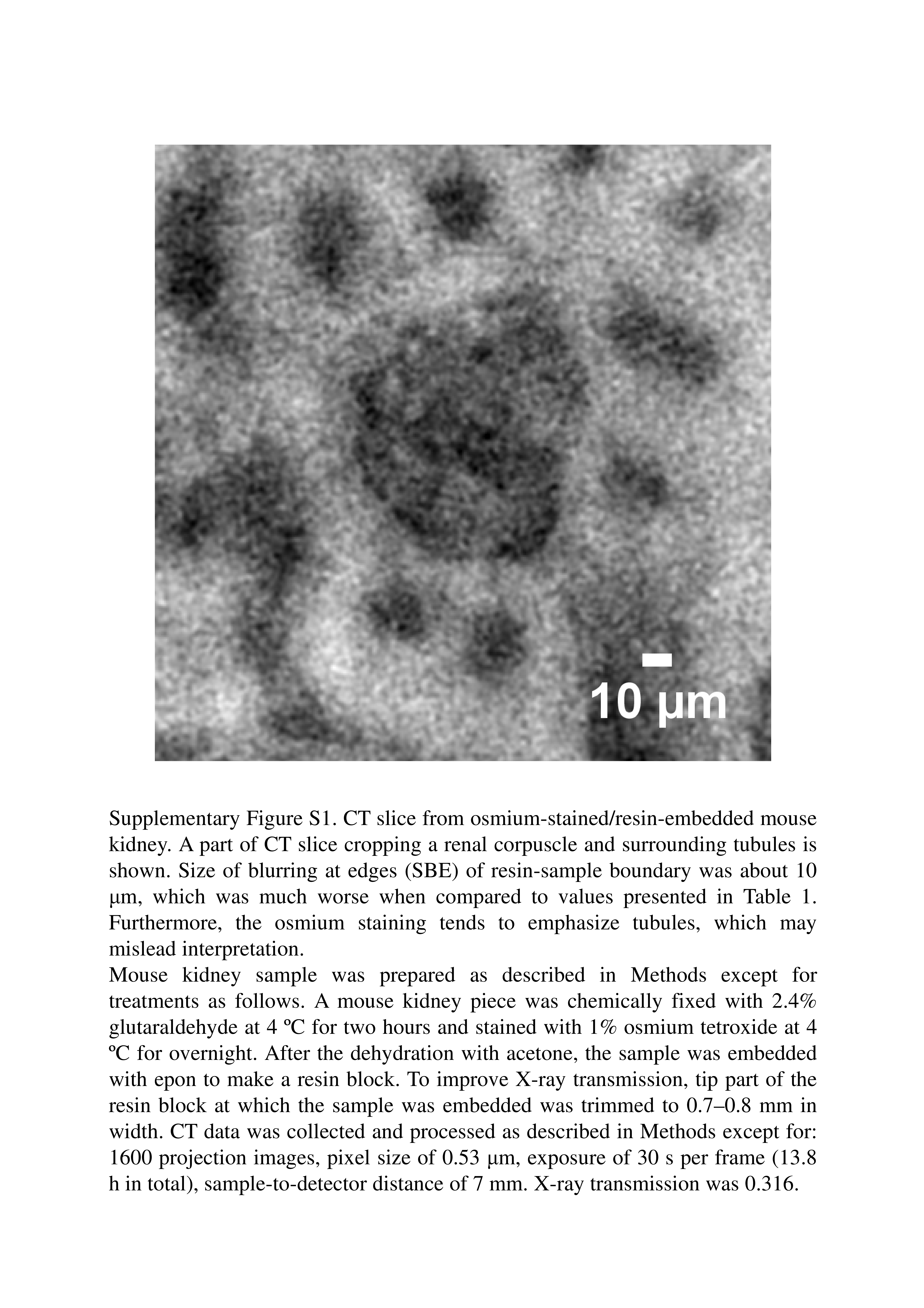

Supplement: Supplementary file 2 — Supplementary Information 2. [file 41598_2022_13394_MOESM2_ESM.png]

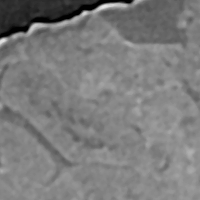

Supplement: Supplementary file 3 — Supplementary Information 3. [file 41598_2022_13394_MOESM3_ESM.zip › Supplementary Figure S2/Supplementary_Figure_S2_001.tif]

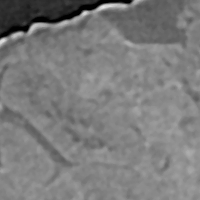

Supplement: Supplementary file 3 — Supplementary Information 3. [file 41598_2022_13394_MOESM3_ESM.zip › Supplementary Figure S2/Supplementary_Figure_S2_002.tif]

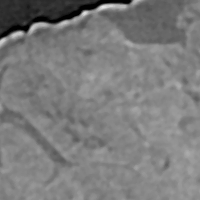

Supplement: Supplementary file 3 — Supplementary Information 3. [file 41598_2022_13394_MOESM3_ESM.zip › Supplementary Figure S2/Supplementary_Figure_S2_003.tif]

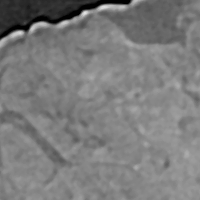

Supplement: Supplementary file 3 — Supplementary Information 3. [file 41598_2022_13394_MOESM3_ESM.zip › Supplementary Figure S2/Supplementary_Figure_S2_004.tif]

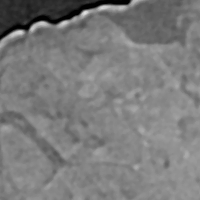

Supplement: Supplementary file 3 — Supplementary Information 3. [file 41598_2022_13394_MOESM3_ESM.zip › Supplementary Figure S2/Supplementary_Figure_S2_005.tif]

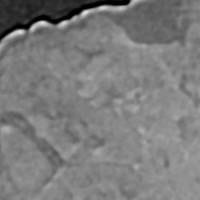

Supplement: Supplementary file 3 — Supplementary Information 3. [file 41598_2022_13394_MOESM3_ESM.zip › Supplementary Figure S2/Supplementary_Figure_S2_006.tif]

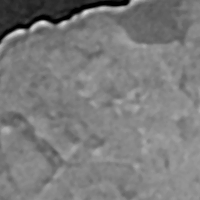

Supplement: Supplementary file 3 — Supplementary Information 3. [file 41598_2022_13394_MOESM3_ESM.zip › Supplementary Figure S2/Supplementary_Figure_S2_007.tif]

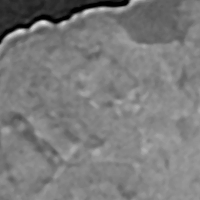

Supplement: Supplementary file 3 — Supplementary Information 3. [file 41598_2022_13394_MOESM3_ESM.zip › Supplementary Figure S2/Supplementary_Figure_S2_008.tif]

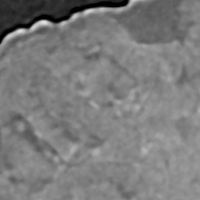

Supplement: Supplementary file 3 — Supplementary Information 3. [file 41598_2022_13394_MOESM3_ESM.zip › Supplementary Figure S2/Supplementary_Figure_S2_009.tif]

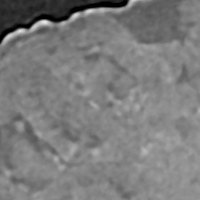

Supplement: Supplementary file 3 — Supplementary Information 3. [file 41598_2022_13394_MOESM3_ESM.zip › Supplementary Figure S2/Supplementary_Figure_S2_010.tif]

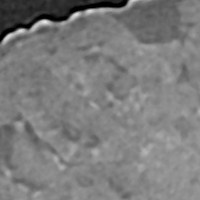

Supplement: Supplementary file 3 — Supplementary Information 3. [file 41598_2022_13394_MOESM3_ESM.zip › Supplementary Figure S2/Supplementary_Figure_S2_011.tif]

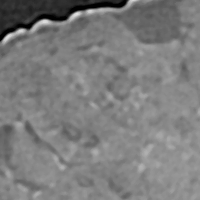

Supplement: Supplementary file 3 — Supplementary Information 3. [file 41598_2022_13394_MOESM3_ESM.zip › Supplementary Figure S2/Supplementary_Figure_S2_012.tif]

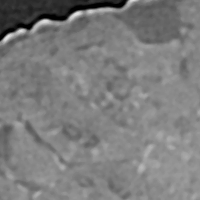

Supplement: Supplementary file 3 — Supplementary Information 3. [file 41598_2022_13394_MOESM3_ESM.zip › Supplementary Figure S2/Supplementary_Figure_S2_013.tif]

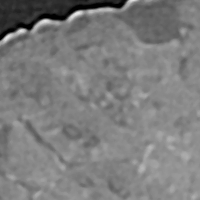

Supplement: Supplementary file 3 — Supplementary Information 3. [file 41598_2022_13394_MOESM3_ESM.zip › Supplementary Figure S2/Supplementary_Figure_S2_014.tif]

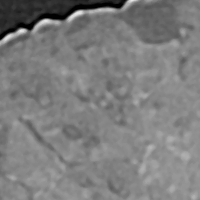

Supplement: Supplementary file 3 — Supplementary Information 3. [file 41598_2022_13394_MOESM3_ESM.zip › Supplementary Figure S2/Supplementary_Figure_S2_015.tif]

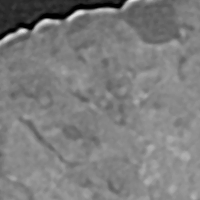

Supplement: Supplementary file 3 — Supplementary Information 3. [file 41598_2022_13394_MOESM3_ESM.zip › Supplementary Figure S2/Supplementary_Figure_S2_016.tif]

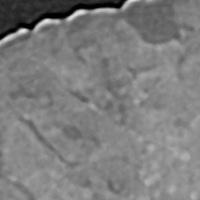

Supplement: Supplementary file 3 — Supplementary Information 3. [file 41598_2022_13394_MOESM3_ESM.zip › Supplementary Figure S2/Supplementary_Figure_S2_017.tif]

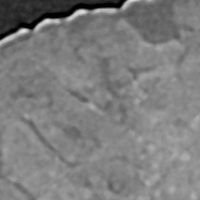

Supplement: Supplementary file 3 — Supplementary Information 3. [file 41598_2022_13394_MOESM3_ESM.zip › Supplementary Figure S2/Supplementary_Figure_S2_018.tif]

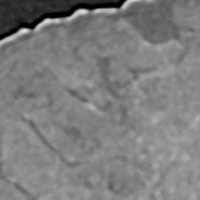

Supplement: Supplementary file 3 — Supplementary Information 3. [file 41598_2022_13394_MOESM3_ESM.zip › Supplementary Figure S2/Supplementary_Figure_S2_019.tif]

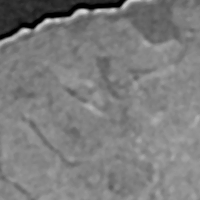

Supplement: Supplementary file 3 — Supplementary Information 3. [file 41598_2022_13394_MOESM3_ESM.zip › Supplementary Figure S2/Supplementary_Figure_S2_020.tif]

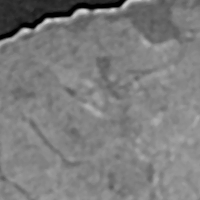

Supplement: Supplementary file 3 — Supplementary Information 3. [file 41598_2022_13394_MOESM3_ESM.zip › Supplementary Figure S2/Supplementary_Figure_S2_021.tif]

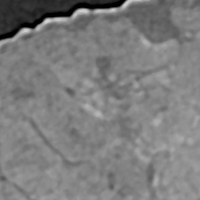

Supplement: Supplementary file 3 — Supplementary Information 3. [file 41598_2022_13394_MOESM3_ESM.zip › Supplementary Figure S2/Supplementary_Figure_S2_022.tif]

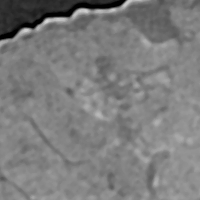

Supplement: Supplementary file 3 — Supplementary Information 3. [file 41598_2022_13394_MOESM3_ESM.zip › Supplementary Figure S2/Supplementary_Figure_S2_023.tif]

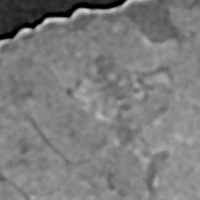

Supplement: Supplementary file 3 — Supplementary Information 3. [file 41598_2022_13394_MOESM3_ESM.zip › Supplementary Figure S2/Supplementary_Figure_S2_024.tif]

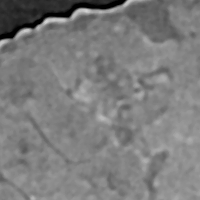

Supplement: Supplementary file 3 — Supplementary Information 3. [file 41598_2022_13394_MOESM3_ESM.zip › Supplementary Figure S2/Supplementary_Figure_S2_025.tif]

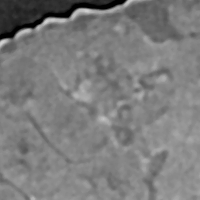

Supplement: Supplementary file 3 — Supplementary Information 3. [file 41598_2022_13394_MOESM3_ESM.zip › Supplementary Figure S2/Supplementary_Figure_S2_026.tif]

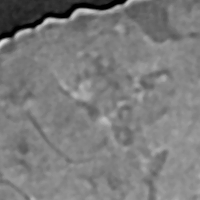

Supplement: Supplementary file 3 — Supplementary Information 3. [file 41598_2022_13394_MOESM3_ESM.zip › Supplementary Figure S2/Supplementary_Figure_S2_027.tif]

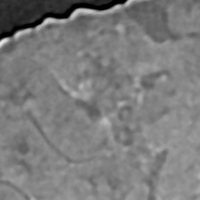

Supplement: Supplementary file 3 — Supplementary Information 3. [file 41598_2022_13394_MOESM3_ESM.zip › Supplementary Figure S2/Supplementary_Figure_S2_028.tif]

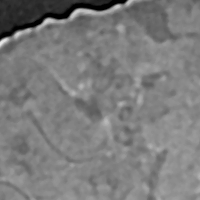

Supplement: Supplementary file 3 — Supplementary Information 3. [file 41598_2022_13394_MOESM3_ESM.zip › Supplementary Figure S2/Supplementary_Figure_S2_029.tif]

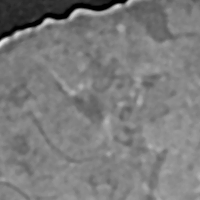

Supplement: Supplementary file 3 — Supplementary Information 3. [file 41598_2022_13394_MOESM3_ESM.zip › Supplementary Figure S2/Supplementary_Figure_S2_030.tif]

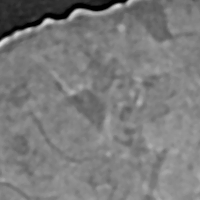

Supplement: Supplementary file 3 — Supplementary Information 3. [file 41598_2022_13394_MOESM3_ESM.zip › Supplementary Figure S2/Supplementary_Figure_S2_031.tif]

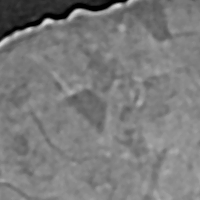

Supplement: Supplementary file 3 — Supplementary Information 3. [file 41598_2022_13394_MOESM3_ESM.zip › Supplementary Figure S2/Supplementary_Figure_S2_032.tif]

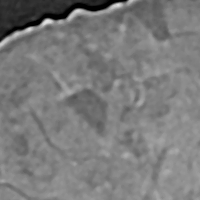

Supplement: Supplementary file 3 — Supplementary Information 3. [file 41598_2022_13394_MOESM3_ESM.zip › Supplementary Figure S2/Supplementary_Figure_S2_033.tif]

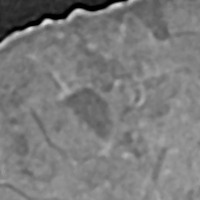

Supplement: Supplementary file 3 — Supplementary Information 3. [file 41598_2022_13394_MOESM3_ESM.zip › Supplementary Figure S2/Supplementary_Figure_S2_034.tif]

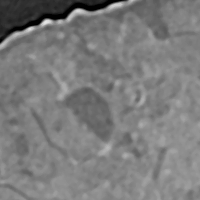

Supplement: Supplementary file 3 — Supplementary Information 3. [file 41598_2022_13394_MOESM3_ESM.zip › Supplementary Figure S2/Supplementary_Figure_S2_035.tif]

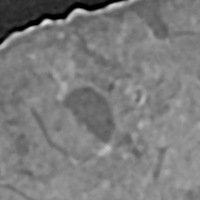

Supplement: Supplementary file 3 — Supplementary Information 3. [file 41598_2022_13394_MOESM3_ESM.zip › Supplementary Figure S2/Supplementary_Figure_S2_036.tif]

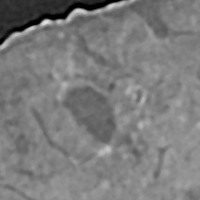

Supplement: Supplementary file 3 — Supplementary Information 3. [file 41598_2022_13394_MOESM3_ESM.zip › Supplementary Figure S2/Supplementary_Figure_S2_037.tif]

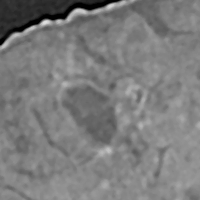

Supplement: Supplementary file 3 — Supplementary Information 3. [file 41598_2022_13394_MOESM3_ESM.zip › Supplementary Figure S2/Supplementary_Figure_S2_038.tif]

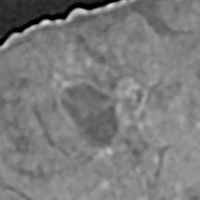

Supplement: Supplementary file 3 — Supplementary Information 3. [file 41598_2022_13394_MOESM3_ESM.zip › Supplementary Figure S2/Supplementary_Figure_S2_039.tif]

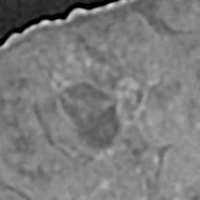

Supplement: Supplementary file 3 — Supplementary Information 3. [file 41598_2022_13394_MOESM3_ESM.zip › Supplementary Figure S2/Supplementary_Figure_S2_040.tif]

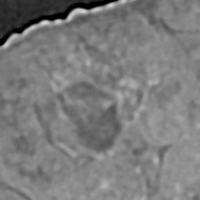

Supplement: Supplementary file 3 — Supplementary Information 3. [file 41598_2022_13394_MOESM3_ESM.zip › Supplementary Figure S2/Supplementary_Figure_S2_041.tif]

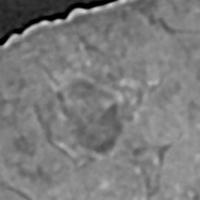

Supplement: Supplementary file 3 — Supplementary Information 3. [file 41598_2022_13394_MOESM3_ESM.zip › Supplementary Figure S2/Supplementary_Figure_S2_042.tif]

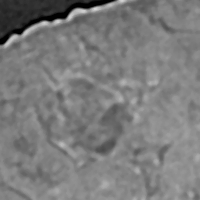

Supplement: Supplementary file 3 — Supplementary Information 3. [file 41598_2022_13394_MOESM3_ESM.zip › Supplementary Figure S2/Supplementary_Figure_S2_043.tif]

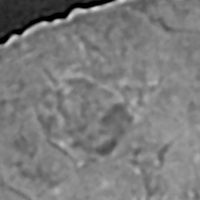

Supplement: Supplementary file 3 — Supplementary Information 3. [file 41598_2022_13394_MOESM3_ESM.zip › Supplementary Figure S2/Supplementary_Figure_S2_044.tif]

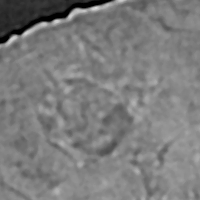

Supplement: Supplementary file 3 — Supplementary Information 3. [file 41598_2022_13394_MOESM3_ESM.zip › Supplementary Figure S2/Supplementary_Figure_S2_045.tif]

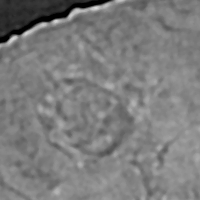

Supplement: Supplementary file 3 — Supplementary Information 3. [file 41598_2022_13394_MOESM3_ESM.zip › Supplementary Figure S2/Supplementary_Figure_S2_046.tif]

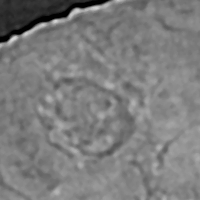

Supplement: Supplementary file 3 — Supplementary Information 3. [file 41598_2022_13394_MOESM3_ESM.zip › Supplementary Figure S2/Supplementary_Figure_S2_047.tif]

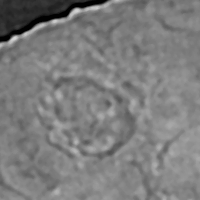

Supplement: Supplementary file 3 — Supplementary Information 3. [file 41598_2022_13394_MOESM3_ESM.zip › Supplementary Figure S2/Supplementary_Figure_S2_048.tif]

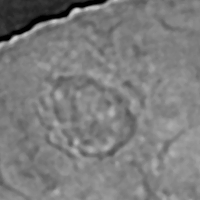

Supplement: Supplementary file 3 — Supplementary Information 3. [file 41598_2022_13394_MOESM3_ESM.zip › Supplementary Figure S2/Supplementary_Figure_S2_049.tif]

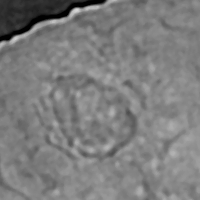

Supplement: Supplementary file 3 — Supplementary Information 3. [file 41598_2022_13394_MOESM3_ESM.zip › Supplementary Figure S2/Supplementary_Figure_S2_050.tif]

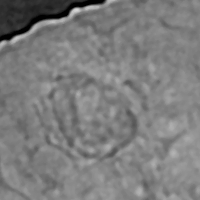

Supplement: Supplementary file 3 — Supplementary Information 3. [file 41598_2022_13394_MOESM3_ESM.zip › Supplementary Figure S2/Supplementary_Figure_S2_051.tif]

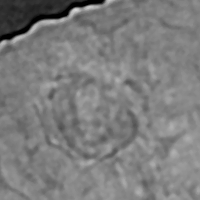

Supplement: Supplementary file 3 — Supplementary Information 3. [file 41598_2022_13394_MOESM3_ESM.zip › Supplementary Figure S2/Supplementary_Figure_S2_052.tif]

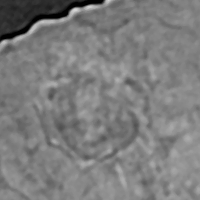

Supplement: Supplementary file 3 — Supplementary Information 3. [file 41598_2022_13394_MOESM3_ESM.zip › Supplementary Figure S2/Supplementary_Figure_S2_053.tif]

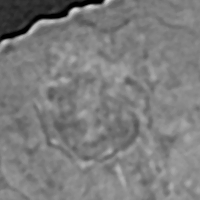

Supplement: Supplementary file 3 — Supplementary Information 3. [file 41598_2022_13394_MOESM3_ESM.zip › Supplementary Figure S2/Supplementary_Figure_S2_054.tif]

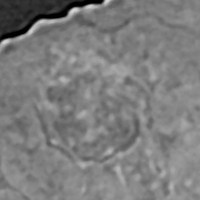

Supplement: Supplementary file 3 — Supplementary Information 3. [file 41598_2022_13394_MOESM3_ESM.zip › Supplementary Figure S2/Supplementary_Figure_S2_055.tif]

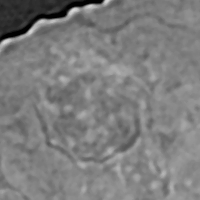

Supplement: Supplementary file 3 — Supplementary Information 3. [file 41598_2022_13394_MOESM3_ESM.zip › Supplementary Figure S2/Supplementary_Figure_S2_056.tif]

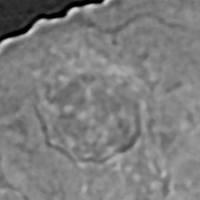

Supplement: Supplementary file 3 — Supplementary Information 3. [file 41598_2022_13394_MOESM3_ESM.zip › Supplementary Figure S2/Supplementary_Figure_S2_057.tif]

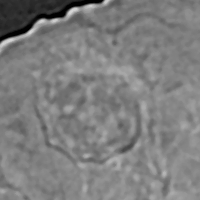

Supplement: Supplementary file 3 — Supplementary Information 3. [file 41598_2022_13394_MOESM3_ESM.zip › Supplementary Figure S2/Supplementary_Figure_S2_058.tif]

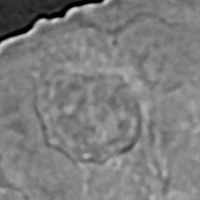

Supplement: Supplementary file 3 — Supplementary Information 3. [file 41598_2022_13394_MOESM3_ESM.zip › Supplementary Figure S2/Supplementary_Figure_S2_059.tif]

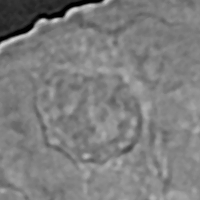

Supplement: Supplementary file 3 — Supplementary Information 3. [file 41598_2022_13394_MOESM3_ESM.zip › Supplementary Figure S2/Supplementary_Figure_S2_060.tif]

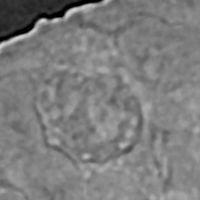

Supplement: Supplementary file 3 — Supplementary Information 3. [file 41598_2022_13394_MOESM3_ESM.zip › Supplementary Figure S2/Supplementary_Figure_S2_061.tif]

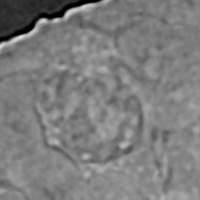

Supplement: Supplementary file 3 — Supplementary Information 3. [file 41598_2022_13394_MOESM3_ESM.zip › Supplementary Figure S2/Supplementary_Figure_S2_062.tif]

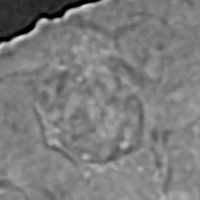

Supplement: Supplementary file 3 — Supplementary Information 3. [file 41598_2022_13394_MOESM3_ESM.zip › Supplementary Figure S2/Supplementary_Figure_S2_063.tif]

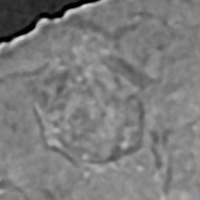

Supplement: Supplementary file 3 — Supplementary Information 3. [file 41598_2022_13394_MOESM3_ESM.zip › Supplementary Figure S2/Supplementary_Figure_S2_064.tif]

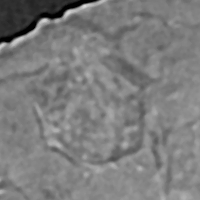

Supplement: Supplementary file 3 — Supplementary Information 3. [file 41598_2022_13394_MOESM3_ESM.zip › Supplementary Figure S2/Supplementary_Figure_S2_065.tif]

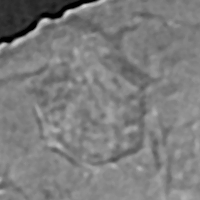

Supplement: Supplementary file 3 — Supplementary Information 3. [file 41598_2022_13394_MOESM3_ESM.zip › Supplementary Figure S2/Supplementary_Figure_S2_066.tif]

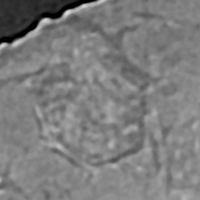

Supplement: Supplementary file 3 — Supplementary Information 3. [file 41598_2022_13394_MOESM3_ESM.zip › Supplementary Figure S2/Supplementary_Figure_S2_067.tif]

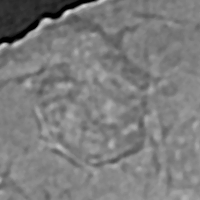

Supplement: Supplementary file 3 — Supplementary Information 3. [file 41598_2022_13394_MOESM3_ESM.zip › Supplementary Figure S2/Supplementary_Figure_S2_068.tif]

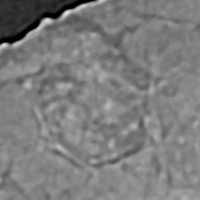

Supplement: Supplementary file 3 — Supplementary Information 3. [file 41598_2022_13394_MOESM3_ESM.zip › Supplementary Figure S2/Supplementary_Figure_S2_069.tif]

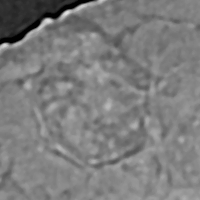

Supplement: Supplementary file 3 — Supplementary Information 3. [file 41598_2022_13394_MOESM3_ESM.zip › Supplementary Figure S2/Supplementary_Figure_S2_070.tif]

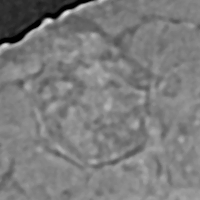

Supplement: Supplementary file 3 — Supplementary Information 3. [file 41598_2022_13394_MOESM3_ESM.zip › Supplementary Figure S2/Supplementary_Figure_S2_071.tif]

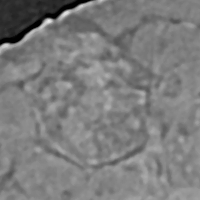

Supplement: Supplementary file 3 — Supplementary Information 3. [file 41598_2022_13394_MOESM3_ESM.zip › Supplementary Figure S2/Supplementary_Figure_S2_072.tif]

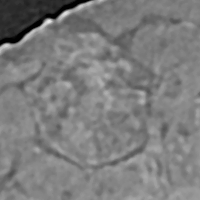

Supplement: Supplementary file 3 — Supplementary Information 3. [file 41598_2022_13394_MOESM3_ESM.zip › Supplementary Figure S2/Supplementary_Figure_S2_073.tif]

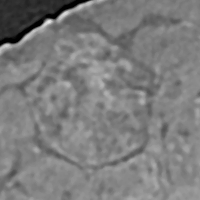

Supplement: Supplementary file 3 — Supplementary Information 3. [file 41598_2022_13394_MOESM3_ESM.zip › Supplementary Figure S2/Supplementary_Figure_S2_074.tif]

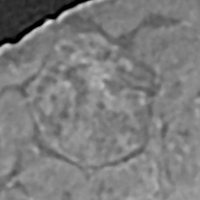

Supplement: Supplementary file 3 — Supplementary Information 3. [file 41598_2022_13394_MOESM3_ESM.zip › Supplementary Figure S2/Supplementary_Figure_S2_075.tif]

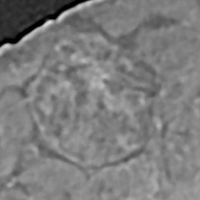

Supplement: Supplementary file 3 — Supplementary Information 3. [file 41598_2022_13394_MOESM3_ESM.zip › Supplementary Figure S2/Supplementary_Figure_S2_076.tif]

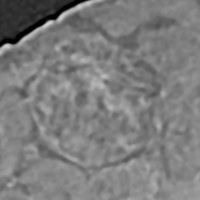

Supplement: Supplementary file 3 — Supplementary Information 3. [file 41598_2022_13394_MOESM3_ESM.zip › Supplementary Figure S2/Supplementary_Figure_S2_077.tif]

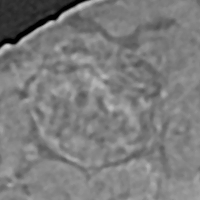

Supplement: Supplementary file 3 — Supplementary Information 3. [file 41598_2022_13394_MOESM3_ESM.zip › Supplementary Figure S2/Supplementary_Figure_S2_078.tif]

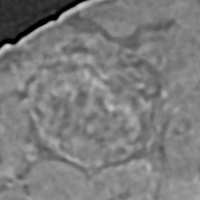

Supplement: Supplementary file 3 — Supplementary Information 3. [file 41598_2022_13394_MOESM3_ESM.zip › Supplementary Figure S2/Supplementary_Figure_S2_079.tif]

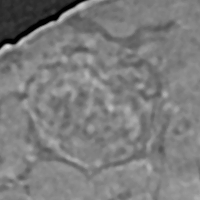

Supplement: Supplementary file 3 — Supplementary Information 3. [file 41598_2022_13394_MOESM3_ESM.zip › Supplementary Figure S2/Supplementary_Figure_S2_080.tif]

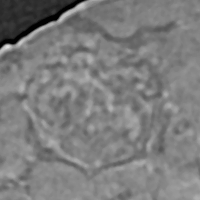

Supplement: Supplementary file 3 — Supplementary Information 3. [file 41598_2022_13394_MOESM3_ESM.zip › Supplementary Figure S2/Supplementary_Figure_S2_081.tif]

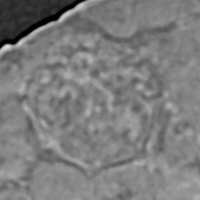

Supplement: Supplementary file 3 — Supplementary Information 3. [file 41598_2022_13394_MOESM3_ESM.zip › Supplementary Figure S2/Supplementary_Figure_S2_082.tif]

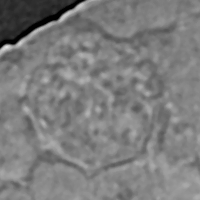

Supplement: Supplementary file 3 — Supplementary Information 3. [file 41598_2022_13394_MOESM3_ESM.zip › Supplementary Figure S2/Supplementary_Figure_S2_083.tif]

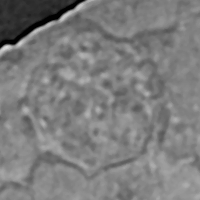

Supplement: Supplementary file 3 — Supplementary Information 3. [file 41598_2022_13394_MOESM3_ESM.zip › Supplementary Figure S2/Supplementary_Figure_S2_084.tif]

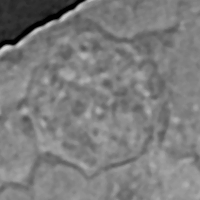

Supplement: Supplementary file 3 — Supplementary Information 3. [file 41598_2022_13394_MOESM3_ESM.zip › Supplementary Figure S2/Supplementary_Figure_S2_085.tif]

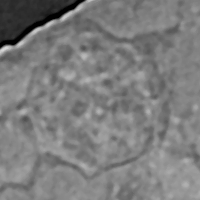

Supplement: Supplementary file 3 — Supplementary Information 3. [file 41598_2022_13394_MOESM3_ESM.zip › Supplementary Figure S2/Supplementary_Figure_S2_086.tif]

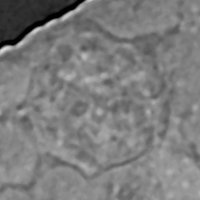

Supplement: Supplementary file 3 — Supplementary Information 3. [file 41598_2022_13394_MOESM3_ESM.zip › Supplementary Figure S2/Supplementary_Figure_S2_087.tif]

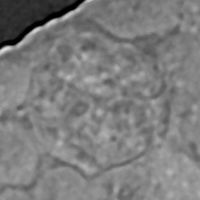

Supplement: Supplementary file 3 — Supplementary Information 3. [file 41598_2022_13394_MOESM3_ESM.zip › Supplementary Figure S2/Supplementary_Figure_S2_088.tif]

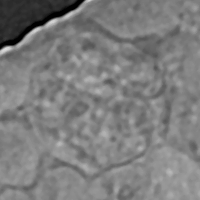

Supplement: Supplementary file 3 — Supplementary Information 3. [file 41598_2022_13394_MOESM3_ESM.zip › Supplementary Figure S2/Supplementary_Figure_S2_089.tif]

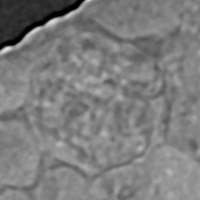

Supplement: Supplementary file 3 — Supplementary Information 3. [file 41598_2022_13394_MOESM3_ESM.zip › Supplementary Figure S2/Supplementary_Figure_S2_090.tif]

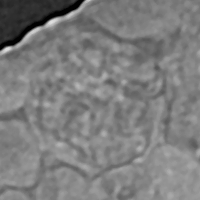

Supplement: Supplementary file 3 — Supplementary Information 3. [file 41598_2022_13394_MOESM3_ESM.zip › Supplementary Figure S2/Supplementary_Figure_S2_091.tif]

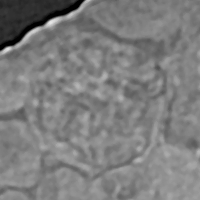

Supplement: Supplementary file 3 — Supplementary Information 3. [file 41598_2022_13394_MOESM3_ESM.zip › Supplementary Figure S2/Supplementary_Figure_S2_092.tif]

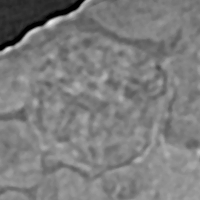

Supplement: Supplementary file 3 — Supplementary Information 3. [file 41598_2022_13394_MOESM3_ESM.zip › Supplementary Figure S2/Supplementary_Figure_S2_093.tif]

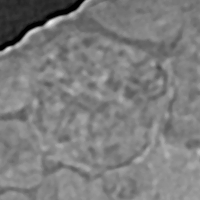

Supplement: Supplementary file 3 — Supplementary Information 3. [file 41598_2022_13394_MOESM3_ESM.zip › Supplementary Figure S2/Supplementary_Figure_S2_094.tif]

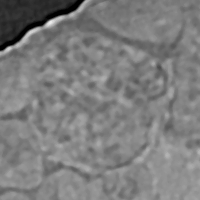

Supplement: Supplementary file 3 — Supplementary Information 3. [file 41598_2022_13394_MOESM3_ESM.zip › Supplementary Figure S2/Supplementary_Figure_S2_095.tif]

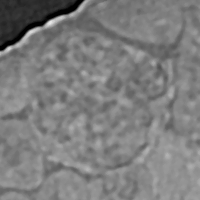

Supplement: Supplementary file 3 — Supplementary Information 3. [file 41598_2022_13394_MOESM3_ESM.zip › Supplementary Figure S2/Supplementary_Figure_S2_096.tif]

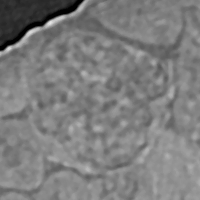

Supplement: Supplementary file 3 — Supplementary Information 3. [file 41598_2022_13394_MOESM3_ESM.zip › Supplementary Figure S2/Supplementary_Figure_S2_097.tif]

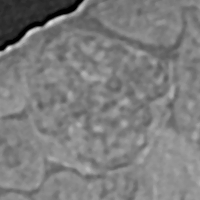

Supplement: Supplementary file 3 — Supplementary Information 3. [file 41598_2022_13394_MOESM3_ESM.zip › Supplementary Figure S2/Supplementary_Figure_S2_098.tif]
