# Supplementary figures and images for: Nondestructive cellular-level 3D observation of mouse kidney using laboratory-based X-ray microscopy with paraffin-mediated contrast enhancement (part 3 of 9)
Source: Sci Rep. 2022 Jun 8;12:9436. doi: 10.1038/s41598-022-13394-9 (PMC9177607; doi:10.1038/s41598-022-13394-9)

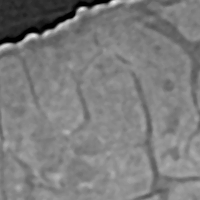

Supplement: Supplementary file 3 — Supplementary Information 3. [file 41598_2022_13394_MOESM3_ESM.zip › Supplementary Figure S2/Supplementary_Figure_S2_199.tif]

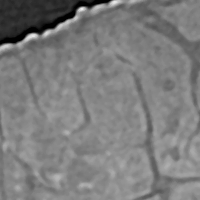

Supplement: Supplementary file 3 — Supplementary Information 3. [file 41598_2022_13394_MOESM3_ESM.zip › Supplementary Figure S2/Supplementary_Figure_S2_200.tif]

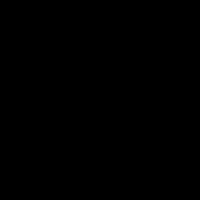

Supplement: Supplementary file 4 — Supplementary Information 4. [file 41598_2022_13394_MOESM4_ESM.zip › Supplementary Figure S3/Supplementary_Figure_S3_001.tif]

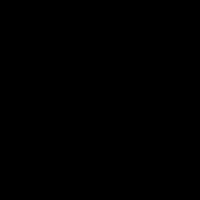

Supplement: Supplementary file 4 — Supplementary Information 4. [file 41598_2022_13394_MOESM4_ESM.zip › Supplementary Figure S3/Supplementary_Figure_S3_002.tif]

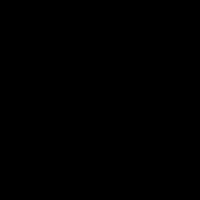

Supplement: Supplementary file 4 — Supplementary Information 4. [file 41598_2022_13394_MOESM4_ESM.zip › Supplementary Figure S3/Supplementary_Figure_S3_003.tif]

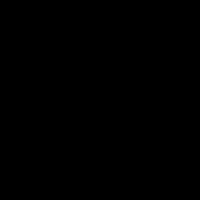

Supplement: Supplementary file 4 — Supplementary Information 4. [file 41598_2022_13394_MOESM4_ESM.zip › Supplementary Figure S3/Supplementary_Figure_S3_004.tif]

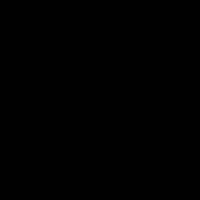

Supplement: Supplementary file 4 — Supplementary Information 4. [file 41598_2022_13394_MOESM4_ESM.zip › Supplementary Figure S3/Supplementary_Figure_S3_005.tif]

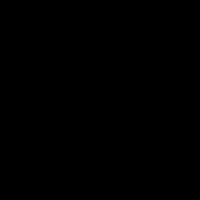

Supplement: Supplementary file 4 — Supplementary Information 4. [file 41598_2022_13394_MOESM4_ESM.zip › Supplementary Figure S3/Supplementary_Figure_S3_006.tif]

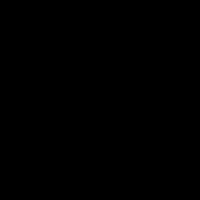

Supplement: Supplementary file 4 — Supplementary Information 4. [file 41598_2022_13394_MOESM4_ESM.zip › Supplementary Figure S3/Supplementary_Figure_S3_007.tif]

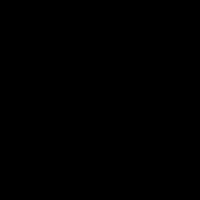

Supplement: Supplementary file 4 — Supplementary Information 4. [file 41598_2022_13394_MOESM4_ESM.zip › Supplementary Figure S3/Supplementary_Figure_S3_008.tif]

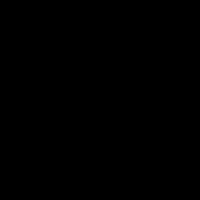

Supplement: Supplementary file 4 — Supplementary Information 4. [file 41598_2022_13394_MOESM4_ESM.zip › Supplementary Figure S3/Supplementary_Figure_S3_009.tif]

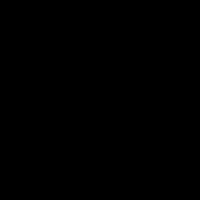

Supplement: Supplementary file 4 — Supplementary Information 4. [file 41598_2022_13394_MOESM4_ESM.zip › Supplementary Figure S3/Supplementary_Figure_S3_010.tif]

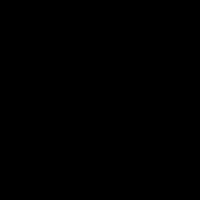

Supplement: Supplementary file 4 — Supplementary Information 4. [file 41598_2022_13394_MOESM4_ESM.zip › Supplementary Figure S3/Supplementary_Figure_S3_011.tif]

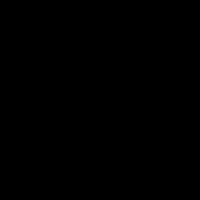

Supplement: Supplementary file 4 — Supplementary Information 4. [file 41598_2022_13394_MOESM4_ESM.zip › Supplementary Figure S3/Supplementary_Figure_S3_012.tif]

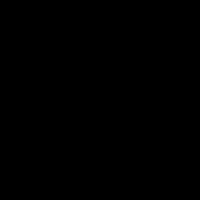

Supplement: Supplementary file 4 — Supplementary Information 4. [file 41598_2022_13394_MOESM4_ESM.zip › Supplementary Figure S3/Supplementary_Figure_S3_013.tif]

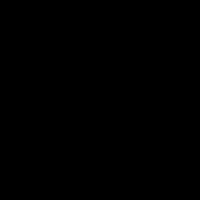

Supplement: Supplementary file 4 — Supplementary Information 4. [file 41598_2022_13394_MOESM4_ESM.zip › Supplementary Figure S3/Supplementary_Figure_S3_014.tif]

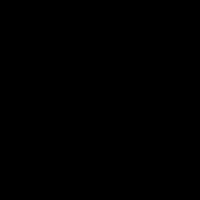

Supplement: Supplementary file 4 — Supplementary Information 4. [file 41598_2022_13394_MOESM4_ESM.zip › Supplementary Figure S3/Supplementary_Figure_S3_015.tif]

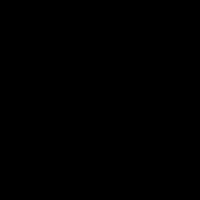

Supplement: Supplementary file 4 — Supplementary Information 4. [file 41598_2022_13394_MOESM4_ESM.zip › Supplementary Figure S3/Supplementary_Figure_S3_016.tif]

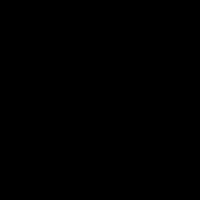

Supplement: Supplementary file 4 — Supplementary Information 4. [file 41598_2022_13394_MOESM4_ESM.zip › Supplementary Figure S3/Supplementary_Figure_S3_017.tif]

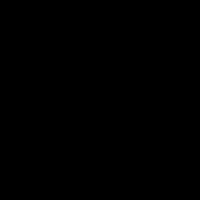

Supplement: Supplementary file 4 — Supplementary Information 4. [file 41598_2022_13394_MOESM4_ESM.zip › Supplementary Figure S3/Supplementary_Figure_S3_018.tif]

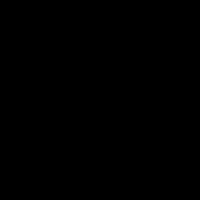

Supplement: Supplementary file 4 — Supplementary Information 4. [file 41598_2022_13394_MOESM4_ESM.zip › Supplementary Figure S3/Supplementary_Figure_S3_019.tif]

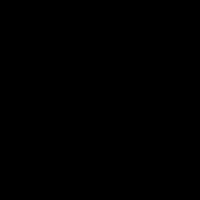

Supplement: Supplementary file 4 — Supplementary Information 4. [file 41598_2022_13394_MOESM4_ESM.zip › Supplementary Figure S3/Supplementary_Figure_S3_020.tif]

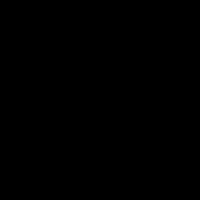

Supplement: Supplementary file 4 — Supplementary Information 4. [file 41598_2022_13394_MOESM4_ESM.zip › Supplementary Figure S3/Supplementary_Figure_S3_021.tif]

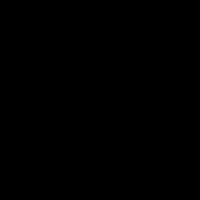

Supplement: Supplementary file 4 — Supplementary Information 4. [file 41598_2022_13394_MOESM4_ESM.zip › Supplementary Figure S3/Supplementary_Figure_S3_022.tif]

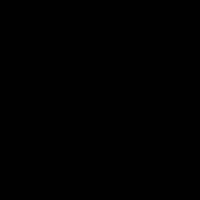

Supplement: Supplementary file 4 — Supplementary Information 4. [file 41598_2022_13394_MOESM4_ESM.zip › Supplementary Figure S3/Supplementary_Figure_S3_023.tif]

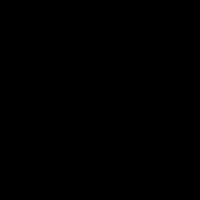

Supplement: Supplementary file 4 — Supplementary Information 4. [file 41598_2022_13394_MOESM4_ESM.zip › Supplementary Figure S3/Supplementary_Figure_S3_024.tif]

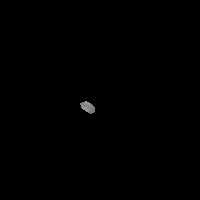

Supplement: Supplementary file 4 — Supplementary Information 4. [file 41598_2022_13394_MOESM4_ESM.zip › Supplementary Figure S3/Supplementary_Figure_S3_025.tif]

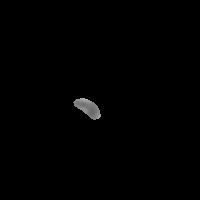

Supplement: Supplementary file 4 — Supplementary Information 4. [file 41598_2022_13394_MOESM4_ESM.zip › Supplementary Figure S3/Supplementary_Figure_S3_026.tif]

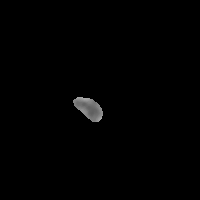

Supplement: Supplementary file 4 — Supplementary Information 4. [file 41598_2022_13394_MOESM4_ESM.zip › Supplementary Figure S3/Supplementary_Figure_S3_027.tif]

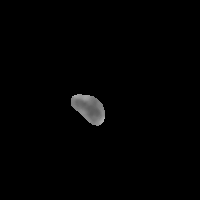

Supplement: Supplementary file 4 — Supplementary Information 4. [file 41598_2022_13394_MOESM4_ESM.zip › Supplementary Figure S3/Supplementary_Figure_S3_028.tif]

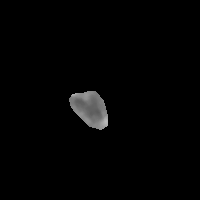

Supplement: Supplementary file 4 — Supplementary Information 4. [file 41598_2022_13394_MOESM4_ESM.zip › Supplementary Figure S3/Supplementary_Figure_S3_029.tif]

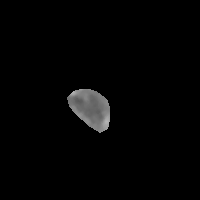

Supplement: Supplementary file 4 — Supplementary Information 4. [file 41598_2022_13394_MOESM4_ESM.zip › Supplementary Figure S3/Supplementary_Figure_S3_030.tif]

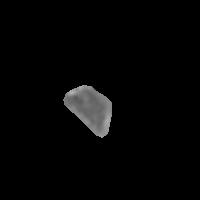

Supplement: Supplementary file 4 — Supplementary Information 4. [file 41598_2022_13394_MOESM4_ESM.zip › Supplementary Figure S3/Supplementary_Figure_S3_031.tif]

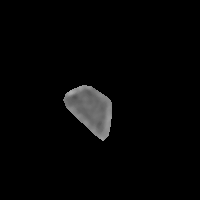

Supplement: Supplementary file 4 — Supplementary Information 4. [file 41598_2022_13394_MOESM4_ESM.zip › Supplementary Figure S3/Supplementary_Figure_S3_032.tif]

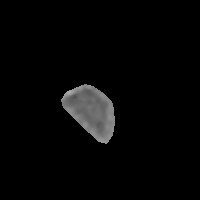

Supplement: Supplementary file 4 — Supplementary Information 4. [file 41598_2022_13394_MOESM4_ESM.zip › Supplementary Figure S3/Supplementary_Figure_S3_033.tif]

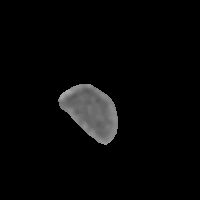

Supplement: Supplementary file 4 — Supplementary Information 4. [file 41598_2022_13394_MOESM4_ESM.zip › Supplementary Figure S3/Supplementary_Figure_S3_034.tif]

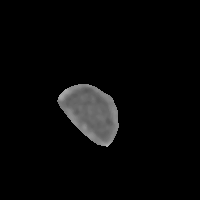

Supplement: Supplementary file 4 — Supplementary Information 4. [file 41598_2022_13394_MOESM4_ESM.zip › Supplementary Figure S3/Supplementary_Figure_S3_035.tif]

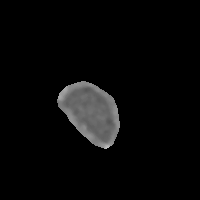

Supplement: Supplementary file 4 — Supplementary Information 4. [file 41598_2022_13394_MOESM4_ESM.zip › Supplementary Figure S3/Supplementary_Figure_S3_036.tif]

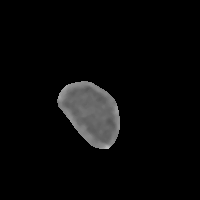

Supplement: Supplementary file 4 — Supplementary Information 4. [file 41598_2022_13394_MOESM4_ESM.zip › Supplementary Figure S3/Supplementary_Figure_S3_037.tif]

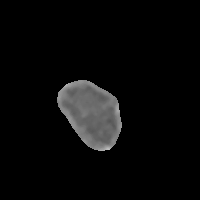

Supplement: Supplementary file 4 — Supplementary Information 4. [file 41598_2022_13394_MOESM4_ESM.zip › Supplementary Figure S3/Supplementary_Figure_S3_038.tif]

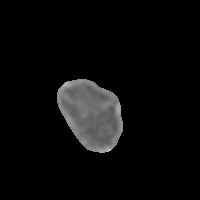

Supplement: Supplementary file 4 — Supplementary Information 4. [file 41598_2022_13394_MOESM4_ESM.zip › Supplementary Figure S3/Supplementary_Figure_S3_039.tif]

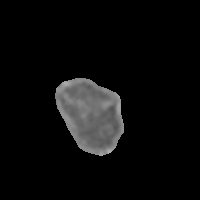

Supplement: Supplementary file 4 — Supplementary Information 4. [file 41598_2022_13394_MOESM4_ESM.zip › Supplementary Figure S3/Supplementary_Figure_S3_040.tif]

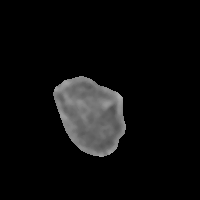

Supplement: Supplementary file 4 — Supplementary Information 4. [file 41598_2022_13394_MOESM4_ESM.zip › Supplementary Figure S3/Supplementary_Figure_S3_041.tif]

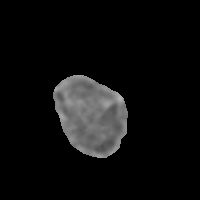

Supplement: Supplementary file 4 — Supplementary Information 4. [file 41598_2022_13394_MOESM4_ESM.zip › Supplementary Figure S3/Supplementary_Figure_S3_042.tif]

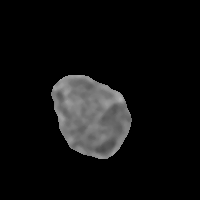

Supplement: Supplementary file 4 — Supplementary Information 4. [file 41598_2022_13394_MOESM4_ESM.zip › Supplementary Figure S3/Supplementary_Figure_S3_043.tif]

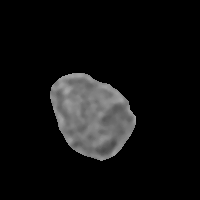

Supplement: Supplementary file 4 — Supplementary Information 4. [file 41598_2022_13394_MOESM4_ESM.zip › Supplementary Figure S3/Supplementary_Figure_S3_044.tif]

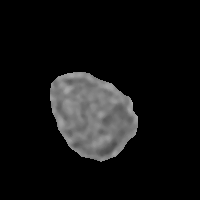

Supplement: Supplementary file 4 — Supplementary Information 4. [file 41598_2022_13394_MOESM4_ESM.zip › Supplementary Figure S3/Supplementary_Figure_S3_045.tif]

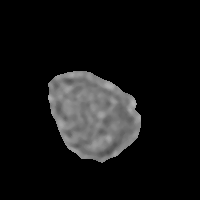

Supplement: Supplementary file 4 — Supplementary Information 4. [file 41598_2022_13394_MOESM4_ESM.zip › Supplementary Figure S3/Supplementary_Figure_S3_046.tif]

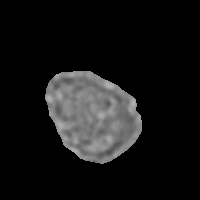

Supplement: Supplementary file 4 — Supplementary Information 4. [file 41598_2022_13394_MOESM4_ESM.zip › Supplementary Figure S3/Supplementary_Figure_S3_047.tif]

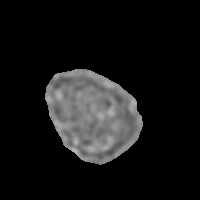

Supplement: Supplementary file 4 — Supplementary Information 4. [file 41598_2022_13394_MOESM4_ESM.zip › Supplementary Figure S3/Supplementary_Figure_S3_048.tif]

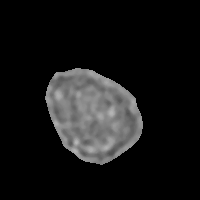

Supplement: Supplementary file 4 — Supplementary Information 4. [file 41598_2022_13394_MOESM4_ESM.zip › Supplementary Figure S3/Supplementary_Figure_S3_049.tif]

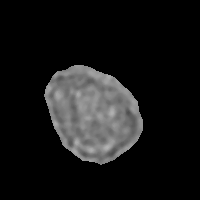

Supplement: Supplementary file 4 — Supplementary Information 4. [file 41598_2022_13394_MOESM4_ESM.zip › Supplementary Figure S3/Supplementary_Figure_S3_050.tif]

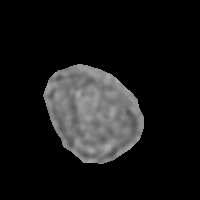

Supplement: Supplementary file 4 — Supplementary Information 4. [file 41598_2022_13394_MOESM4_ESM.zip › Supplementary Figure S3/Supplementary_Figure_S3_051.tif]

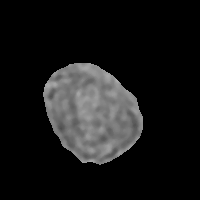

Supplement: Supplementary file 4 — Supplementary Information 4. [file 41598_2022_13394_MOESM4_ESM.zip › Supplementary Figure S3/Supplementary_Figure_S3_052.tif]

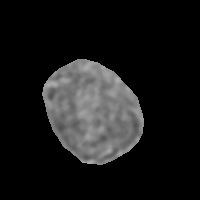

Supplement: Supplementary file 4 — Supplementary Information 4. [file 41598_2022_13394_MOESM4_ESM.zip › Supplementary Figure S3/Supplementary_Figure_S3_053.tif]

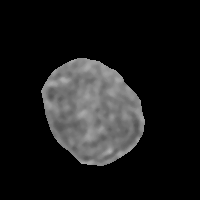

Supplement: Supplementary file 4 — Supplementary Information 4. [file 41598_2022_13394_MOESM4_ESM.zip › Supplementary Figure S3/Supplementary_Figure_S3_054.tif]

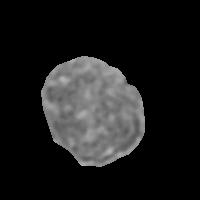

Supplement: Supplementary file 4 — Supplementary Information 4. [file 41598_2022_13394_MOESM4_ESM.zip › Supplementary Figure S3/Supplementary_Figure_S3_055.tif]

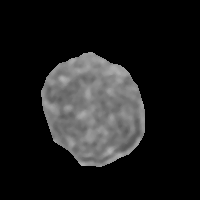

Supplement: Supplementary file 4 — Supplementary Information 4. [file 41598_2022_13394_MOESM4_ESM.zip › Supplementary Figure S3/Supplementary_Figure_S3_056.tif]

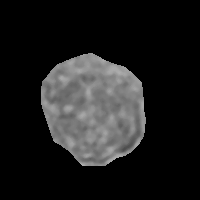

Supplement: Supplementary file 4 — Supplementary Information 4. [file 41598_2022_13394_MOESM4_ESM.zip › Supplementary Figure S3/Supplementary_Figure_S3_057.tif]

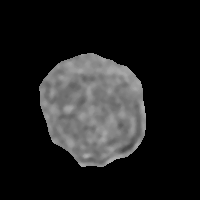

Supplement: Supplementary file 4 — Supplementary Information 4. [file 41598_2022_13394_MOESM4_ESM.zip › Supplementary Figure S3/Supplementary_Figure_S3_058.tif]

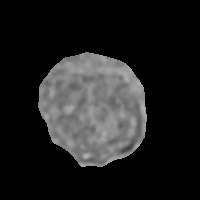

Supplement: Supplementary file 4 — Supplementary Information 4. [file 41598_2022_13394_MOESM4_ESM.zip › Supplementary Figure S3/Supplementary_Figure_S3_059.tif]

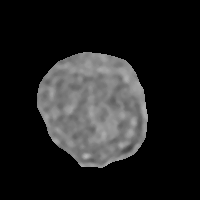

Supplement: Supplementary file 4 — Supplementary Information 4. [file 41598_2022_13394_MOESM4_ESM.zip › Supplementary Figure S3/Supplementary_Figure_S3_060.tif]

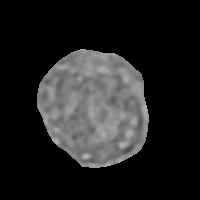

Supplement: Supplementary file 4 — Supplementary Information 4. [file 41598_2022_13394_MOESM4_ESM.zip › Supplementary Figure S3/Supplementary_Figure_S3_061.tif]

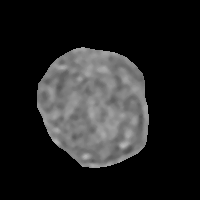

Supplement: Supplementary file 4 — Supplementary Information 4. [file 41598_2022_13394_MOESM4_ESM.zip › Supplementary Figure S3/Supplementary_Figure_S3_062.tif]

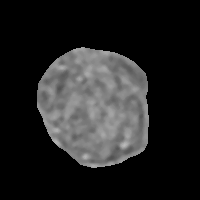

Supplement: Supplementary file 4 — Supplementary Information 4. [file 41598_2022_13394_MOESM4_ESM.zip › Supplementary Figure S3/Supplementary_Figure_S3_063.tif]

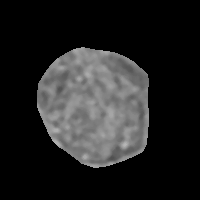

Supplement: Supplementary file 4 — Supplementary Information 4. [file 41598_2022_13394_MOESM4_ESM.zip › Supplementary Figure S3/Supplementary_Figure_S3_064.tif]

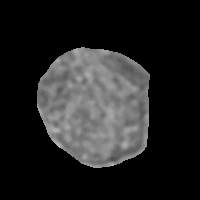

Supplement: Supplementary file 4 — Supplementary Information 4. [file 41598_2022_13394_MOESM4_ESM.zip › Supplementary Figure S3/Supplementary_Figure_S3_065.tif]

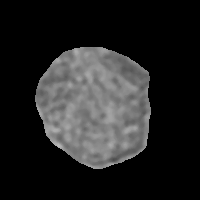

Supplement: Supplementary file 4 — Supplementary Information 4. [file 41598_2022_13394_MOESM4_ESM.zip › Supplementary Figure S3/Supplementary_Figure_S3_066.tif]

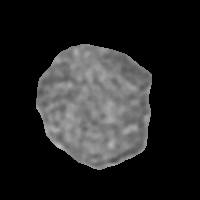

Supplement: Supplementary file 4 — Supplementary Information 4. [file 41598_2022_13394_MOESM4_ESM.zip › Supplementary Figure S3/Supplementary_Figure_S3_067.tif]

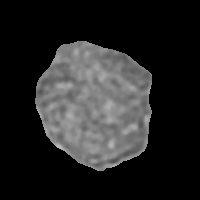

Supplement: Supplementary file 4 — Supplementary Information 4. [file 41598_2022_13394_MOESM4_ESM.zip › Supplementary Figure S3/Supplementary_Figure_S3_068.tif]

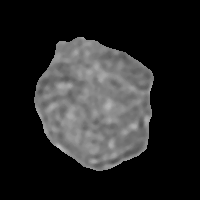

Supplement: Supplementary file 4 — Supplementary Information 4. [file 41598_2022_13394_MOESM4_ESM.zip › Supplementary Figure S3/Supplementary_Figure_S3_069.tif]

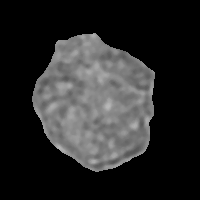

Supplement: Supplementary file 4 — Supplementary Information 4. [file 41598_2022_13394_MOESM4_ESM.zip › Supplementary Figure S3/Supplementary_Figure_S3_070.tif]

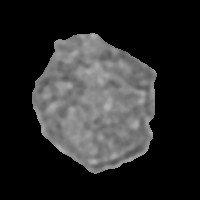

Supplement: Supplementary file 4 — Supplementary Information 4. [file 41598_2022_13394_MOESM4_ESM.zip › Supplementary Figure S3/Supplementary_Figure_S3_071.tif]

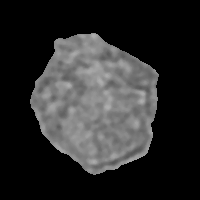

Supplement: Supplementary file 4 — Supplementary Information 4. [file 41598_2022_13394_MOESM4_ESM.zip › Supplementary Figure S3/Supplementary_Figure_S3_072.tif]

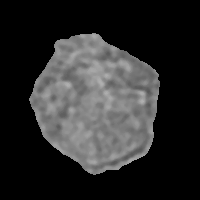

Supplement: Supplementary file 4 — Supplementary Information 4. [file 41598_2022_13394_MOESM4_ESM.zip › Supplementary Figure S3/Supplementary_Figure_S3_073.tif]

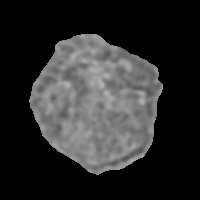

Supplement: Supplementary file 4 — Supplementary Information 4. [file 41598_2022_13394_MOESM4_ESM.zip › Supplementary Figure S3/Supplementary_Figure_S3_074.tif]

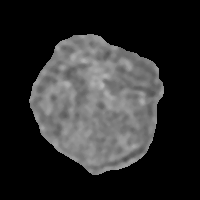

Supplement: Supplementary file 4 — Supplementary Information 4. [file 41598_2022_13394_MOESM4_ESM.zip › Supplementary Figure S3/Supplementary_Figure_S3_075.tif]

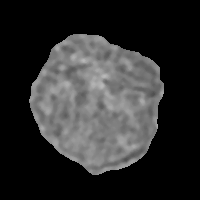

Supplement: Supplementary file 4 — Supplementary Information 4. [file 41598_2022_13394_MOESM4_ESM.zip › Supplementary Figure S3/Supplementary_Figure_S3_076.tif]

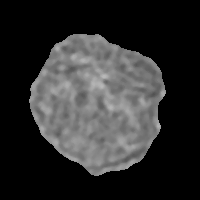

Supplement: Supplementary file 4 — Supplementary Information 4. [file 41598_2022_13394_MOESM4_ESM.zip › Supplementary Figure S3/Supplementary_Figure_S3_077.tif]

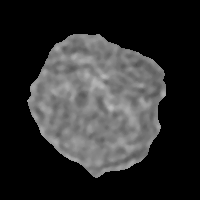

Supplement: Supplementary file 4 — Supplementary Information 4. [file 41598_2022_13394_MOESM4_ESM.zip › Supplementary Figure S3/Supplementary_Figure_S3_078.tif]

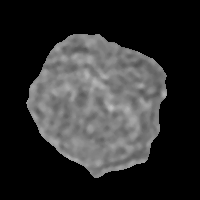

Supplement: Supplementary file 4 — Supplementary Information 4. [file 41598_2022_13394_MOESM4_ESM.zip › Supplementary Figure S3/Supplementary_Figure_S3_079.tif]

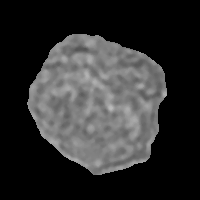

Supplement: Supplementary file 4 — Supplementary Information 4. [file 41598_2022_13394_MOESM4_ESM.zip › Supplementary Figure S3/Supplementary_Figure_S3_080.tif]

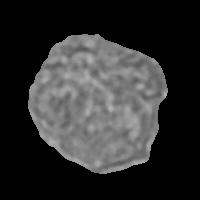

Supplement: Supplementary file 4 — Supplementary Information 4. [file 41598_2022_13394_MOESM4_ESM.zip › Supplementary Figure S3/Supplementary_Figure_S3_081.tif]

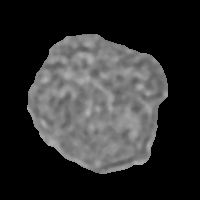

Supplement: Supplementary file 4 — Supplementary Information 4. [file 41598_2022_13394_MOESM4_ESM.zip › Supplementary Figure S3/Supplementary_Figure_S3_082.tif]

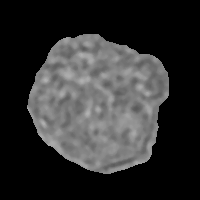

Supplement: Supplementary file 4 — Supplementary Information 4. [file 41598_2022_13394_MOESM4_ESM.zip › Supplementary Figure S3/Supplementary_Figure_S3_083.tif]

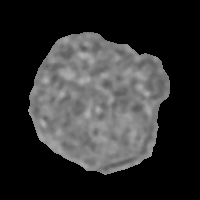

Supplement: Supplementary file 4 — Supplementary Information 4. [file 41598_2022_13394_MOESM4_ESM.zip › Supplementary Figure S3/Supplementary_Figure_S3_084.tif]

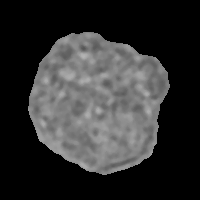

Supplement: Supplementary file 4 — Supplementary Information 4. [file 41598_2022_13394_MOESM4_ESM.zip › Supplementary Figure S3/Supplementary_Figure_S3_085.tif]

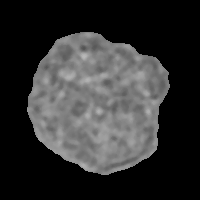

Supplement: Supplementary file 4 — Supplementary Information 4. [file 41598_2022_13394_MOESM4_ESM.zip › Supplementary Figure S3/Supplementary_Figure_S3_086.tif]

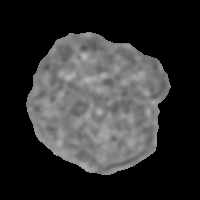

Supplement: Supplementary file 4 — Supplementary Information 4. [file 41598_2022_13394_MOESM4_ESM.zip › Supplementary Figure S3/Supplementary_Figure_S3_087.tif]

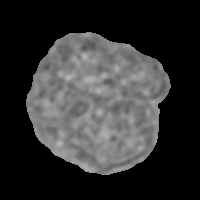

Supplement: Supplementary file 4 — Supplementary Information 4. [file 41598_2022_13394_MOESM4_ESM.zip › Supplementary Figure S3/Supplementary_Figure_S3_088.tif]

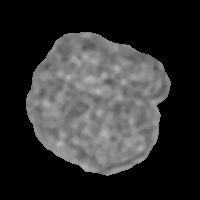

Supplement: Supplementary file 4 — Supplementary Information 4. [file 41598_2022_13394_MOESM4_ESM.zip › Supplementary Figure S3/Supplementary_Figure_S3_089.tif]

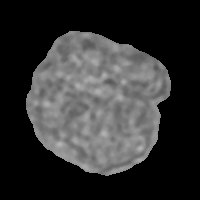

Supplement: Supplementary file 4 — Supplementary Information 4. [file 41598_2022_13394_MOESM4_ESM.zip › Supplementary Figure S3/Supplementary_Figure_S3_090.tif]

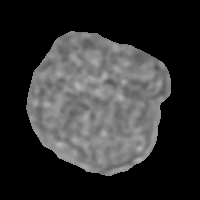

Supplement: Supplementary file 4 — Supplementary Information 4. [file 41598_2022_13394_MOESM4_ESM.zip › Supplementary Figure S3/Supplementary_Figure_S3_091.tif]

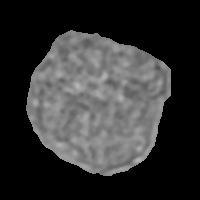

Supplement: Supplementary file 4 — Supplementary Information 4. [file 41598_2022_13394_MOESM4_ESM.zip › Supplementary Figure S3/Supplementary_Figure_S3_092.tif]

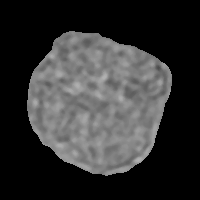

Supplement: Supplementary file 4 — Supplementary Information 4. [file 41598_2022_13394_MOESM4_ESM.zip › Supplementary Figure S3/Supplementary_Figure_S3_093.tif]

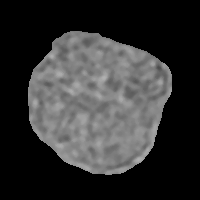

Supplement: Supplementary file 4 — Supplementary Information 4. [file 41598_2022_13394_MOESM4_ESM.zip › Supplementary Figure S3/Supplementary_Figure_S3_094.tif]

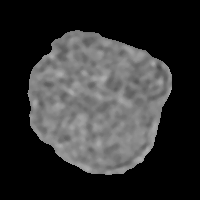

Supplement: Supplementary file 4 — Supplementary Information 4. [file 41598_2022_13394_MOESM4_ESM.zip › Supplementary Figure S3/Supplementary_Figure_S3_095.tif]

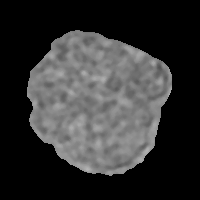

Supplement: Supplementary file 4 — Supplementary Information 4. [file 41598_2022_13394_MOESM4_ESM.zip › Supplementary Figure S3/Supplementary_Figure_S3_096.tif]

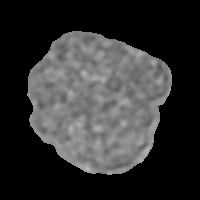

Supplement: Supplementary file 4 — Supplementary Information 4. [file 41598_2022_13394_MOESM4_ESM.zip › Supplementary Figure S3/Supplementary_Figure_S3_097.tif]

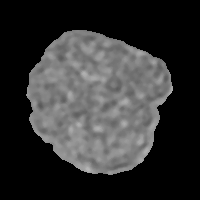

Supplement: Supplementary file 4 — Supplementary Information 4. [file 41598_2022_13394_MOESM4_ESM.zip › Supplementary Figure S3/Supplementary_Figure_S3_098.tif]
