# Supplementary figures and images for: Nondestructive cellular-level 3D observation of mouse kidney using laboratory-based X-ray microscopy with paraffin-mediated contrast enhancement (part 2 of 9)
Source: Sci Rep. 2022 Jun 8;12:9436. doi: 10.1038/s41598-022-13394-9 (PMC9177607; doi:10.1038/s41598-022-13394-9)

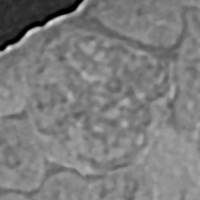

Supplement: Supplementary file 3 — Supplementary Information 3. [file 41598_2022_13394_MOESM3_ESM.zip › Supplementary Figure S2/Supplementary_Figure_S2_099.tif]

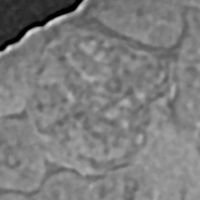

Supplement: Supplementary file 3 — Supplementary Information 3. [file 41598_2022_13394_MOESM3_ESM.zip › Supplementary Figure S2/Supplementary_Figure_S2_100.tif]

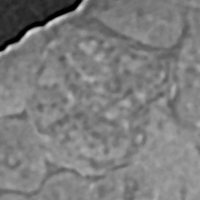

Supplement: Supplementary file 3 — Supplementary Information 3. [file 41598_2022_13394_MOESM3_ESM.zip › Supplementary Figure S2/Supplementary_Figure_S2_101.tif]

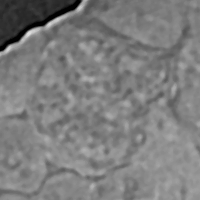

Supplement: Supplementary file 3 — Supplementary Information 3. [file 41598_2022_13394_MOESM3_ESM.zip › Supplementary Figure S2/Supplementary_Figure_S2_102.tif]

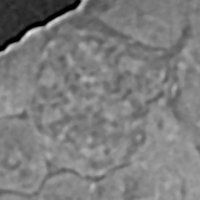

Supplement: Supplementary file 3 — Supplementary Information 3. [file 41598_2022_13394_MOESM3_ESM.zip › Supplementary Figure S2/Supplementary_Figure_S2_103.tif]

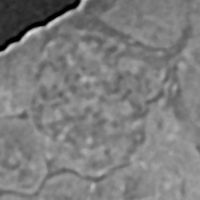

Supplement: Supplementary file 3 — Supplementary Information 3. [file 41598_2022_13394_MOESM3_ESM.zip › Supplementary Figure S2/Supplementary_Figure_S2_104.tif]

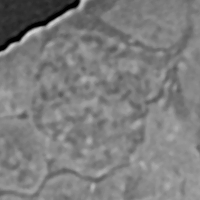

Supplement: Supplementary file 3 — Supplementary Information 3. [file 41598_2022_13394_MOESM3_ESM.zip › Supplementary Figure S2/Supplementary_Figure_S2_105.tif]

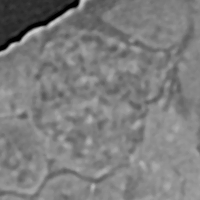

Supplement: Supplementary file 3 — Supplementary Information 3. [file 41598_2022_13394_MOESM3_ESM.zip › Supplementary Figure S2/Supplementary_Figure_S2_106.tif]

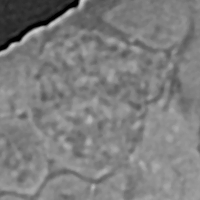

Supplement: Supplementary file 3 — Supplementary Information 3. [file 41598_2022_13394_MOESM3_ESM.zip › Supplementary Figure S2/Supplementary_Figure_S2_107.tif]

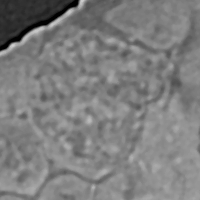

Supplement: Supplementary file 3 — Supplementary Information 3. [file 41598_2022_13394_MOESM3_ESM.zip › Supplementary Figure S2/Supplementary_Figure_S2_108.tif]

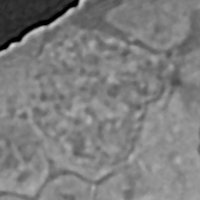

Supplement: Supplementary file 3 — Supplementary Information 3. [file 41598_2022_13394_MOESM3_ESM.zip › Supplementary Figure S2/Supplementary_Figure_S2_109.tif]

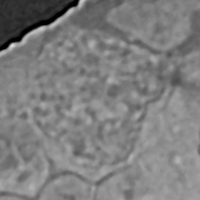

Supplement: Supplementary file 3 — Supplementary Information 3. [file 41598_2022_13394_MOESM3_ESM.zip › Supplementary Figure S2/Supplementary_Figure_S2_110.tif]

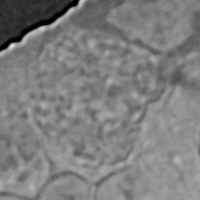

Supplement: Supplementary file 3 — Supplementary Information 3. [file 41598_2022_13394_MOESM3_ESM.zip › Supplementary Figure S2/Supplementary_Figure_S2_111.tif]

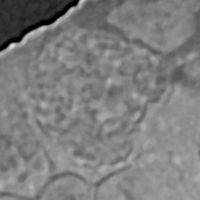

Supplement: Supplementary file 3 — Supplementary Information 3. [file 41598_2022_13394_MOESM3_ESM.zip › Supplementary Figure S2/Supplementary_Figure_S2_112.tif]

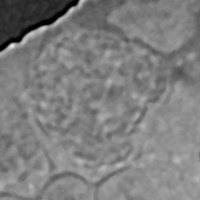

Supplement: Supplementary file 3 — Supplementary Information 3. [file 41598_2022_13394_MOESM3_ESM.zip › Supplementary Figure S2/Supplementary_Figure_S2_113.tif]

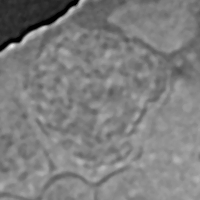

Supplement: Supplementary file 3 — Supplementary Information 3. [file 41598_2022_13394_MOESM3_ESM.zip › Supplementary Figure S2/Supplementary_Figure_S2_114.tif]

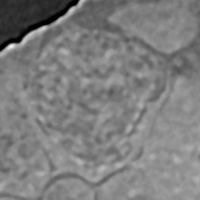

Supplement: Supplementary file 3 — Supplementary Information 3. [file 41598_2022_13394_MOESM3_ESM.zip › Supplementary Figure S2/Supplementary_Figure_S2_115.tif]

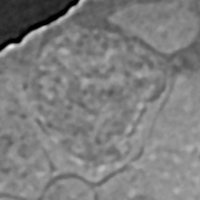

Supplement: Supplementary file 3 — Supplementary Information 3. [file 41598_2022_13394_MOESM3_ESM.zip › Supplementary Figure S2/Supplementary_Figure_S2_116.tif]

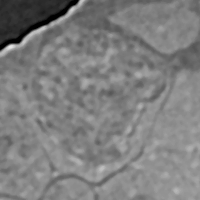

Supplement: Supplementary file 3 — Supplementary Information 3. [file 41598_2022_13394_MOESM3_ESM.zip › Supplementary Figure S2/Supplementary_Figure_S2_117.tif]

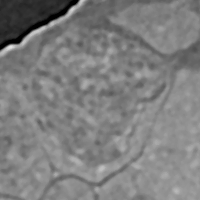

Supplement: Supplementary file 3 — Supplementary Information 3. [file 41598_2022_13394_MOESM3_ESM.zip › Supplementary Figure S2/Supplementary_Figure_S2_118.tif]

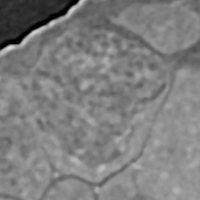

Supplement: Supplementary file 3 — Supplementary Information 3. [file 41598_2022_13394_MOESM3_ESM.zip › Supplementary Figure S2/Supplementary_Figure_S2_119.tif]

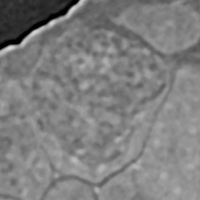

Supplement: Supplementary file 3 — Supplementary Information 3. [file 41598_2022_13394_MOESM3_ESM.zip › Supplementary Figure S2/Supplementary_Figure_S2_120.tif]

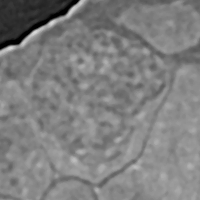

Supplement: Supplementary file 3 — Supplementary Information 3. [file 41598_2022_13394_MOESM3_ESM.zip › Supplementary Figure S2/Supplementary_Figure_S2_121.tif]

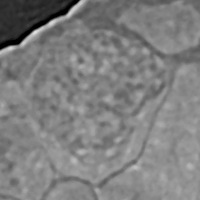

Supplement: Supplementary file 3 — Supplementary Information 3. [file 41598_2022_13394_MOESM3_ESM.zip › Supplementary Figure S2/Supplementary_Figure_S2_122.tif]

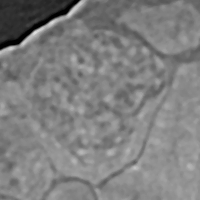

Supplement: Supplementary file 3 — Supplementary Information 3. [file 41598_2022_13394_MOESM3_ESM.zip › Supplementary Figure S2/Supplementary_Figure_S2_123.tif]

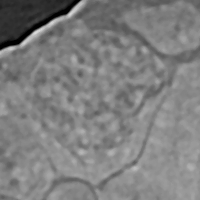

Supplement: Supplementary file 3 — Supplementary Information 3. [file 41598_2022_13394_MOESM3_ESM.zip › Supplementary Figure S2/Supplementary_Figure_S2_124.tif]

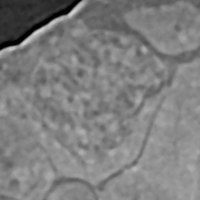

Supplement: Supplementary file 3 — Supplementary Information 3. [file 41598_2022_13394_MOESM3_ESM.zip › Supplementary Figure S2/Supplementary_Figure_S2_125.tif]

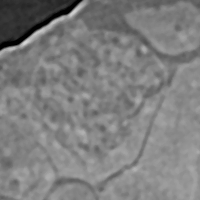

Supplement: Supplementary file 3 — Supplementary Information 3. [file 41598_2022_13394_MOESM3_ESM.zip › Supplementary Figure S2/Supplementary_Figure_S2_126.tif]

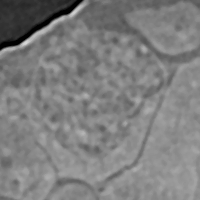

Supplement: Supplementary file 3 — Supplementary Information 3. [file 41598_2022_13394_MOESM3_ESM.zip › Supplementary Figure S2/Supplementary_Figure_S2_127.tif]

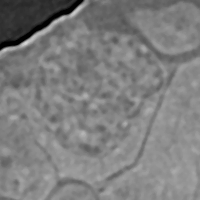

Supplement: Supplementary file 3 — Supplementary Information 3. [file 41598_2022_13394_MOESM3_ESM.zip › Supplementary Figure S2/Supplementary_Figure_S2_128.tif]

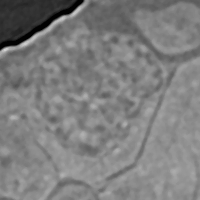

Supplement: Supplementary file 3 — Supplementary Information 3. [file 41598_2022_13394_MOESM3_ESM.zip › Supplementary Figure S2/Supplementary_Figure_S2_129.tif]

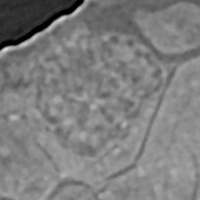

Supplement: Supplementary file 3 — Supplementary Information 3. [file 41598_2022_13394_MOESM3_ESM.zip › Supplementary Figure S2/Supplementary_Figure_S2_130.tif]

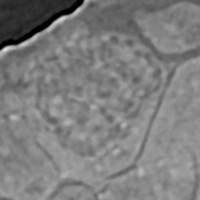

Supplement: Supplementary file 3 — Supplementary Information 3. [file 41598_2022_13394_MOESM3_ESM.zip › Supplementary Figure S2/Supplementary_Figure_S2_131.tif]

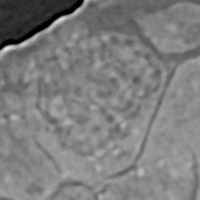

Supplement: Supplementary file 3 — Supplementary Information 3. [file 41598_2022_13394_MOESM3_ESM.zip › Supplementary Figure S2/Supplementary_Figure_S2_132.tif]

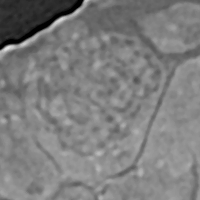

Supplement: Supplementary file 3 — Supplementary Information 3. [file 41598_2022_13394_MOESM3_ESM.zip › Supplementary Figure S2/Supplementary_Figure_S2_133.tif]

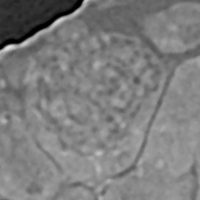

Supplement: Supplementary file 3 — Supplementary Information 3. [file 41598_2022_13394_MOESM3_ESM.zip › Supplementary Figure S2/Supplementary_Figure_S2_134.tif]

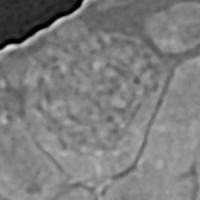

Supplement: Supplementary file 3 — Supplementary Information 3. [file 41598_2022_13394_MOESM3_ESM.zip › Supplementary Figure S2/Supplementary_Figure_S2_135.tif]

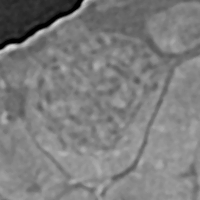

Supplement: Supplementary file 3 — Supplementary Information 3. [file 41598_2022_13394_MOESM3_ESM.zip › Supplementary Figure S2/Supplementary_Figure_S2_136.tif]

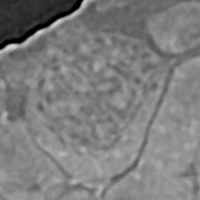

Supplement: Supplementary file 3 — Supplementary Information 3. [file 41598_2022_13394_MOESM3_ESM.zip › Supplementary Figure S2/Supplementary_Figure_S2_137.tif]

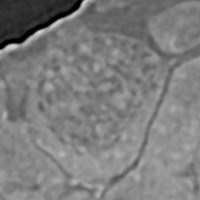

Supplement: Supplementary file 3 — Supplementary Information 3. [file 41598_2022_13394_MOESM3_ESM.zip › Supplementary Figure S2/Supplementary_Figure_S2_138.tif]

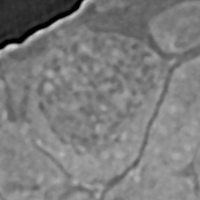

Supplement: Supplementary file 3 — Supplementary Information 3. [file 41598_2022_13394_MOESM3_ESM.zip › Supplementary Figure S2/Supplementary_Figure_S2_139.tif]

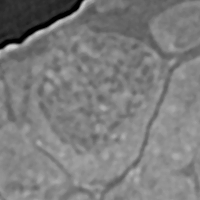

Supplement: Supplementary file 3 — Supplementary Information 3. [file 41598_2022_13394_MOESM3_ESM.zip › Supplementary Figure S2/Supplementary_Figure_S2_140.tif]

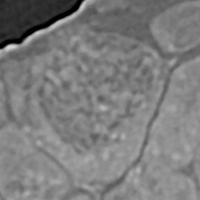

Supplement: Supplementary file 3 — Supplementary Information 3. [file 41598_2022_13394_MOESM3_ESM.zip › Supplementary Figure S2/Supplementary_Figure_S2_141.tif]

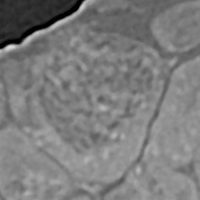

Supplement: Supplementary file 3 — Supplementary Information 3. [file 41598_2022_13394_MOESM3_ESM.zip › Supplementary Figure S2/Supplementary_Figure_S2_142.tif]

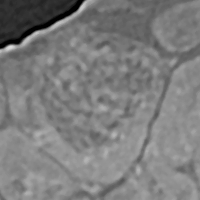

Supplement: Supplementary file 3 — Supplementary Information 3. [file 41598_2022_13394_MOESM3_ESM.zip › Supplementary Figure S2/Supplementary_Figure_S2_143.tif]

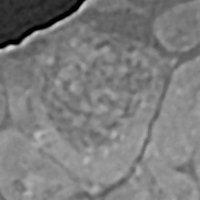

Supplement: Supplementary file 3 — Supplementary Information 3. [file 41598_2022_13394_MOESM3_ESM.zip › Supplementary Figure S2/Supplementary_Figure_S2_144.tif]

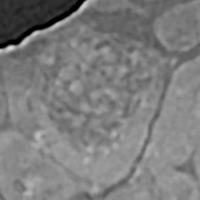

Supplement: Supplementary file 3 — Supplementary Information 3. [file 41598_2022_13394_MOESM3_ESM.zip › Supplementary Figure S2/Supplementary_Figure_S2_145.tif]

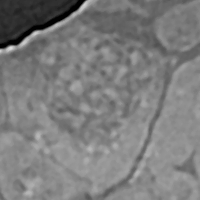

Supplement: Supplementary file 3 — Supplementary Information 3. [file 41598_2022_13394_MOESM3_ESM.zip › Supplementary Figure S2/Supplementary_Figure_S2_146.tif]

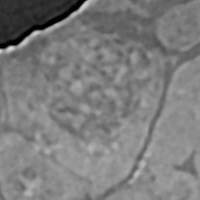

Supplement: Supplementary file 3 — Supplementary Information 3. [file 41598_2022_13394_MOESM3_ESM.zip › Supplementary Figure S2/Supplementary_Figure_S2_147.tif]

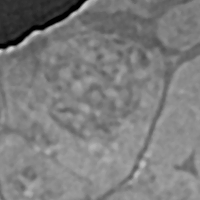

Supplement: Supplementary file 3 — Supplementary Information 3. [file 41598_2022_13394_MOESM3_ESM.zip › Supplementary Figure S2/Supplementary_Figure_S2_148.tif]

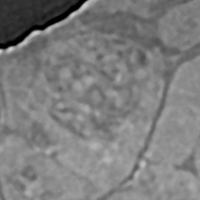

Supplement: Supplementary file 3 — Supplementary Information 3. [file 41598_2022_13394_MOESM3_ESM.zip › Supplementary Figure S2/Supplementary_Figure_S2_149.tif]

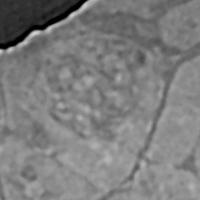

Supplement: Supplementary file 3 — Supplementary Information 3. [file 41598_2022_13394_MOESM3_ESM.zip › Supplementary Figure S2/Supplementary_Figure_S2_150.tif]

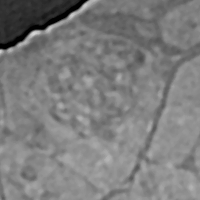

Supplement: Supplementary file 3 — Supplementary Information 3. [file 41598_2022_13394_MOESM3_ESM.zip › Supplementary Figure S2/Supplementary_Figure_S2_151.tif]

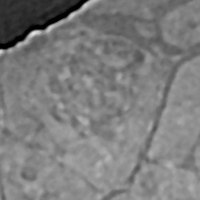

Supplement: Supplementary file 3 — Supplementary Information 3. [file 41598_2022_13394_MOESM3_ESM.zip › Supplementary Figure S2/Supplementary_Figure_S2_152.tif]

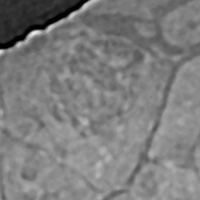

Supplement: Supplementary file 3 — Supplementary Information 3. [file 41598_2022_13394_MOESM3_ESM.zip › Supplementary Figure S2/Supplementary_Figure_S2_153.tif]

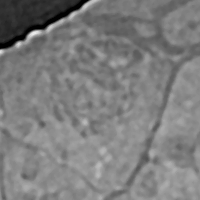

Supplement: Supplementary file 3 — Supplementary Information 3. [file 41598_2022_13394_MOESM3_ESM.zip › Supplementary Figure S2/Supplementary_Figure_S2_154.tif]

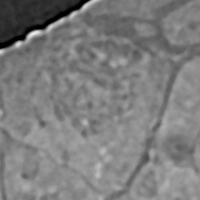

Supplement: Supplementary file 3 — Supplementary Information 3. [file 41598_2022_13394_MOESM3_ESM.zip › Supplementary Figure S2/Supplementary_Figure_S2_155.tif]

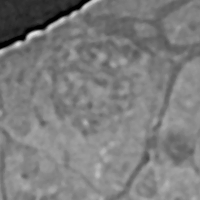

Supplement: Supplementary file 3 — Supplementary Information 3. [file 41598_2022_13394_MOESM3_ESM.zip › Supplementary Figure S2/Supplementary_Figure_S2_156.tif]

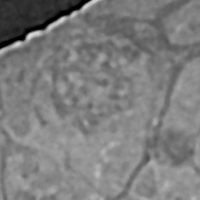

Supplement: Supplementary file 3 — Supplementary Information 3. [file 41598_2022_13394_MOESM3_ESM.zip › Supplementary Figure S2/Supplementary_Figure_S2_157.tif]

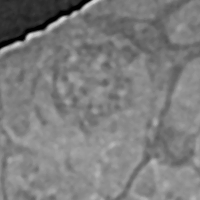

Supplement: Supplementary file 3 — Supplementary Information 3. [file 41598_2022_13394_MOESM3_ESM.zip › Supplementary Figure S2/Supplementary_Figure_S2_158.tif]

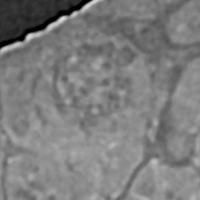

Supplement: Supplementary file 3 — Supplementary Information 3. [file 41598_2022_13394_MOESM3_ESM.zip › Supplementary Figure S2/Supplementary_Figure_S2_159.tif]

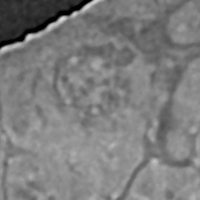

Supplement: Supplementary file 3 — Supplementary Information 3. [file 41598_2022_13394_MOESM3_ESM.zip › Supplementary Figure S2/Supplementary_Figure_S2_160.tif]

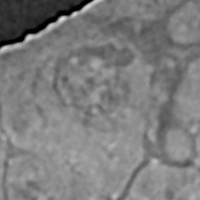

Supplement: Supplementary file 3 — Supplementary Information 3. [file 41598_2022_13394_MOESM3_ESM.zip › Supplementary Figure S2/Supplementary_Figure_S2_161.tif]

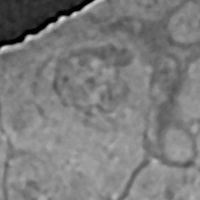

Supplement: Supplementary file 3 — Supplementary Information 3. [file 41598_2022_13394_MOESM3_ESM.zip › Supplementary Figure S2/Supplementary_Figure_S2_162.tif]

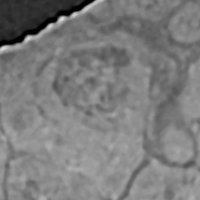

Supplement: Supplementary file 3 — Supplementary Information 3. [file 41598_2022_13394_MOESM3_ESM.zip › Supplementary Figure S2/Supplementary_Figure_S2_163.tif]

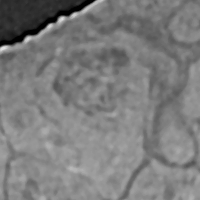

Supplement: Supplementary file 3 — Supplementary Information 3. [file 41598_2022_13394_MOESM3_ESM.zip › Supplementary Figure S2/Supplementary_Figure_S2_164.tif]

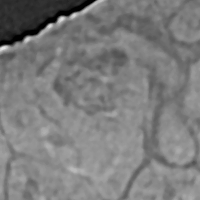

Supplement: Supplementary file 3 — Supplementary Information 3. [file 41598_2022_13394_MOESM3_ESM.zip › Supplementary Figure S2/Supplementary_Figure_S2_165.tif]

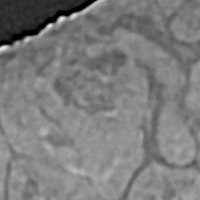

Supplement: Supplementary file 3 — Supplementary Information 3. [file 41598_2022_13394_MOESM3_ESM.zip › Supplementary Figure S2/Supplementary_Figure_S2_166.tif]

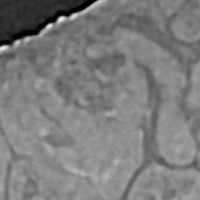

Supplement: Supplementary file 3 — Supplementary Information 3. [file 41598_2022_13394_MOESM3_ESM.zip › Supplementary Figure S2/Supplementary_Figure_S2_167.tif]

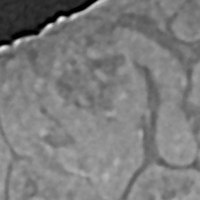

Supplement: Supplementary file 3 — Supplementary Information 3. [file 41598_2022_13394_MOESM3_ESM.zip › Supplementary Figure S2/Supplementary_Figure_S2_168.tif]

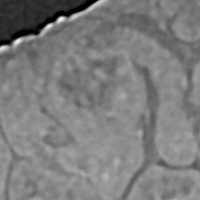

Supplement: Supplementary file 3 — Supplementary Information 3. [file 41598_2022_13394_MOESM3_ESM.zip › Supplementary Figure S2/Supplementary_Figure_S2_169.tif]

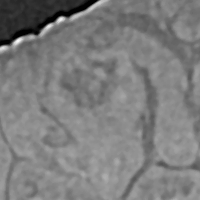

Supplement: Supplementary file 3 — Supplementary Information 3. [file 41598_2022_13394_MOESM3_ESM.zip › Supplementary Figure S2/Supplementary_Figure_S2_170.tif]

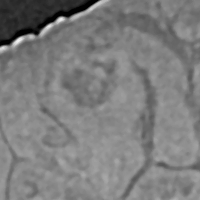

Supplement: Supplementary file 3 — Supplementary Information 3. [file 41598_2022_13394_MOESM3_ESM.zip › Supplementary Figure S2/Supplementary_Figure_S2_171.tif]

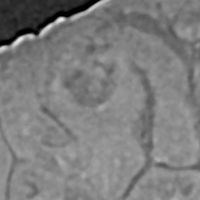

Supplement: Supplementary file 3 — Supplementary Information 3. [file 41598_2022_13394_MOESM3_ESM.zip › Supplementary Figure S2/Supplementary_Figure_S2_172.tif]

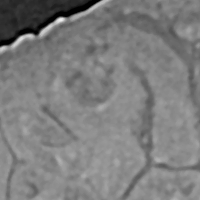

Supplement: Supplementary file 3 — Supplementary Information 3. [file 41598_2022_13394_MOESM3_ESM.zip › Supplementary Figure S2/Supplementary_Figure_S2_173.tif]

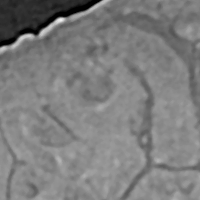

Supplement: Supplementary file 3 — Supplementary Information 3. [file 41598_2022_13394_MOESM3_ESM.zip › Supplementary Figure S2/Supplementary_Figure_S2_174.tif]

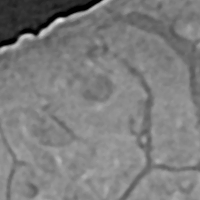

Supplement: Supplementary file 3 — Supplementary Information 3. [file 41598_2022_13394_MOESM3_ESM.zip › Supplementary Figure S2/Supplementary_Figure_S2_175.tif]

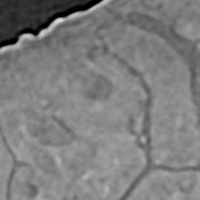

Supplement: Supplementary file 3 — Supplementary Information 3. [file 41598_2022_13394_MOESM3_ESM.zip › Supplementary Figure S2/Supplementary_Figure_S2_176.tif]

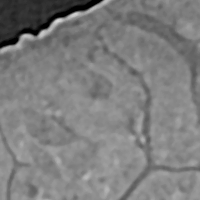

Supplement: Supplementary file 3 — Supplementary Information 3. [file 41598_2022_13394_MOESM3_ESM.zip › Supplementary Figure S2/Supplementary_Figure_S2_177.tif]

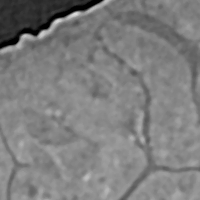

Supplement: Supplementary file 3 — Supplementary Information 3. [file 41598_2022_13394_MOESM3_ESM.zip › Supplementary Figure S2/Supplementary_Figure_S2_178.tif]

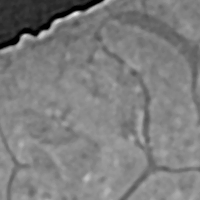

Supplement: Supplementary file 3 — Supplementary Information 3. [file 41598_2022_13394_MOESM3_ESM.zip › Supplementary Figure S2/Supplementary_Figure_S2_179.tif]

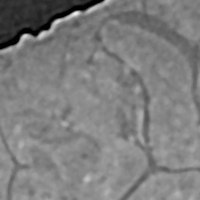

Supplement: Supplementary file 3 — Supplementary Information 3. [file 41598_2022_13394_MOESM3_ESM.zip › Supplementary Figure S2/Supplementary_Figure_S2_180.tif]

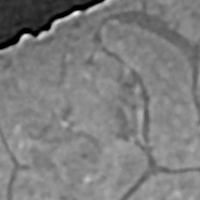

Supplement: Supplementary file 3 — Supplementary Information 3. [file 41598_2022_13394_MOESM3_ESM.zip › Supplementary Figure S2/Supplementary_Figure_S2_181.tif]

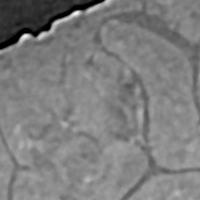

Supplement: Supplementary file 3 — Supplementary Information 3. [file 41598_2022_13394_MOESM3_ESM.zip › Supplementary Figure S2/Supplementary_Figure_S2_182.tif]

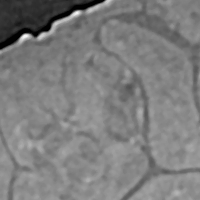

Supplement: Supplementary file 3 — Supplementary Information 3. [file 41598_2022_13394_MOESM3_ESM.zip › Supplementary Figure S2/Supplementary_Figure_S2_183.tif]

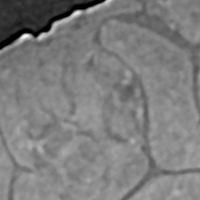

Supplement: Supplementary file 3 — Supplementary Information 3. [file 41598_2022_13394_MOESM3_ESM.zip › Supplementary Figure S2/Supplementary_Figure_S2_184.tif]

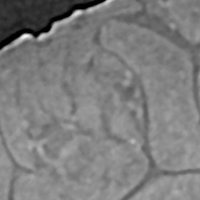

Supplement: Supplementary file 3 — Supplementary Information 3. [file 41598_2022_13394_MOESM3_ESM.zip › Supplementary Figure S2/Supplementary_Figure_S2_185.tif]

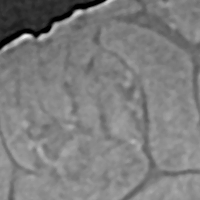

Supplement: Supplementary file 3 — Supplementary Information 3. [file 41598_2022_13394_MOESM3_ESM.zip › Supplementary Figure S2/Supplementary_Figure_S2_186.tif]

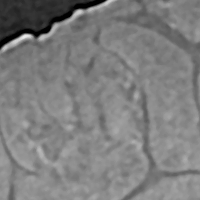

Supplement: Supplementary file 3 — Supplementary Information 3. [file 41598_2022_13394_MOESM3_ESM.zip › Supplementary Figure S2/Supplementary_Figure_S2_187.tif]

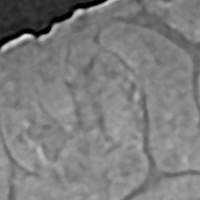

Supplement: Supplementary file 3 — Supplementary Information 3. [file 41598_2022_13394_MOESM3_ESM.zip › Supplementary Figure S2/Supplementary_Figure_S2_188.tif]

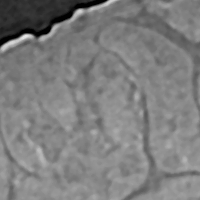

Supplement: Supplementary file 3 — Supplementary Information 3. [file 41598_2022_13394_MOESM3_ESM.zip › Supplementary Figure S2/Supplementary_Figure_S2_189.tif]

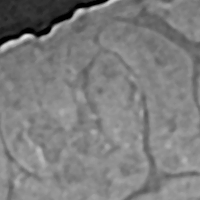

Supplement: Supplementary file 3 — Supplementary Information 3. [file 41598_2022_13394_MOESM3_ESM.zip › Supplementary Figure S2/Supplementary_Figure_S2_190.tif]

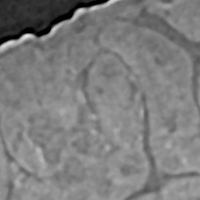

Supplement: Supplementary file 3 — Supplementary Information 3. [file 41598_2022_13394_MOESM3_ESM.zip › Supplementary Figure S2/Supplementary_Figure_S2_191.tif]

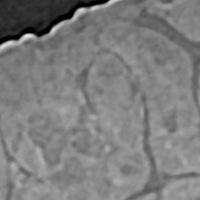

Supplement: Supplementary file 3 — Supplementary Information 3. [file 41598_2022_13394_MOESM3_ESM.zip › Supplementary Figure S2/Supplementary_Figure_S2_192.tif]

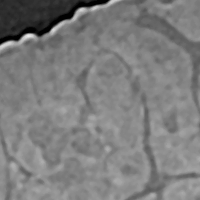

Supplement: Supplementary file 3 — Supplementary Information 3. [file 41598_2022_13394_MOESM3_ESM.zip › Supplementary Figure S2/Supplementary_Figure_S2_193.tif]

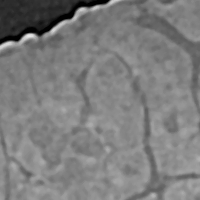

Supplement: Supplementary file 3 — Supplementary Information 3. [file 41598_2022_13394_MOESM3_ESM.zip › Supplementary Figure S2/Supplementary_Figure_S2_194.tif]

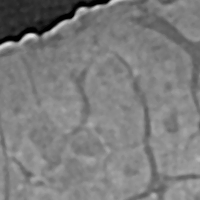

Supplement: Supplementary file 3 — Supplementary Information 3. [file 41598_2022_13394_MOESM3_ESM.zip › Supplementary Figure S2/Supplementary_Figure_S2_195.tif]

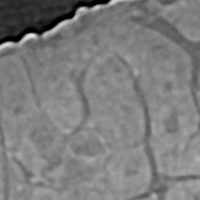

Supplement: Supplementary file 3 — Supplementary Information 3. [file 41598_2022_13394_MOESM3_ESM.zip › Supplementary Figure S2/Supplementary_Figure_S2_196.tif]

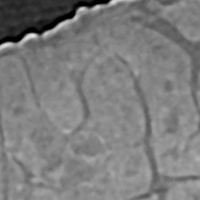

Supplement: Supplementary file 3 — Supplementary Information 3. [file 41598_2022_13394_MOESM3_ESM.zip › Supplementary Figure S2/Supplementary_Figure_S2_197.tif]

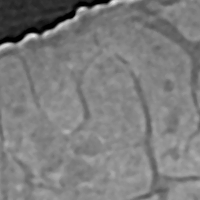

Supplement: Supplementary file 3 — Supplementary Information 3. [file 41598_2022_13394_MOESM3_ESM.zip › Supplementary Figure S2/Supplementary_Figure_S2_198.tif]
