# Supplementary figures and images for: Nondestructive cellular-level 3D observation of mouse kidney using laboratory-based X-ray microscopy with paraffin-mediated contrast enhancement (part 5 of 9)
Source: Sci Rep. 2022 Jun 8;12:9436. doi: 10.1038/s41598-022-13394-9 (PMC9177607; doi:10.1038/s41598-022-13394-9)

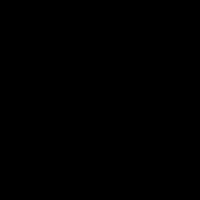

Supplement: Supplementary file 4 — Supplementary Information 4. [file 41598_2022_13394_MOESM4_ESM.zip › Supplementary Figure S3/Supplementary_Figure_S3_199.tif]

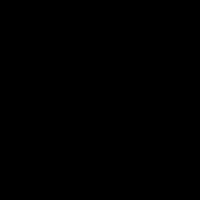

Supplement: Supplementary file 4 — Supplementary Information 4. [file 41598_2022_13394_MOESM4_ESM.zip › Supplementary Figure S3/Supplementary_Figure_S3_200.tif]

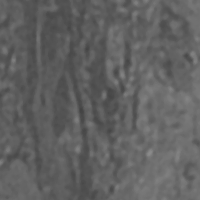

Supplement: Supplementary file 5 — Supplementary Information 5. [file 41598_2022_13394_MOESM5_ESM.zip › Supplementary Figure S4/Supplementary_Figure_S4_001.tif]

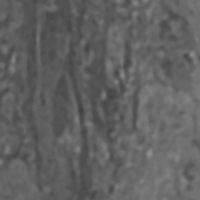

Supplement: Supplementary file 5 — Supplementary Information 5. [file 41598_2022_13394_MOESM5_ESM.zip › Supplementary Figure S4/Supplementary_Figure_S4_002.tif]

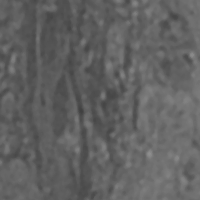

Supplement: Supplementary file 5 — Supplementary Information 5. [file 41598_2022_13394_MOESM5_ESM.zip › Supplementary Figure S4/Supplementary_Figure_S4_003.tif]

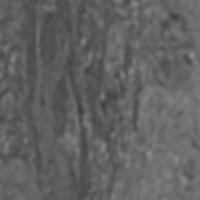

Supplement: Supplementary file 5 — Supplementary Information 5. [file 41598_2022_13394_MOESM5_ESM.zip › Supplementary Figure S4/Supplementary_Figure_S4_004.tif]

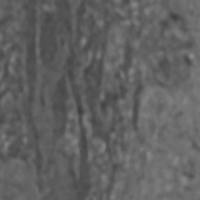

Supplement: Supplementary file 5 — Supplementary Information 5. [file 41598_2022_13394_MOESM5_ESM.zip › Supplementary Figure S4/Supplementary_Figure_S4_005.tif]

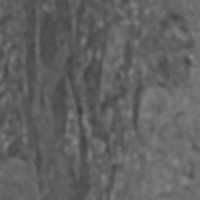

Supplement: Supplementary file 5 — Supplementary Information 5. [file 41598_2022_13394_MOESM5_ESM.zip › Supplementary Figure S4/Supplementary_Figure_S4_006.tif]

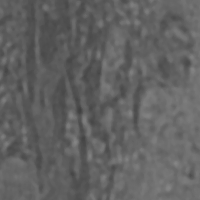

Supplement: Supplementary file 5 — Supplementary Information 5. [file 41598_2022_13394_MOESM5_ESM.zip › Supplementary Figure S4/Supplementary_Figure_S4_007.tif]

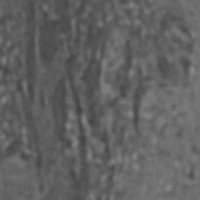

Supplement: Supplementary file 5 — Supplementary Information 5. [file 41598_2022_13394_MOESM5_ESM.zip › Supplementary Figure S4/Supplementary_Figure_S4_008.tif]

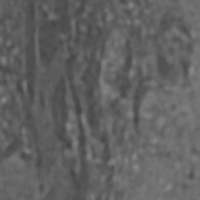

Supplement: Supplementary file 5 — Supplementary Information 5. [file 41598_2022_13394_MOESM5_ESM.zip › Supplementary Figure S4/Supplementary_Figure_S4_009.tif]

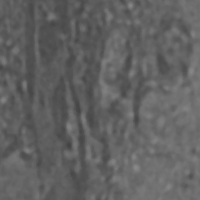

Supplement: Supplementary file 5 — Supplementary Information 5. [file 41598_2022_13394_MOESM5_ESM.zip › Supplementary Figure S4/Supplementary_Figure_S4_010.tif]

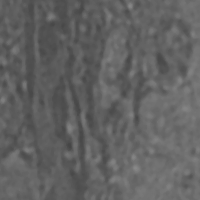

Supplement: Supplementary file 5 — Supplementary Information 5. [file 41598_2022_13394_MOESM5_ESM.zip › Supplementary Figure S4/Supplementary_Figure_S4_011.tif]

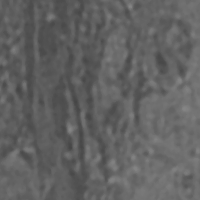

Supplement: Supplementary file 5 — Supplementary Information 5. [file 41598_2022_13394_MOESM5_ESM.zip › Supplementary Figure S4/Supplementary_Figure_S4_012.tif]

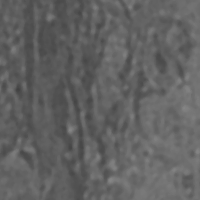

Supplement: Supplementary file 5 — Supplementary Information 5. [file 41598_2022_13394_MOESM5_ESM.zip › Supplementary Figure S4/Supplementary_Figure_S4_013.tif]

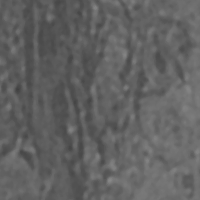

Supplement: Supplementary file 5 — Supplementary Information 5. [file 41598_2022_13394_MOESM5_ESM.zip › Supplementary Figure S4/Supplementary_Figure_S4_014.tif]

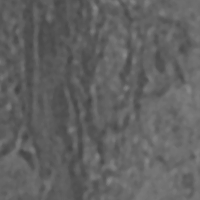

Supplement: Supplementary file 5 — Supplementary Information 5. [file 41598_2022_13394_MOESM5_ESM.zip › Supplementary Figure S4/Supplementary_Figure_S4_015.tif]

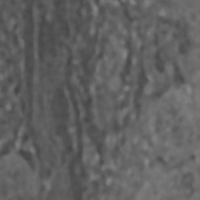

Supplement: Supplementary file 5 — Supplementary Information 5. [file 41598_2022_13394_MOESM5_ESM.zip › Supplementary Figure S4/Supplementary_Figure_S4_016.tif]

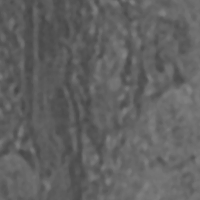

Supplement: Supplementary file 5 — Supplementary Information 5. [file 41598_2022_13394_MOESM5_ESM.zip › Supplementary Figure S4/Supplementary_Figure_S4_017.tif]

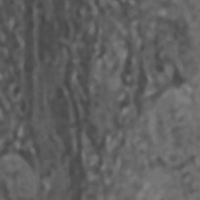

Supplement: Supplementary file 5 — Supplementary Information 5. [file 41598_2022_13394_MOESM5_ESM.zip › Supplementary Figure S4/Supplementary_Figure_S4_018.tif]

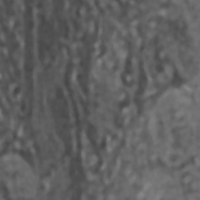

Supplement: Supplementary file 5 — Supplementary Information 5. [file 41598_2022_13394_MOESM5_ESM.zip › Supplementary Figure S4/Supplementary_Figure_S4_019.tif]

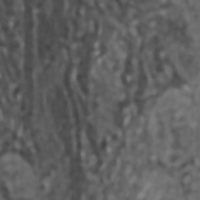

Supplement: Supplementary file 5 — Supplementary Information 5. [file 41598_2022_13394_MOESM5_ESM.zip › Supplementary Figure S4/Supplementary_Figure_S4_020.tif]

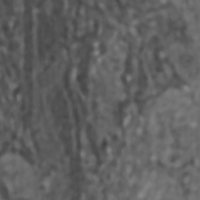

Supplement: Supplementary file 5 — Supplementary Information 5. [file 41598_2022_13394_MOESM5_ESM.zip › Supplementary Figure S4/Supplementary_Figure_S4_021.tif]

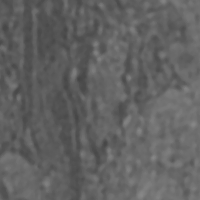

Supplement: Supplementary file 5 — Supplementary Information 5. [file 41598_2022_13394_MOESM5_ESM.zip › Supplementary Figure S4/Supplementary_Figure_S4_022.tif]

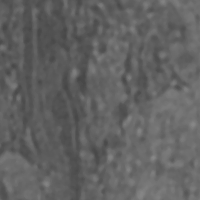

Supplement: Supplementary file 5 — Supplementary Information 5. [file 41598_2022_13394_MOESM5_ESM.zip › Supplementary Figure S4/Supplementary_Figure_S4_023.tif]

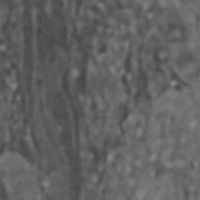

Supplement: Supplementary file 5 — Supplementary Information 5. [file 41598_2022_13394_MOESM5_ESM.zip › Supplementary Figure S4/Supplementary_Figure_S4_024.tif]

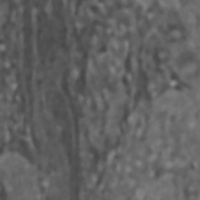

Supplement: Supplementary file 5 — Supplementary Information 5. [file 41598_2022_13394_MOESM5_ESM.zip › Supplementary Figure S4/Supplementary_Figure_S4_025.tif]

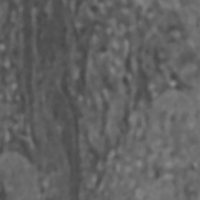

Supplement: Supplementary file 5 — Supplementary Information 5. [file 41598_2022_13394_MOESM5_ESM.zip › Supplementary Figure S4/Supplementary_Figure_S4_026.tif]

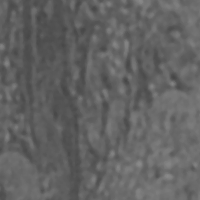

Supplement: Supplementary file 5 — Supplementary Information 5. [file 41598_2022_13394_MOESM5_ESM.zip › Supplementary Figure S4/Supplementary_Figure_S4_027.tif]

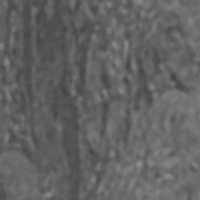

Supplement: Supplementary file 5 — Supplementary Information 5. [file 41598_2022_13394_MOESM5_ESM.zip › Supplementary Figure S4/Supplementary_Figure_S4_028.tif]

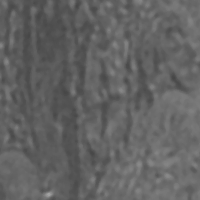

Supplement: Supplementary file 5 — Supplementary Information 5. [file 41598_2022_13394_MOESM5_ESM.zip › Supplementary Figure S4/Supplementary_Figure_S4_029.tif]

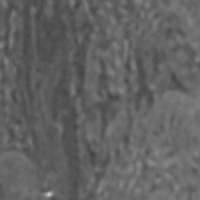

Supplement: Supplementary file 5 — Supplementary Information 5. [file 41598_2022_13394_MOESM5_ESM.zip › Supplementary Figure S4/Supplementary_Figure_S4_030.tif]

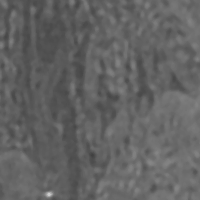

Supplement: Supplementary file 5 — Supplementary Information 5. [file 41598_2022_13394_MOESM5_ESM.zip › Supplementary Figure S4/Supplementary_Figure_S4_031.tif]

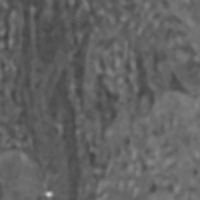

Supplement: Supplementary file 5 — Supplementary Information 5. [file 41598_2022_13394_MOESM5_ESM.zip › Supplementary Figure S4/Supplementary_Figure_S4_032.tif]

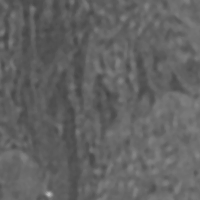

Supplement: Supplementary file 5 — Supplementary Information 5. [file 41598_2022_13394_MOESM5_ESM.zip › Supplementary Figure S4/Supplementary_Figure_S4_033.tif]

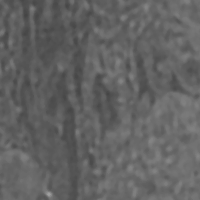

Supplement: Supplementary file 5 — Supplementary Information 5. [file 41598_2022_13394_MOESM5_ESM.zip › Supplementary Figure S4/Supplementary_Figure_S4_034.tif]

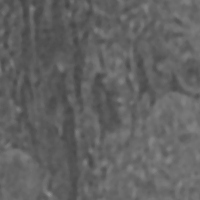

Supplement: Supplementary file 5 — Supplementary Information 5. [file 41598_2022_13394_MOESM5_ESM.zip › Supplementary Figure S4/Supplementary_Figure_S4_035.tif]

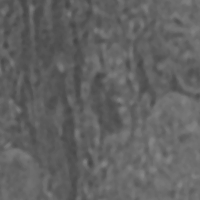

Supplement: Supplementary file 5 — Supplementary Information 5. [file 41598_2022_13394_MOESM5_ESM.zip › Supplementary Figure S4/Supplementary_Figure_S4_036.tif]

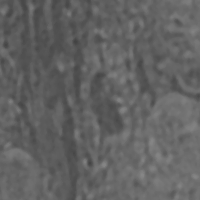

Supplement: Supplementary file 5 — Supplementary Information 5. [file 41598_2022_13394_MOESM5_ESM.zip › Supplementary Figure S4/Supplementary_Figure_S4_037.tif]

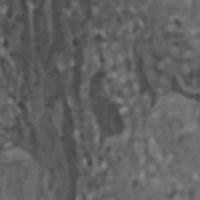

Supplement: Supplementary file 5 — Supplementary Information 5. [file 41598_2022_13394_MOESM5_ESM.zip › Supplementary Figure S4/Supplementary_Figure_S4_038.tif]

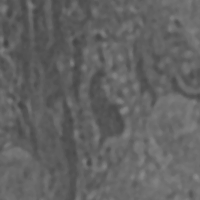

Supplement: Supplementary file 5 — Supplementary Information 5. [file 41598_2022_13394_MOESM5_ESM.zip › Supplementary Figure S4/Supplementary_Figure_S4_039.tif]

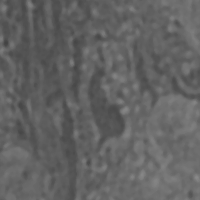

Supplement: Supplementary file 5 — Supplementary Information 5. [file 41598_2022_13394_MOESM5_ESM.zip › Supplementary Figure S4/Supplementary_Figure_S4_040.tif]

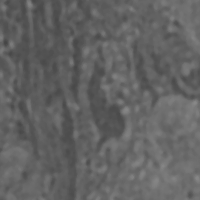

Supplement: Supplementary file 5 — Supplementary Information 5. [file 41598_2022_13394_MOESM5_ESM.zip › Supplementary Figure S4/Supplementary_Figure_S4_041.tif]

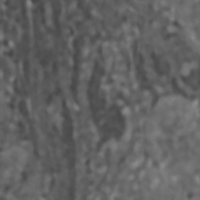

Supplement: Supplementary file 5 — Supplementary Information 5. [file 41598_2022_13394_MOESM5_ESM.zip › Supplementary Figure S4/Supplementary_Figure_S4_042.tif]

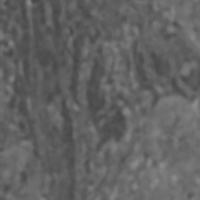

Supplement: Supplementary file 5 — Supplementary Information 5. [file 41598_2022_13394_MOESM5_ESM.zip › Supplementary Figure S4/Supplementary_Figure_S4_043.tif]

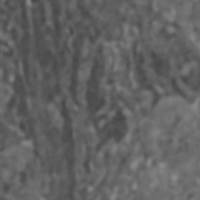

Supplement: Supplementary file 5 — Supplementary Information 5. [file 41598_2022_13394_MOESM5_ESM.zip › Supplementary Figure S4/Supplementary_Figure_S4_044.tif]

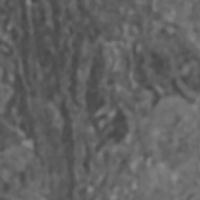

Supplement: Supplementary file 5 — Supplementary Information 5. [file 41598_2022_13394_MOESM5_ESM.zip › Supplementary Figure S4/Supplementary_Figure_S4_045.tif]

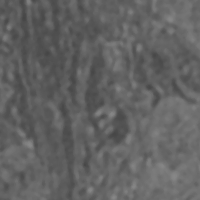

Supplement: Supplementary file 5 — Supplementary Information 5. [file 41598_2022_13394_MOESM5_ESM.zip › Supplementary Figure S4/Supplementary_Figure_S4_046.tif]

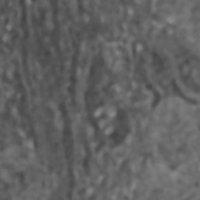

Supplement: Supplementary file 5 — Supplementary Information 5. [file 41598_2022_13394_MOESM5_ESM.zip › Supplementary Figure S4/Supplementary_Figure_S4_047.tif]

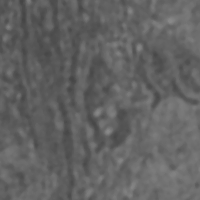

Supplement: Supplementary file 5 — Supplementary Information 5. [file 41598_2022_13394_MOESM5_ESM.zip › Supplementary Figure S4/Supplementary_Figure_S4_048.tif]

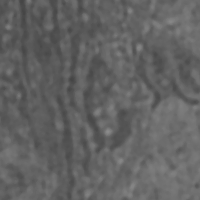

Supplement: Supplementary file 5 — Supplementary Information 5. [file 41598_2022_13394_MOESM5_ESM.zip › Supplementary Figure S4/Supplementary_Figure_S4_049.tif]

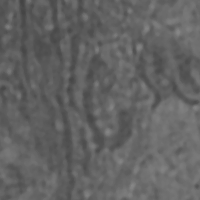

Supplement: Supplementary file 5 — Supplementary Information 5. [file 41598_2022_13394_MOESM5_ESM.zip › Supplementary Figure S4/Supplementary_Figure_S4_050.tif]

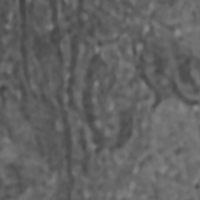

Supplement: Supplementary file 5 — Supplementary Information 5. [file 41598_2022_13394_MOESM5_ESM.zip › Supplementary Figure S4/Supplementary_Figure_S4_051.tif]

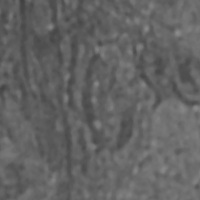

Supplement: Supplementary file 5 — Supplementary Information 5. [file 41598_2022_13394_MOESM5_ESM.zip › Supplementary Figure S4/Supplementary_Figure_S4_052.tif]

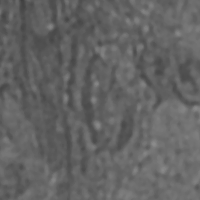

Supplement: Supplementary file 5 — Supplementary Information 5. [file 41598_2022_13394_MOESM5_ESM.zip › Supplementary Figure S4/Supplementary_Figure_S4_053.tif]

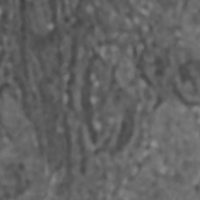

Supplement: Supplementary file 5 — Supplementary Information 5. [file 41598_2022_13394_MOESM5_ESM.zip › Supplementary Figure S4/Supplementary_Figure_S4_054.tif]

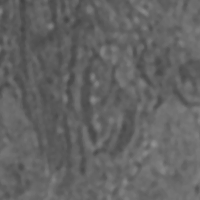

Supplement: Supplementary file 5 — Supplementary Information 5. [file 41598_2022_13394_MOESM5_ESM.zip › Supplementary Figure S4/Supplementary_Figure_S4_055.tif]

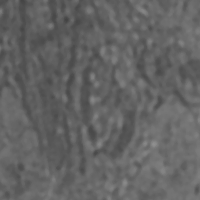

Supplement: Supplementary file 5 — Supplementary Information 5. [file 41598_2022_13394_MOESM5_ESM.zip › Supplementary Figure S4/Supplementary_Figure_S4_056.tif]

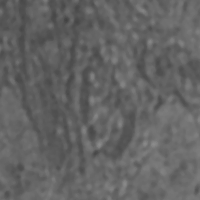

Supplement: Supplementary file 5 — Supplementary Information 5. [file 41598_2022_13394_MOESM5_ESM.zip › Supplementary Figure S4/Supplementary_Figure_S4_057.tif]

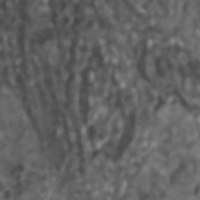

Supplement: Supplementary file 5 — Supplementary Information 5. [file 41598_2022_13394_MOESM5_ESM.zip › Supplementary Figure S4/Supplementary_Figure_S4_058.tif]

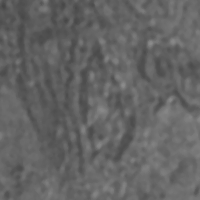

Supplement: Supplementary file 5 — Supplementary Information 5. [file 41598_2022_13394_MOESM5_ESM.zip › Supplementary Figure S4/Supplementary_Figure_S4_059.tif]

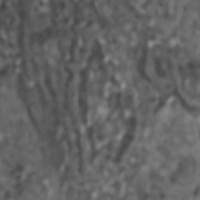

Supplement: Supplementary file 5 — Supplementary Information 5. [file 41598_2022_13394_MOESM5_ESM.zip › Supplementary Figure S4/Supplementary_Figure_S4_060.tif]

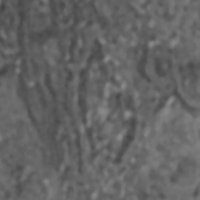

Supplement: Supplementary file 5 — Supplementary Information 5. [file 41598_2022_13394_MOESM5_ESM.zip › Supplementary Figure S4/Supplementary_Figure_S4_061.tif]

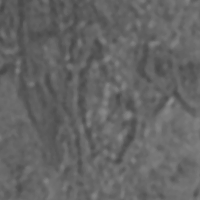

Supplement: Supplementary file 5 — Supplementary Information 5. [file 41598_2022_13394_MOESM5_ESM.zip › Supplementary Figure S4/Supplementary_Figure_S4_062.tif]

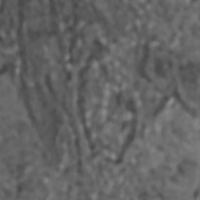

Supplement: Supplementary file 5 — Supplementary Information 5. [file 41598_2022_13394_MOESM5_ESM.zip › Supplementary Figure S4/Supplementary_Figure_S4_063.tif]

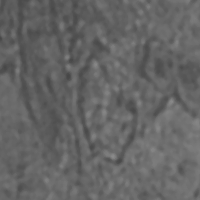

Supplement: Supplementary file 5 — Supplementary Information 5. [file 41598_2022_13394_MOESM5_ESM.zip › Supplementary Figure S4/Supplementary_Figure_S4_064.tif]

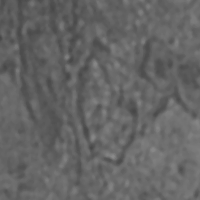

Supplement: Supplementary file 5 — Supplementary Information 5. [file 41598_2022_13394_MOESM5_ESM.zip › Supplementary Figure S4/Supplementary_Figure_S4_065.tif]

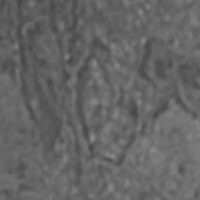

Supplement: Supplementary file 5 — Supplementary Information 5. [file 41598_2022_13394_MOESM5_ESM.zip › Supplementary Figure S4/Supplementary_Figure_S4_066.tif]

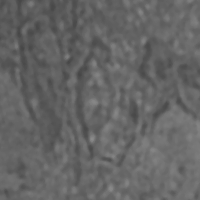

Supplement: Supplementary file 5 — Supplementary Information 5. [file 41598_2022_13394_MOESM5_ESM.zip › Supplementary Figure S4/Supplementary_Figure_S4_067.tif]

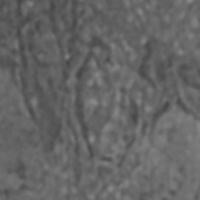

Supplement: Supplementary file 5 — Supplementary Information 5. [file 41598_2022_13394_MOESM5_ESM.zip › Supplementary Figure S4/Supplementary_Figure_S4_068.tif]

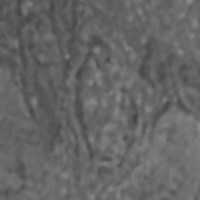

Supplement: Supplementary file 5 — Supplementary Information 5. [file 41598_2022_13394_MOESM5_ESM.zip › Supplementary Figure S4/Supplementary_Figure_S4_069.tif]

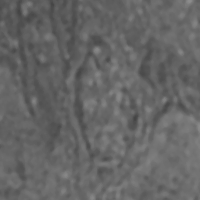

Supplement: Supplementary file 5 — Supplementary Information 5. [file 41598_2022_13394_MOESM5_ESM.zip › Supplementary Figure S4/Supplementary_Figure_S4_070.tif]

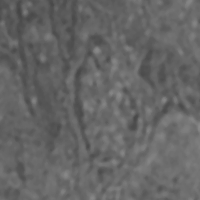

Supplement: Supplementary file 5 — Supplementary Information 5. [file 41598_2022_13394_MOESM5_ESM.zip › Supplementary Figure S4/Supplementary_Figure_S4_071.tif]

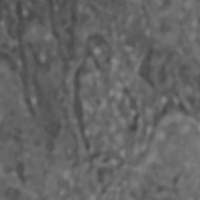

Supplement: Supplementary file 5 — Supplementary Information 5. [file 41598_2022_13394_MOESM5_ESM.zip › Supplementary Figure S4/Supplementary_Figure_S4_072.tif]

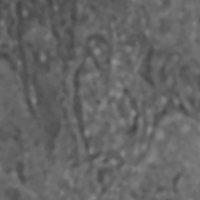

Supplement: Supplementary file 5 — Supplementary Information 5. [file 41598_2022_13394_MOESM5_ESM.zip › Supplementary Figure S4/Supplementary_Figure_S4_073.tif]

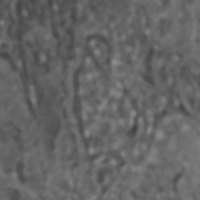

Supplement: Supplementary file 5 — Supplementary Information 5. [file 41598_2022_13394_MOESM5_ESM.zip › Supplementary Figure S4/Supplementary_Figure_S4_074.tif]

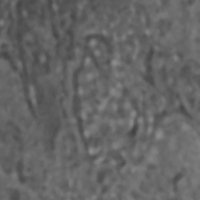

Supplement: Supplementary file 5 — Supplementary Information 5. [file 41598_2022_13394_MOESM5_ESM.zip › Supplementary Figure S4/Supplementary_Figure_S4_075.tif]

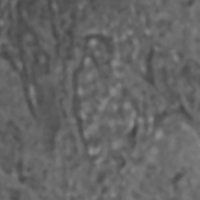

Supplement: Supplementary file 5 — Supplementary Information 5. [file 41598_2022_13394_MOESM5_ESM.zip › Supplementary Figure S4/Supplementary_Figure_S4_076.tif]

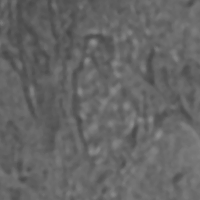

Supplement: Supplementary file 5 — Supplementary Information 5. [file 41598_2022_13394_MOESM5_ESM.zip › Supplementary Figure S4/Supplementary_Figure_S4_077.tif]

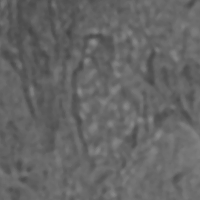

Supplement: Supplementary file 5 — Supplementary Information 5. [file 41598_2022_13394_MOESM5_ESM.zip › Supplementary Figure S4/Supplementary_Figure_S4_078.tif]

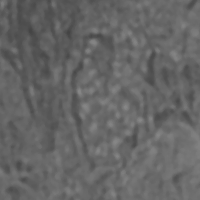

Supplement: Supplementary file 5 — Supplementary Information 5. [file 41598_2022_13394_MOESM5_ESM.zip › Supplementary Figure S4/Supplementary_Figure_S4_079.tif]

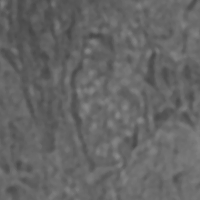

Supplement: Supplementary file 5 — Supplementary Information 5. [file 41598_2022_13394_MOESM5_ESM.zip › Supplementary Figure S4/Supplementary_Figure_S4_080.tif]

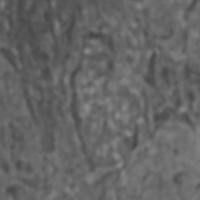

Supplement: Supplementary file 5 — Supplementary Information 5. [file 41598_2022_13394_MOESM5_ESM.zip › Supplementary Figure S4/Supplementary_Figure_S4_081.tif]

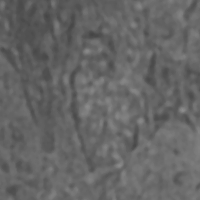

Supplement: Supplementary file 5 — Supplementary Information 5. [file 41598_2022_13394_MOESM5_ESM.zip › Supplementary Figure S4/Supplementary_Figure_S4_082.tif]

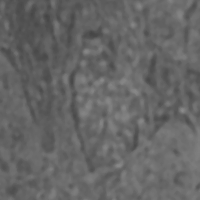

Supplement: Supplementary file 5 — Supplementary Information 5. [file 41598_2022_13394_MOESM5_ESM.zip › Supplementary Figure S4/Supplementary_Figure_S4_083.tif]

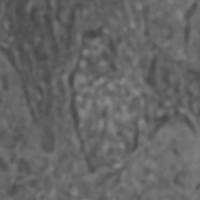

Supplement: Supplementary file 5 — Supplementary Information 5. [file 41598_2022_13394_MOESM5_ESM.zip › Supplementary Figure S4/Supplementary_Figure_S4_084.tif]

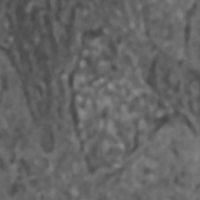

Supplement: Supplementary file 5 — Supplementary Information 5. [file 41598_2022_13394_MOESM5_ESM.zip › Supplementary Figure S4/Supplementary_Figure_S4_085.tif]

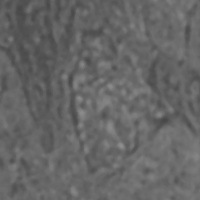

Supplement: Supplementary file 5 — Supplementary Information 5. [file 41598_2022_13394_MOESM5_ESM.zip › Supplementary Figure S4/Supplementary_Figure_S4_086.tif]

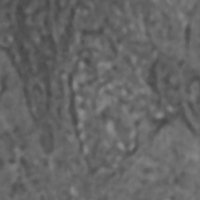

Supplement: Supplementary file 5 — Supplementary Information 5. [file 41598_2022_13394_MOESM5_ESM.zip › Supplementary Figure S4/Supplementary_Figure_S4_087.tif]

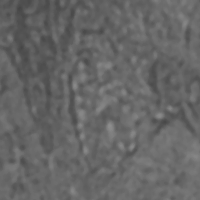

Supplement: Supplementary file 5 — Supplementary Information 5. [file 41598_2022_13394_MOESM5_ESM.zip › Supplementary Figure S4/Supplementary_Figure_S4_088.tif]

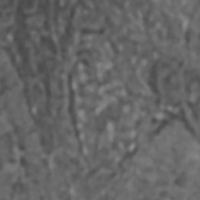

Supplement: Supplementary file 5 — Supplementary Information 5. [file 41598_2022_13394_MOESM5_ESM.zip › Supplementary Figure S4/Supplementary_Figure_S4_089.tif]

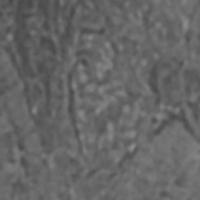

Supplement: Supplementary file 5 — Supplementary Information 5. [file 41598_2022_13394_MOESM5_ESM.zip › Supplementary Figure S4/Supplementary_Figure_S4_090.tif]

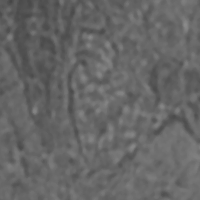

Supplement: Supplementary file 5 — Supplementary Information 5. [file 41598_2022_13394_MOESM5_ESM.zip › Supplementary Figure S4/Supplementary_Figure_S4_091.tif]

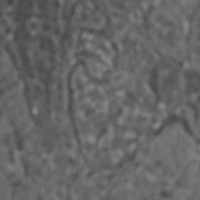

Supplement: Supplementary file 5 — Supplementary Information 5. [file 41598_2022_13394_MOESM5_ESM.zip › Supplementary Figure S4/Supplementary_Figure_S4_092.tif]

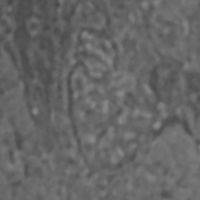

Supplement: Supplementary file 5 — Supplementary Information 5. [file 41598_2022_13394_MOESM5_ESM.zip › Supplementary Figure S4/Supplementary_Figure_S4_093.tif]

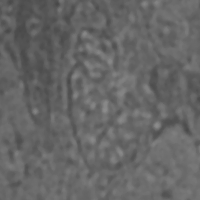

Supplement: Supplementary file 5 — Supplementary Information 5. [file 41598_2022_13394_MOESM5_ESM.zip › Supplementary Figure S4/Supplementary_Figure_S4_094.tif]

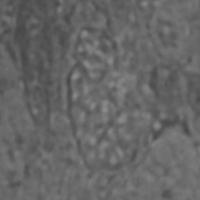

Supplement: Supplementary file 5 — Supplementary Information 5. [file 41598_2022_13394_MOESM5_ESM.zip › Supplementary Figure S4/Supplementary_Figure_S4_095.tif]

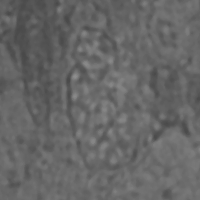

Supplement: Supplementary file 5 — Supplementary Information 5. [file 41598_2022_13394_MOESM5_ESM.zip › Supplementary Figure S4/Supplementary_Figure_S4_096.tif]

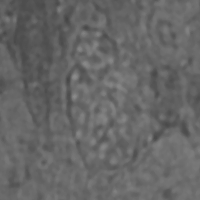

Supplement: Supplementary file 5 — Supplementary Information 5. [file 41598_2022_13394_MOESM5_ESM.zip › Supplementary Figure S4/Supplementary_Figure_S4_097.tif]

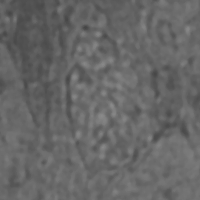

Supplement: Supplementary file 5 — Supplementary Information 5. [file 41598_2022_13394_MOESM5_ESM.zip › Supplementary Figure S4/Supplementary_Figure_S4_098.tif]
