# Supplementary figures and images for: Nondestructive cellular-level 3D observation of mouse kidney using laboratory-based X-ray microscopy with paraffin-mediated contrast enhancement (part 4 of 9)
Source: Sci Rep. 2022 Jun 8;12:9436. doi: 10.1038/s41598-022-13394-9 (PMC9177607; doi:10.1038/s41598-022-13394-9)

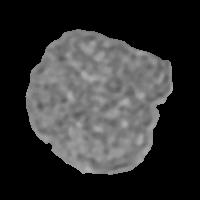

Supplement: Supplementary file 4 — Supplementary Information 4. [file 41598_2022_13394_MOESM4_ESM.zip › Supplementary Figure S3/Supplementary_Figure_S3_099.tif]

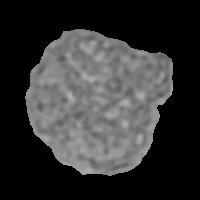

Supplement: Supplementary file 4 — Supplementary Information 4. [file 41598_2022_13394_MOESM4_ESM.zip › Supplementary Figure S3/Supplementary_Figure_S3_100.tif]

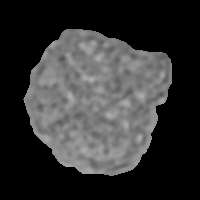

Supplement: Supplementary file 4 — Supplementary Information 4. [file 41598_2022_13394_MOESM4_ESM.zip › Supplementary Figure S3/Supplementary_Figure_S3_101.tif]

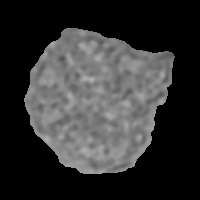

Supplement: Supplementary file 4 — Supplementary Information 4. [file 41598_2022_13394_MOESM4_ESM.zip › Supplementary Figure S3/Supplementary_Figure_S3_102.tif]

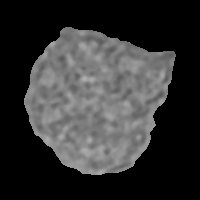

Supplement: Supplementary file 4 — Supplementary Information 4. [file 41598_2022_13394_MOESM4_ESM.zip › Supplementary Figure S3/Supplementary_Figure_S3_103.tif]

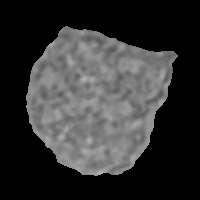

Supplement: Supplementary file 4 — Supplementary Information 4. [file 41598_2022_13394_MOESM4_ESM.zip › Supplementary Figure S3/Supplementary_Figure_S3_104.tif]

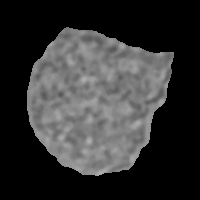

Supplement: Supplementary file 4 — Supplementary Information 4. [file 41598_2022_13394_MOESM4_ESM.zip › Supplementary Figure S3/Supplementary_Figure_S3_105.tif]

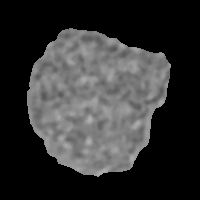

Supplement: Supplementary file 4 — Supplementary Information 4. [file 41598_2022_13394_MOESM4_ESM.zip › Supplementary Figure S3/Supplementary_Figure_S3_106.tif]

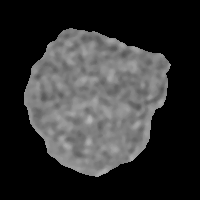

Supplement: Supplementary file 4 — Supplementary Information 4. [file 41598_2022_13394_MOESM4_ESM.zip › Supplementary Figure S3/Supplementary_Figure_S3_107.tif]

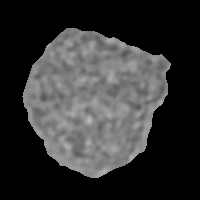

Supplement: Supplementary file 4 — Supplementary Information 4. [file 41598_2022_13394_MOESM4_ESM.zip › Supplementary Figure S3/Supplementary_Figure_S3_108.tif]

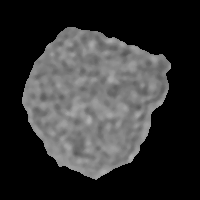

Supplement: Supplementary file 4 — Supplementary Information 4. [file 41598_2022_13394_MOESM4_ESM.zip › Supplementary Figure S3/Supplementary_Figure_S3_109.tif]

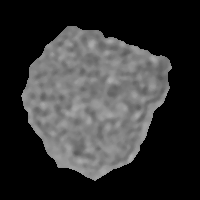

Supplement: Supplementary file 4 — Supplementary Information 4. [file 41598_2022_13394_MOESM4_ESM.zip › Supplementary Figure S3/Supplementary_Figure_S3_110.tif]

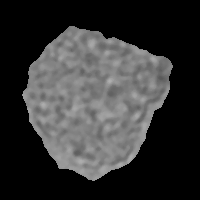

Supplement: Supplementary file 4 — Supplementary Information 4. [file 41598_2022_13394_MOESM4_ESM.zip › Supplementary Figure S3/Supplementary_Figure_S3_111.tif]

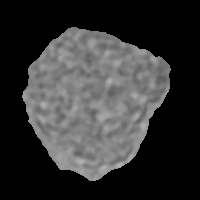

Supplement: Supplementary file 4 — Supplementary Information 4. [file 41598_2022_13394_MOESM4_ESM.zip › Supplementary Figure S3/Supplementary_Figure_S3_112.tif]

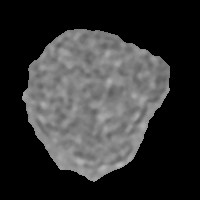

Supplement: Supplementary file 4 — Supplementary Information 4. [file 41598_2022_13394_MOESM4_ESM.zip › Supplementary Figure S3/Supplementary_Figure_S3_113.tif]

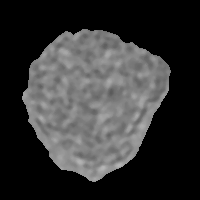

Supplement: Supplementary file 4 — Supplementary Information 4. [file 41598_2022_13394_MOESM4_ESM.zip › Supplementary Figure S3/Supplementary_Figure_S3_114.tif]

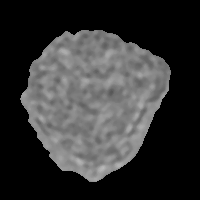

Supplement: Supplementary file 4 — Supplementary Information 4. [file 41598_2022_13394_MOESM4_ESM.zip › Supplementary Figure S3/Supplementary_Figure_S3_115.tif]

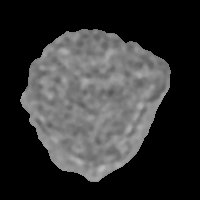

Supplement: Supplementary file 4 — Supplementary Information 4. [file 41598_2022_13394_MOESM4_ESM.zip › Supplementary Figure S3/Supplementary_Figure_S3_116.tif]

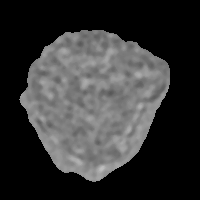

Supplement: Supplementary file 4 — Supplementary Information 4. [file 41598_2022_13394_MOESM4_ESM.zip › Supplementary Figure S3/Supplementary_Figure_S3_117.tif]

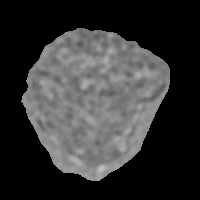

Supplement: Supplementary file 4 — Supplementary Information 4. [file 41598_2022_13394_MOESM4_ESM.zip › Supplementary Figure S3/Supplementary_Figure_S3_118.tif]

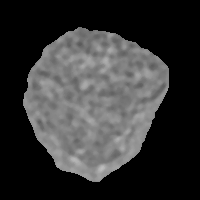

Supplement: Supplementary file 4 — Supplementary Information 4. [file 41598_2022_13394_MOESM4_ESM.zip › Supplementary Figure S3/Supplementary_Figure_S3_119.tif]

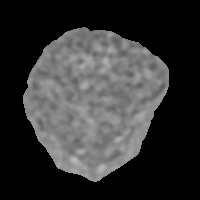

Supplement: Supplementary file 4 — Supplementary Information 4. [file 41598_2022_13394_MOESM4_ESM.zip › Supplementary Figure S3/Supplementary_Figure_S3_120.tif]

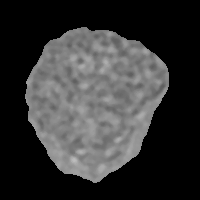

Supplement: Supplementary file 4 — Supplementary Information 4. [file 41598_2022_13394_MOESM4_ESM.zip › Supplementary Figure S3/Supplementary_Figure_S3_121.tif]

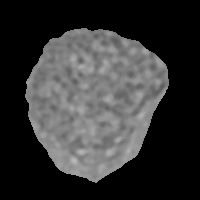

Supplement: Supplementary file 4 — Supplementary Information 4. [file 41598_2022_13394_MOESM4_ESM.zip › Supplementary Figure S3/Supplementary_Figure_S3_122.tif]

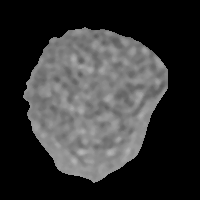

Supplement: Supplementary file 4 — Supplementary Information 4. [file 41598_2022_13394_MOESM4_ESM.zip › Supplementary Figure S3/Supplementary_Figure_S3_123.tif]

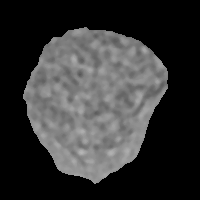

Supplement: Supplementary file 4 — Supplementary Information 4. [file 41598_2022_13394_MOESM4_ESM.zip › Supplementary Figure S3/Supplementary_Figure_S3_124.tif]

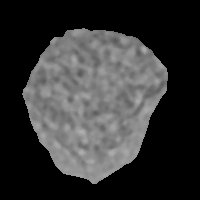

Supplement: Supplementary file 4 — Supplementary Information 4. [file 41598_2022_13394_MOESM4_ESM.zip › Supplementary Figure S3/Supplementary_Figure_S3_125.tif]

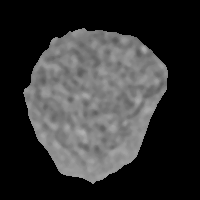

Supplement: Supplementary file 4 — Supplementary Information 4. [file 41598_2022_13394_MOESM4_ESM.zip › Supplementary Figure S3/Supplementary_Figure_S3_126.tif]

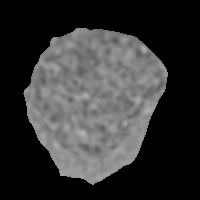

Supplement: Supplementary file 4 — Supplementary Information 4. [file 41598_2022_13394_MOESM4_ESM.zip › Supplementary Figure S3/Supplementary_Figure_S3_127.tif]

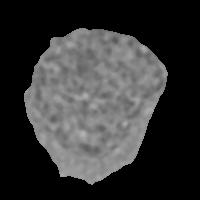

Supplement: Supplementary file 4 — Supplementary Information 4. [file 41598_2022_13394_MOESM4_ESM.zip › Supplementary Figure S3/Supplementary_Figure_S3_128.tif]

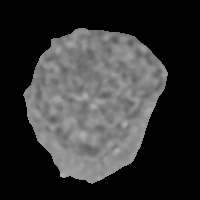

Supplement: Supplementary file 4 — Supplementary Information 4. [file 41598_2022_13394_MOESM4_ESM.zip › Supplementary Figure S3/Supplementary_Figure_S3_129.tif]

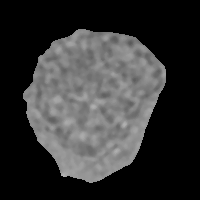

Supplement: Supplementary file 4 — Supplementary Information 4. [file 41598_2022_13394_MOESM4_ESM.zip › Supplementary Figure S3/Supplementary_Figure_S3_130.tif]

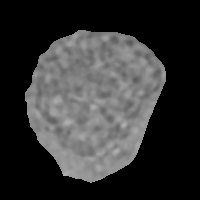

Supplement: Supplementary file 4 — Supplementary Information 4. [file 41598_2022_13394_MOESM4_ESM.zip › Supplementary Figure S3/Supplementary_Figure_S3_131.tif]

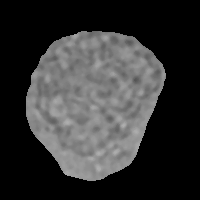

Supplement: Supplementary file 4 — Supplementary Information 4. [file 41598_2022_13394_MOESM4_ESM.zip › Supplementary Figure S3/Supplementary_Figure_S3_132.tif]

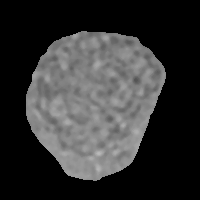

Supplement: Supplementary file 4 — Supplementary Information 4. [file 41598_2022_13394_MOESM4_ESM.zip › Supplementary Figure S3/Supplementary_Figure_S3_133.tif]

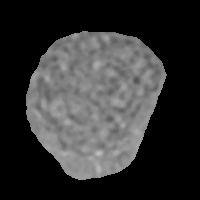

Supplement: Supplementary file 4 — Supplementary Information 4. [file 41598_2022_13394_MOESM4_ESM.zip › Supplementary Figure S3/Supplementary_Figure_S3_134.tif]

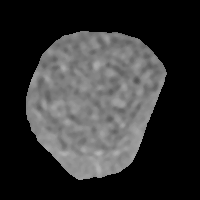

Supplement: Supplementary file 4 — Supplementary Information 4. [file 41598_2022_13394_MOESM4_ESM.zip › Supplementary Figure S3/Supplementary_Figure_S3_135.tif]

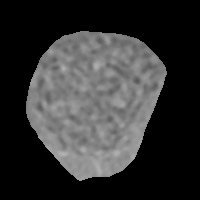

Supplement: Supplementary file 4 — Supplementary Information 4. [file 41598_2022_13394_MOESM4_ESM.zip › Supplementary Figure S3/Supplementary_Figure_S3_136.tif]

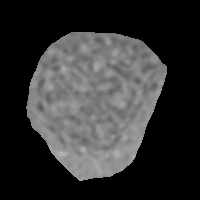

Supplement: Supplementary file 4 — Supplementary Information 4. [file 41598_2022_13394_MOESM4_ESM.zip › Supplementary Figure S3/Supplementary_Figure_S3_137.tif]

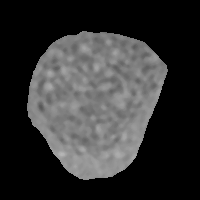

Supplement: Supplementary file 4 — Supplementary Information 4. [file 41598_2022_13394_MOESM4_ESM.zip › Supplementary Figure S3/Supplementary_Figure_S3_138.tif]

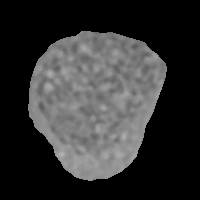

Supplement: Supplementary file 4 — Supplementary Information 4. [file 41598_2022_13394_MOESM4_ESM.zip › Supplementary Figure S3/Supplementary_Figure_S3_139.tif]

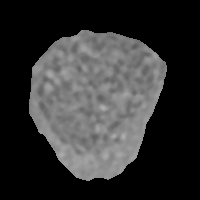

Supplement: Supplementary file 4 — Supplementary Information 4. [file 41598_2022_13394_MOESM4_ESM.zip › Supplementary Figure S3/Supplementary_Figure_S3_140.tif]

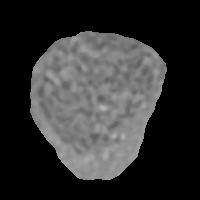

Supplement: Supplementary file 4 — Supplementary Information 4. [file 41598_2022_13394_MOESM4_ESM.zip › Supplementary Figure S3/Supplementary_Figure_S3_141.tif]

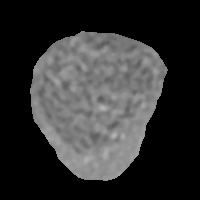

Supplement: Supplementary file 4 — Supplementary Information 4. [file 41598_2022_13394_MOESM4_ESM.zip › Supplementary Figure S3/Supplementary_Figure_S3_142.tif]

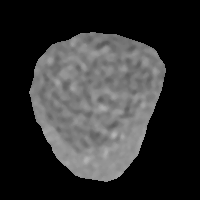

Supplement: Supplementary file 4 — Supplementary Information 4. [file 41598_2022_13394_MOESM4_ESM.zip › Supplementary Figure S3/Supplementary_Figure_S3_143.tif]

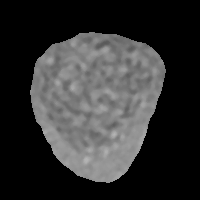

Supplement: Supplementary file 4 — Supplementary Information 4. [file 41598_2022_13394_MOESM4_ESM.zip › Supplementary Figure S3/Supplementary_Figure_S3_144.tif]

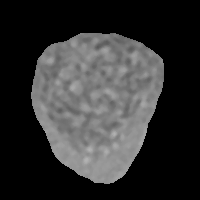

Supplement: Supplementary file 4 — Supplementary Information 4. [file 41598_2022_13394_MOESM4_ESM.zip › Supplementary Figure S3/Supplementary_Figure_S3_145.tif]

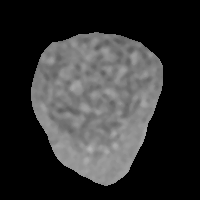

Supplement: Supplementary file 4 — Supplementary Information 4. [file 41598_2022_13394_MOESM4_ESM.zip › Supplementary Figure S3/Supplementary_Figure_S3_146.tif]

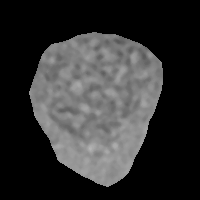

Supplement: Supplementary file 4 — Supplementary Information 4. [file 41598_2022_13394_MOESM4_ESM.zip › Supplementary Figure S3/Supplementary_Figure_S3_147.tif]

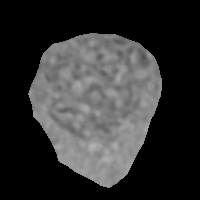

Supplement: Supplementary file 4 — Supplementary Information 4. [file 41598_2022_13394_MOESM4_ESM.zip › Supplementary Figure S3/Supplementary_Figure_S3_148.tif]

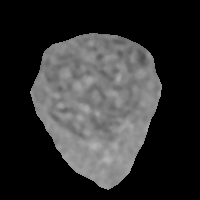

Supplement: Supplementary file 4 — Supplementary Information 4. [file 41598_2022_13394_MOESM4_ESM.zip › Supplementary Figure S3/Supplementary_Figure_S3_149.tif]

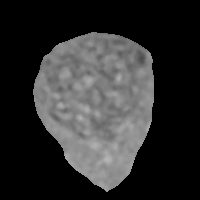

Supplement: Supplementary file 4 — Supplementary Information 4. [file 41598_2022_13394_MOESM4_ESM.zip › Supplementary Figure S3/Supplementary_Figure_S3_150.tif]

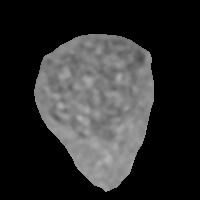

Supplement: Supplementary file 4 — Supplementary Information 4. [file 41598_2022_13394_MOESM4_ESM.zip › Supplementary Figure S3/Supplementary_Figure_S3_151.tif]

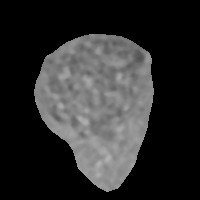

Supplement: Supplementary file 4 — Supplementary Information 4. [file 41598_2022_13394_MOESM4_ESM.zip › Supplementary Figure S3/Supplementary_Figure_S3_152.tif]

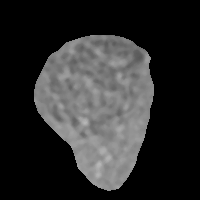

Supplement: Supplementary file 4 — Supplementary Information 4. [file 41598_2022_13394_MOESM4_ESM.zip › Supplementary Figure S3/Supplementary_Figure_S3_153.tif]

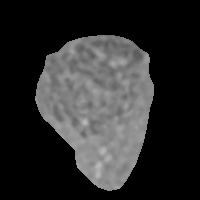

Supplement: Supplementary file 4 — Supplementary Information 4. [file 41598_2022_13394_MOESM4_ESM.zip › Supplementary Figure S3/Supplementary_Figure_S3_154.tif]

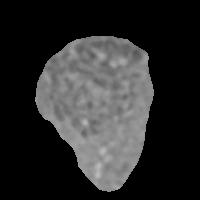

Supplement: Supplementary file 4 — Supplementary Information 4. [file 41598_2022_13394_MOESM4_ESM.zip › Supplementary Figure S3/Supplementary_Figure_S3_155.tif]

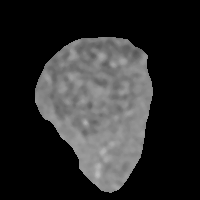

Supplement: Supplementary file 4 — Supplementary Information 4. [file 41598_2022_13394_MOESM4_ESM.zip › Supplementary Figure S3/Supplementary_Figure_S3_156.tif]

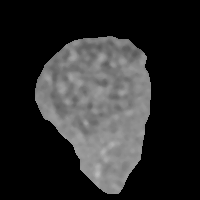

Supplement: Supplementary file 4 — Supplementary Information 4. [file 41598_2022_13394_MOESM4_ESM.zip › Supplementary Figure S3/Supplementary_Figure_S3_157.tif]

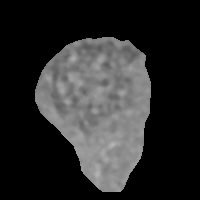

Supplement: Supplementary file 4 — Supplementary Information 4. [file 41598_2022_13394_MOESM4_ESM.zip › Supplementary Figure S3/Supplementary_Figure_S3_158.tif]

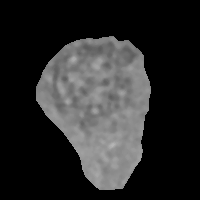

Supplement: Supplementary file 4 — Supplementary Information 4. [file 41598_2022_13394_MOESM4_ESM.zip › Supplementary Figure S3/Supplementary_Figure_S3_159.tif]

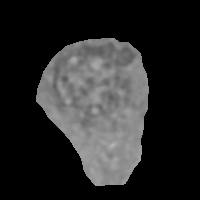

Supplement: Supplementary file 4 — Supplementary Information 4. [file 41598_2022_13394_MOESM4_ESM.zip › Supplementary Figure S3/Supplementary_Figure_S3_160.tif]

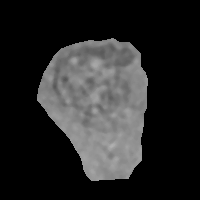

Supplement: Supplementary file 4 — Supplementary Information 4. [file 41598_2022_13394_MOESM4_ESM.zip › Supplementary Figure S3/Supplementary_Figure_S3_161.tif]

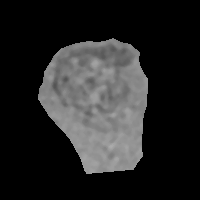

Supplement: Supplementary file 4 — Supplementary Information 4. [file 41598_2022_13394_MOESM4_ESM.zip › Supplementary Figure S3/Supplementary_Figure_S3_162.tif]

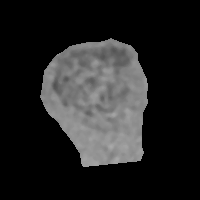

Supplement: Supplementary file 4 — Supplementary Information 4. [file 41598_2022_13394_MOESM4_ESM.zip › Supplementary Figure S3/Supplementary_Figure_S3_163.tif]

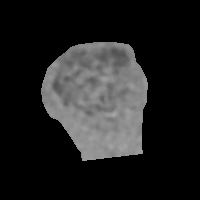

Supplement: Supplementary file 4 — Supplementary Information 4. [file 41598_2022_13394_MOESM4_ESM.zip › Supplementary Figure S3/Supplementary_Figure_S3_164.tif]

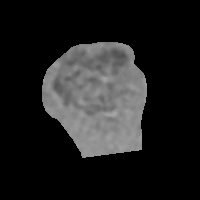

Supplement: Supplementary file 4 — Supplementary Information 4. [file 41598_2022_13394_MOESM4_ESM.zip › Supplementary Figure S3/Supplementary_Figure_S3_165.tif]

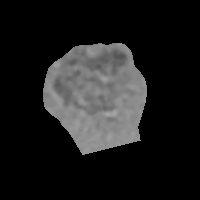

Supplement: Supplementary file 4 — Supplementary Information 4. [file 41598_2022_13394_MOESM4_ESM.zip › Supplementary Figure S3/Supplementary_Figure_S3_166.tif]

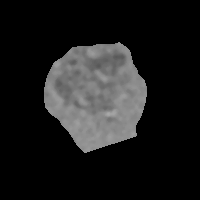

Supplement: Supplementary file 4 — Supplementary Information 4. [file 41598_2022_13394_MOESM4_ESM.zip › Supplementary Figure S3/Supplementary_Figure_S3_167.tif]

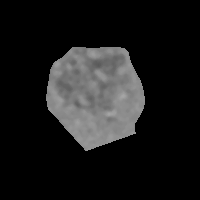

Supplement: Supplementary file 4 — Supplementary Information 4. [file 41598_2022_13394_MOESM4_ESM.zip › Supplementary Figure S3/Supplementary_Figure_S3_168.tif]

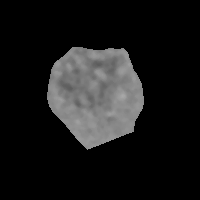

Supplement: Supplementary file 4 — Supplementary Information 4. [file 41598_2022_13394_MOESM4_ESM.zip › Supplementary Figure S3/Supplementary_Figure_S3_169.tif]

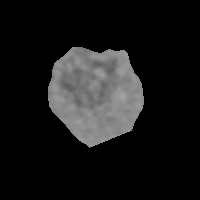

Supplement: Supplementary file 4 — Supplementary Information 4. [file 41598_2022_13394_MOESM4_ESM.zip › Supplementary Figure S3/Supplementary_Figure_S3_170.tif]

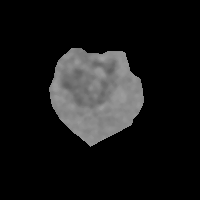

Supplement: Supplementary file 4 — Supplementary Information 4. [file 41598_2022_13394_MOESM4_ESM.zip › Supplementary Figure S3/Supplementary_Figure_S3_171.tif]

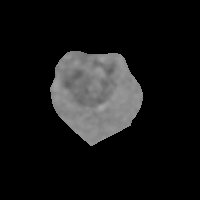

Supplement: Supplementary file 4 — Supplementary Information 4. [file 41598_2022_13394_MOESM4_ESM.zip › Supplementary Figure S3/Supplementary_Figure_S3_172.tif]

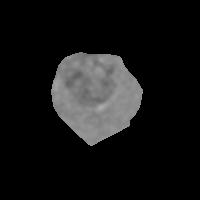

Supplement: Supplementary file 4 — Supplementary Information 4. [file 41598_2022_13394_MOESM4_ESM.zip › Supplementary Figure S3/Supplementary_Figure_S3_173.tif]

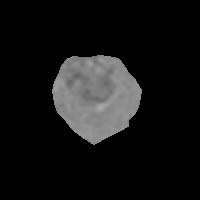

Supplement: Supplementary file 4 — Supplementary Information 4. [file 41598_2022_13394_MOESM4_ESM.zip › Supplementary Figure S3/Supplementary_Figure_S3_174.tif]

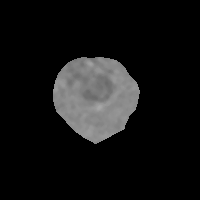

Supplement: Supplementary file 4 — Supplementary Information 4. [file 41598_2022_13394_MOESM4_ESM.zip › Supplementary Figure S3/Supplementary_Figure_S3_175.tif]

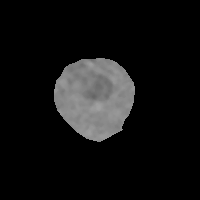

Supplement: Supplementary file 4 — Supplementary Information 4. [file 41598_2022_13394_MOESM4_ESM.zip › Supplementary Figure S3/Supplementary_Figure_S3_176.tif]

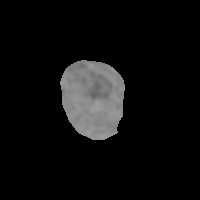

Supplement: Supplementary file 4 — Supplementary Information 4. [file 41598_2022_13394_MOESM4_ESM.zip › Supplementary Figure S3/Supplementary_Figure_S3_177.tif]

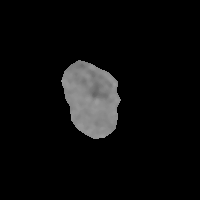

Supplement: Supplementary file 4 — Supplementary Information 4. [file 41598_2022_13394_MOESM4_ESM.zip › Supplementary Figure S3/Supplementary_Figure_S3_178.tif]

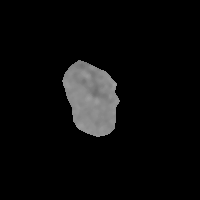

Supplement: Supplementary file 4 — Supplementary Information 4. [file 41598_2022_13394_MOESM4_ESM.zip › Supplementary Figure S3/Supplementary_Figure_S3_179.tif]

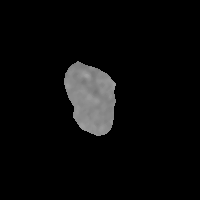

Supplement: Supplementary file 4 — Supplementary Information 4. [file 41598_2022_13394_MOESM4_ESM.zip › Supplementary Figure S3/Supplementary_Figure_S3_180.tif]

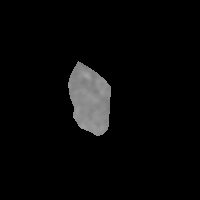

Supplement: Supplementary file 4 — Supplementary Information 4. [file 41598_2022_13394_MOESM4_ESM.zip › Supplementary Figure S3/Supplementary_Figure_S3_181.tif]

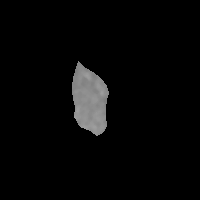

Supplement: Supplementary file 4 — Supplementary Information 4. [file 41598_2022_13394_MOESM4_ESM.zip › Supplementary Figure S3/Supplementary_Figure_S3_182.tif]

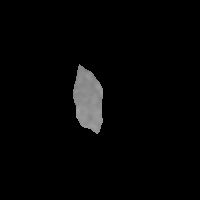

Supplement: Supplementary file 4 — Supplementary Information 4. [file 41598_2022_13394_MOESM4_ESM.zip › Supplementary Figure S3/Supplementary_Figure_S3_183.tif]

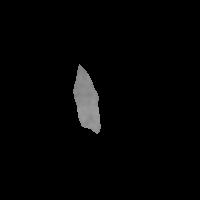

Supplement: Supplementary file 4 — Supplementary Information 4. [file 41598_2022_13394_MOESM4_ESM.zip › Supplementary Figure S3/Supplementary_Figure_S3_184.tif]

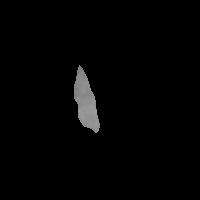

Supplement: Supplementary file 4 — Supplementary Information 4. [file 41598_2022_13394_MOESM4_ESM.zip › Supplementary Figure S3/Supplementary_Figure_S3_185.tif]

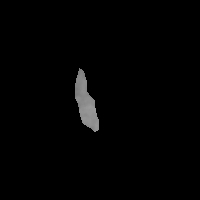

Supplement: Supplementary file 4 — Supplementary Information 4. [file 41598_2022_13394_MOESM4_ESM.zip › Supplementary Figure S3/Supplementary_Figure_S3_186.tif]

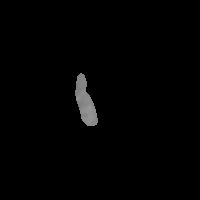

Supplement: Supplementary file 4 — Supplementary Information 4. [file 41598_2022_13394_MOESM4_ESM.zip › Supplementary Figure S3/Supplementary_Figure_S3_187.tif]

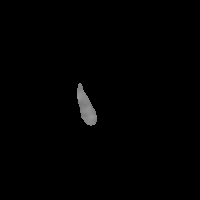

Supplement: Supplementary file 4 — Supplementary Information 4. [file 41598_2022_13394_MOESM4_ESM.zip › Supplementary Figure S3/Supplementary_Figure_S3_188.tif]

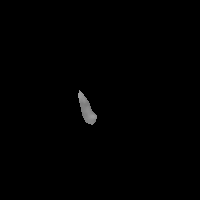

Supplement: Supplementary file 4 — Supplementary Information 4. [file 41598_2022_13394_MOESM4_ESM.zip › Supplementary Figure S3/Supplementary_Figure_S3_189.tif]

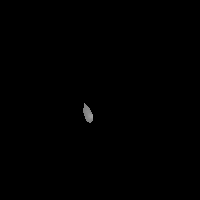

Supplement: Supplementary file 4 — Supplementary Information 4. [file 41598_2022_13394_MOESM4_ESM.zip › Supplementary Figure S3/Supplementary_Figure_S3_190.tif]

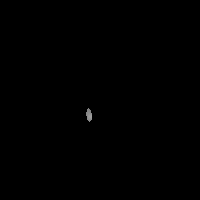

Supplement: Supplementary file 4 — Supplementary Information 4. [file 41598_2022_13394_MOESM4_ESM.zip › Supplementary Figure S3/Supplementary_Figure_S3_191.tif]

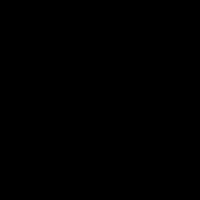

Supplement: Supplementary file 4 — Supplementary Information 4. [file 41598_2022_13394_MOESM4_ESM.zip › Supplementary Figure S3/Supplementary_Figure_S3_192.tif]

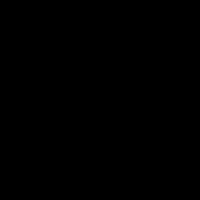

Supplement: Supplementary file 4 — Supplementary Information 4. [file 41598_2022_13394_MOESM4_ESM.zip › Supplementary Figure S3/Supplementary_Figure_S3_193.tif]

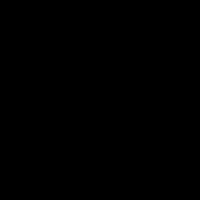

Supplement: Supplementary file 4 — Supplementary Information 4. [file 41598_2022_13394_MOESM4_ESM.zip › Supplementary Figure S3/Supplementary_Figure_S3_194.tif]

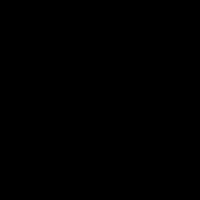

Supplement: Supplementary file 4 — Supplementary Information 4. [file 41598_2022_13394_MOESM4_ESM.zip › Supplementary Figure S3/Supplementary_Figure_S3_195.tif]

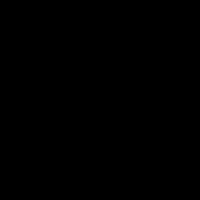

Supplement: Supplementary file 4 — Supplementary Information 4. [file 41598_2022_13394_MOESM4_ESM.zip › Supplementary Figure S3/Supplementary_Figure_S3_196.tif]

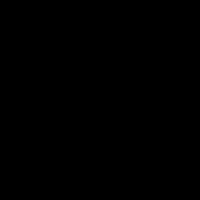

Supplement: Supplementary file 4 — Supplementary Information 4. [file 41598_2022_13394_MOESM4_ESM.zip › Supplementary Figure S3/Supplementary_Figure_S3_197.tif]

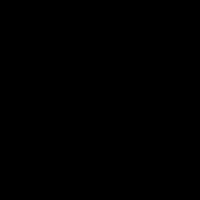

Supplement: Supplementary file 4 — Supplementary Information 4. [file 41598_2022_13394_MOESM4_ESM.zip › Supplementary Figure S3/Supplementary_Figure_S3_198.tif]
