# Supplementary figures and images for: Nondestructive cellular-level 3D observation of mouse kidney using laboratory-based X-ray microscopy with paraffin-mediated contrast enhancement (part 7 of 9)
Source: Sci Rep. 2022 Jun 8;12:9436. doi: 10.1038/s41598-022-13394-9 (PMC9177607; doi:10.1038/s41598-022-13394-9)

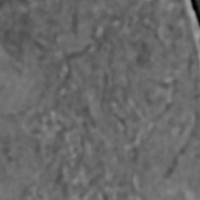

Supplement: Supplementary file 5 — Supplementary Information 5. [file 41598_2022_13394_MOESM5_ESM.zip › Supplementary Figure S4/Supplementary_Figure_S4_199.tif]

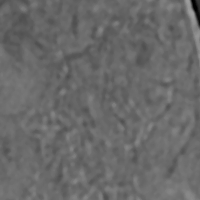

Supplement: Supplementary file 5 — Supplementary Information 5. [file 41598_2022_13394_MOESM5_ESM.zip › Supplementary Figure S4/Supplementary_Figure_S4_200.tif]

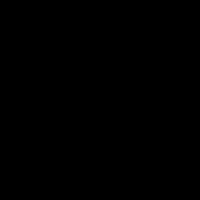

Supplement: Supplementary file 6 — Supplementary Information 6. [file 41598_2022_13394_MOESM6_ESM.zip › Supplementary Figure S5/Supplementary_Figure_S5_001.tif]

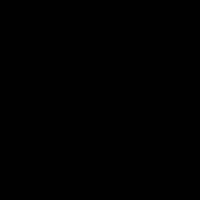

Supplement: Supplementary file 6 — Supplementary Information 6. [file 41598_2022_13394_MOESM6_ESM.zip › Supplementary Figure S5/Supplementary_Figure_S5_002.tif]

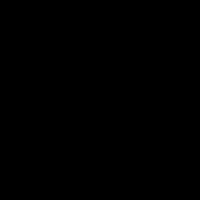

Supplement: Supplementary file 6 — Supplementary Information 6. [file 41598_2022_13394_MOESM6_ESM.zip › Supplementary Figure S5/Supplementary_Figure_S5_003.tif]

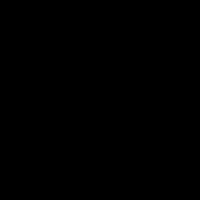

Supplement: Supplementary file 6 — Supplementary Information 6. [file 41598_2022_13394_MOESM6_ESM.zip › Supplementary Figure S5/Supplementary_Figure_S5_004.tif]

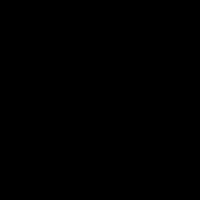

Supplement: Supplementary file 6 — Supplementary Information 6. [file 41598_2022_13394_MOESM6_ESM.zip › Supplementary Figure S5/Supplementary_Figure_S5_005.tif]

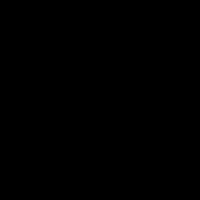

Supplement: Supplementary file 6 — Supplementary Information 6. [file 41598_2022_13394_MOESM6_ESM.zip › Supplementary Figure S5/Supplementary_Figure_S5_006.tif]

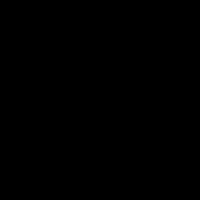

Supplement: Supplementary file 6 — Supplementary Information 6. [file 41598_2022_13394_MOESM6_ESM.zip › Supplementary Figure S5/Supplementary_Figure_S5_007.tif]

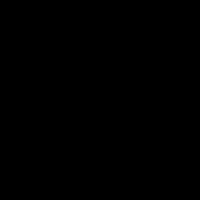

Supplement: Supplementary file 6 — Supplementary Information 6. [file 41598_2022_13394_MOESM6_ESM.zip › Supplementary Figure S5/Supplementary_Figure_S5_008.tif]

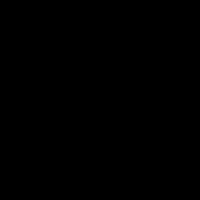

Supplement: Supplementary file 6 — Supplementary Information 6. [file 41598_2022_13394_MOESM6_ESM.zip › Supplementary Figure S5/Supplementary_Figure_S5_009.tif]

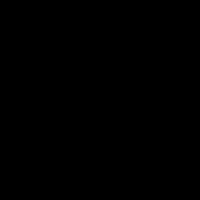

Supplement: Supplementary file 6 — Supplementary Information 6. [file 41598_2022_13394_MOESM6_ESM.zip › Supplementary Figure S5/Supplementary_Figure_S5_010.tif]

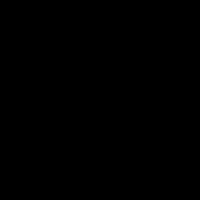

Supplement: Supplementary file 6 — Supplementary Information 6. [file 41598_2022_13394_MOESM6_ESM.zip › Supplementary Figure S5/Supplementary_Figure_S5_011.tif]

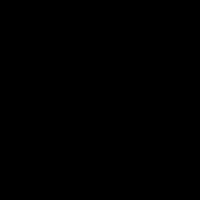

Supplement: Supplementary file 6 — Supplementary Information 6. [file 41598_2022_13394_MOESM6_ESM.zip › Supplementary Figure S5/Supplementary_Figure_S5_012.tif]

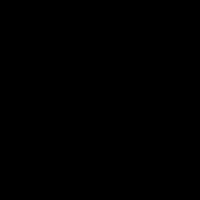

Supplement: Supplementary file 6 — Supplementary Information 6. [file 41598_2022_13394_MOESM6_ESM.zip › Supplementary Figure S5/Supplementary_Figure_S5_013.tif]

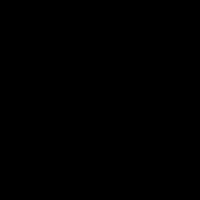

Supplement: Supplementary file 6 — Supplementary Information 6. [file 41598_2022_13394_MOESM6_ESM.zip › Supplementary Figure S5/Supplementary_Figure_S5_014.tif]

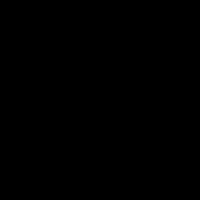

Supplement: Supplementary file 6 — Supplementary Information 6. [file 41598_2022_13394_MOESM6_ESM.zip › Supplementary Figure S5/Supplementary_Figure_S5_015.tif]

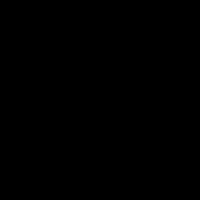

Supplement: Supplementary file 6 — Supplementary Information 6. [file 41598_2022_13394_MOESM6_ESM.zip › Supplementary Figure S5/Supplementary_Figure_S5_016.tif]

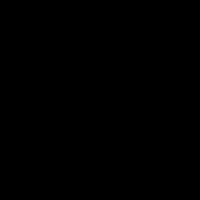

Supplement: Supplementary file 6 — Supplementary Information 6. [file 41598_2022_13394_MOESM6_ESM.zip › Supplementary Figure S5/Supplementary_Figure_S5_017.tif]

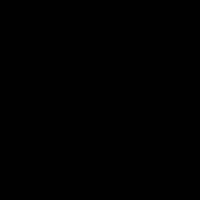

Supplement: Supplementary file 6 — Supplementary Information 6. [file 41598_2022_13394_MOESM6_ESM.zip › Supplementary Figure S5/Supplementary_Figure_S5_018.tif]

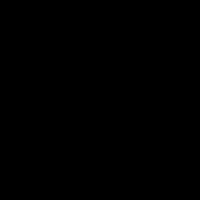

Supplement: Supplementary file 6 — Supplementary Information 6. [file 41598_2022_13394_MOESM6_ESM.zip › Supplementary Figure S5/Supplementary_Figure_S5_019.tif]

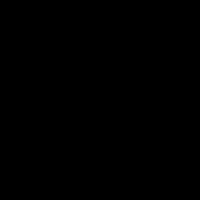

Supplement: Supplementary file 6 — Supplementary Information 6. [file 41598_2022_13394_MOESM6_ESM.zip › Supplementary Figure S5/Supplementary_Figure_S5_020.tif]

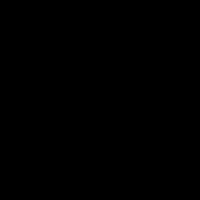

Supplement: Supplementary file 6 — Supplementary Information 6. [file 41598_2022_13394_MOESM6_ESM.zip › Supplementary Figure S5/Supplementary_Figure_S5_021.tif]

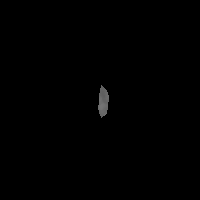

Supplement: Supplementary file 6 — Supplementary Information 6. [file 41598_2022_13394_MOESM6_ESM.zip › Supplementary Figure S5/Supplementary_Figure_S5_022.tif]

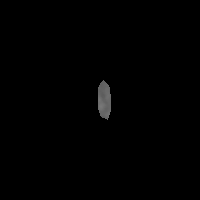

Supplement: Supplementary file 6 — Supplementary Information 6. [file 41598_2022_13394_MOESM6_ESM.zip › Supplementary Figure S5/Supplementary_Figure_S5_023.tif]

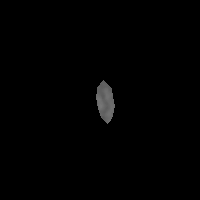

Supplement: Supplementary file 6 — Supplementary Information 6. [file 41598_2022_13394_MOESM6_ESM.zip › Supplementary Figure S5/Supplementary_Figure_S5_024.tif]

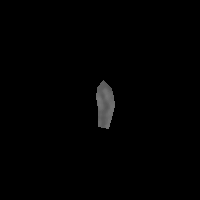

Supplement: Supplementary file 6 — Supplementary Information 6. [file 41598_2022_13394_MOESM6_ESM.zip › Supplementary Figure S5/Supplementary_Figure_S5_025.tif]

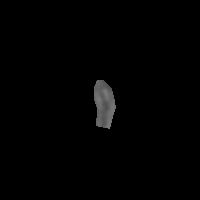

Supplement: Supplementary file 6 — Supplementary Information 6. [file 41598_2022_13394_MOESM6_ESM.zip › Supplementary Figure S5/Supplementary_Figure_S5_026.tif]

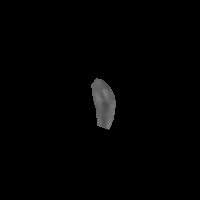

Supplement: Supplementary file 6 — Supplementary Information 6. [file 41598_2022_13394_MOESM6_ESM.zip › Supplementary Figure S5/Supplementary_Figure_S5_027.tif]

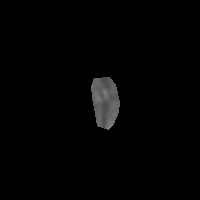

Supplement: Supplementary file 6 — Supplementary Information 6. [file 41598_2022_13394_MOESM6_ESM.zip › Supplementary Figure S5/Supplementary_Figure_S5_028.tif]

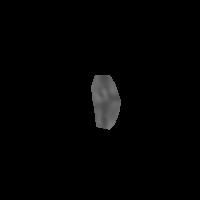

Supplement: Supplementary file 6 — Supplementary Information 6. [file 41598_2022_13394_MOESM6_ESM.zip › Supplementary Figure S5/Supplementary_Figure_S5_029.tif]

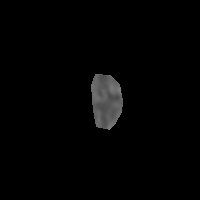

Supplement: Supplementary file 6 — Supplementary Information 6. [file 41598_2022_13394_MOESM6_ESM.zip › Supplementary Figure S5/Supplementary_Figure_S5_030.tif]

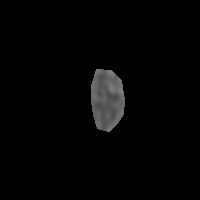

Supplement: Supplementary file 6 — Supplementary Information 6. [file 41598_2022_13394_MOESM6_ESM.zip › Supplementary Figure S5/Supplementary_Figure_S5_031.tif]

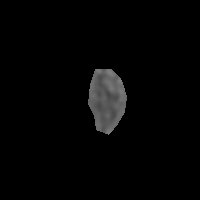

Supplement: Supplementary file 6 — Supplementary Information 6. [file 41598_2022_13394_MOESM6_ESM.zip › Supplementary Figure S5/Supplementary_Figure_S5_032.tif]

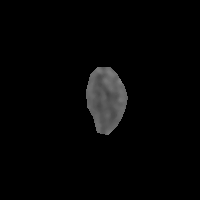

Supplement: Supplementary file 6 — Supplementary Information 6. [file 41598_2022_13394_MOESM6_ESM.zip › Supplementary Figure S5/Supplementary_Figure_S5_033.tif]

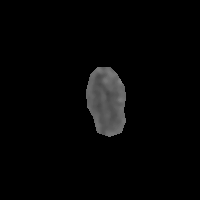

Supplement: Supplementary file 6 — Supplementary Information 6. [file 41598_2022_13394_MOESM6_ESM.zip › Supplementary Figure S5/Supplementary_Figure_S5_034.tif]

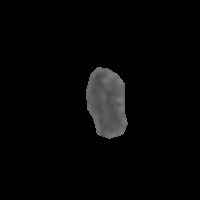

Supplement: Supplementary file 6 — Supplementary Information 6. [file 41598_2022_13394_MOESM6_ESM.zip › Supplementary Figure S5/Supplementary_Figure_S5_035.tif]

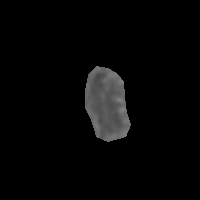

Supplement: Supplementary file 6 — Supplementary Information 6. [file 41598_2022_13394_MOESM6_ESM.zip › Supplementary Figure S5/Supplementary_Figure_S5_036.tif]

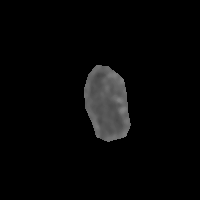

Supplement: Supplementary file 6 — Supplementary Information 6. [file 41598_2022_13394_MOESM6_ESM.zip › Supplementary Figure S5/Supplementary_Figure_S5_037.tif]

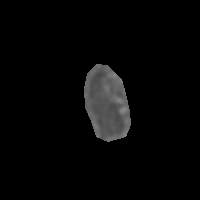

Supplement: Supplementary file 6 — Supplementary Information 6. [file 41598_2022_13394_MOESM6_ESM.zip › Supplementary Figure S5/Supplementary_Figure_S5_038.tif]

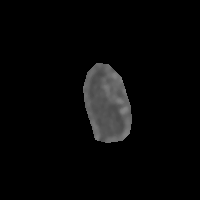

Supplement: Supplementary file 6 — Supplementary Information 6. [file 41598_2022_13394_MOESM6_ESM.zip › Supplementary Figure S5/Supplementary_Figure_S5_039.tif]

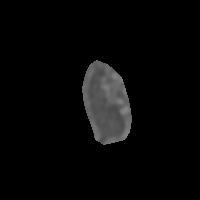

Supplement: Supplementary file 6 — Supplementary Information 6. [file 41598_2022_13394_MOESM6_ESM.zip › Supplementary Figure S5/Supplementary_Figure_S5_040.tif]

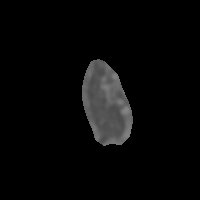

Supplement: Supplementary file 6 — Supplementary Information 6. [file 41598_2022_13394_MOESM6_ESM.zip › Supplementary Figure S5/Supplementary_Figure_S5_041.tif]

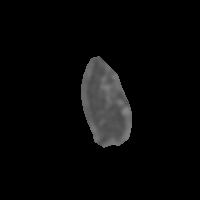

Supplement: Supplementary file 6 — Supplementary Information 6. [file 41598_2022_13394_MOESM6_ESM.zip › Supplementary Figure S5/Supplementary_Figure_S5_042.tif]

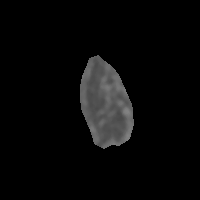

Supplement: Supplementary file 6 — Supplementary Information 6. [file 41598_2022_13394_MOESM6_ESM.zip › Supplementary Figure S5/Supplementary_Figure_S5_043.tif]

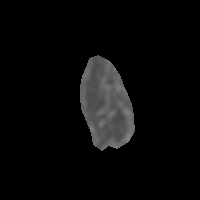

Supplement: Supplementary file 6 — Supplementary Information 6. [file 41598_2022_13394_MOESM6_ESM.zip › Supplementary Figure S5/Supplementary_Figure_S5_044.tif]

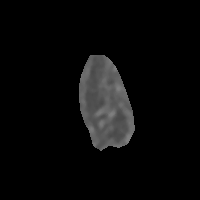

Supplement: Supplementary file 6 — Supplementary Information 6. [file 41598_2022_13394_MOESM6_ESM.zip › Supplementary Figure S5/Supplementary_Figure_S5_045.tif]

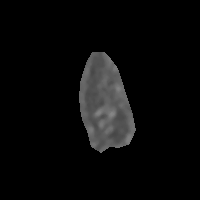

Supplement: Supplementary file 6 — Supplementary Information 6. [file 41598_2022_13394_MOESM6_ESM.zip › Supplementary Figure S5/Supplementary_Figure_S5_046.tif]

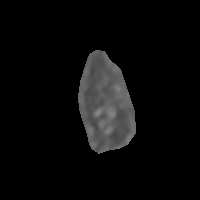

Supplement: Supplementary file 6 — Supplementary Information 6. [file 41598_2022_13394_MOESM6_ESM.zip › Supplementary Figure S5/Supplementary_Figure_S5_047.tif]

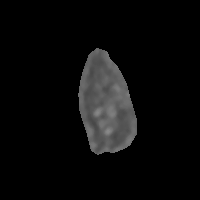

Supplement: Supplementary file 6 — Supplementary Information 6. [file 41598_2022_13394_MOESM6_ESM.zip › Supplementary Figure S5/Supplementary_Figure_S5_048.tif]

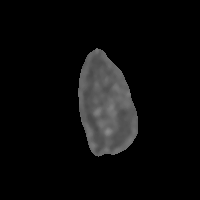

Supplement: Supplementary file 6 — Supplementary Information 6. [file 41598_2022_13394_MOESM6_ESM.zip › Supplementary Figure S5/Supplementary_Figure_S5_049.tif]

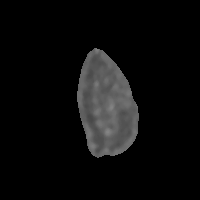

Supplement: Supplementary file 6 — Supplementary Information 6. [file 41598_2022_13394_MOESM6_ESM.zip › Supplementary Figure S5/Supplementary_Figure_S5_050.tif]

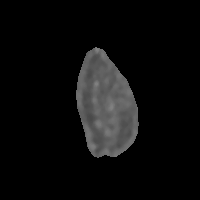

Supplement: Supplementary file 6 — Supplementary Information 6. [file 41598_2022_13394_MOESM6_ESM.zip › Supplementary Figure S5/Supplementary_Figure_S5_051.tif]

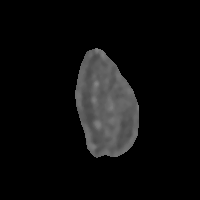

Supplement: Supplementary file 6 — Supplementary Information 6. [file 41598_2022_13394_MOESM6_ESM.zip › Supplementary Figure S5/Supplementary_Figure_S5_052.tif]

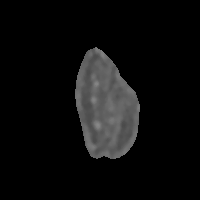

Supplement: Supplementary file 6 — Supplementary Information 6. [file 41598_2022_13394_MOESM6_ESM.zip › Supplementary Figure S5/Supplementary_Figure_S5_053.tif]

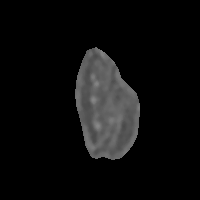

Supplement: Supplementary file 6 — Supplementary Information 6. [file 41598_2022_13394_MOESM6_ESM.zip › Supplementary Figure S5/Supplementary_Figure_S5_054.tif]

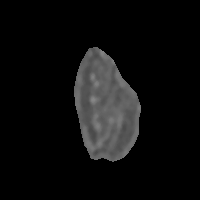

Supplement: Supplementary file 6 — Supplementary Information 6. [file 41598_2022_13394_MOESM6_ESM.zip › Supplementary Figure S5/Supplementary_Figure_S5_055.tif]

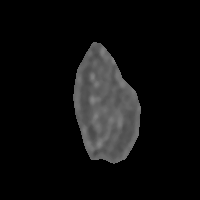

Supplement: Supplementary file 6 — Supplementary Information 6. [file 41598_2022_13394_MOESM6_ESM.zip › Supplementary Figure S5/Supplementary_Figure_S5_056.tif]

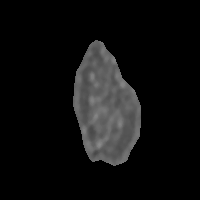

Supplement: Supplementary file 6 — Supplementary Information 6. [file 41598_2022_13394_MOESM6_ESM.zip › Supplementary Figure S5/Supplementary_Figure_S5_057.tif]

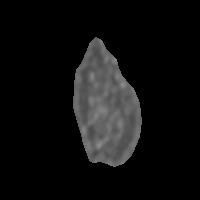

Supplement: Supplementary file 6 — Supplementary Information 6. [file 41598_2022_13394_MOESM6_ESM.zip › Supplementary Figure S5/Supplementary_Figure_S5_058.tif]

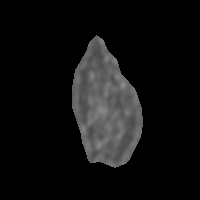

Supplement: Supplementary file 6 — Supplementary Information 6. [file 41598_2022_13394_MOESM6_ESM.zip › Supplementary Figure S5/Supplementary_Figure_S5_059.tif]

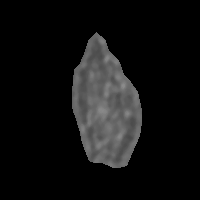

Supplement: Supplementary file 6 — Supplementary Information 6. [file 41598_2022_13394_MOESM6_ESM.zip › Supplementary Figure S5/Supplementary_Figure_S5_060.tif]

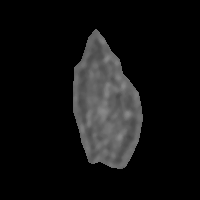

Supplement: Supplementary file 6 — Supplementary Information 6. [file 41598_2022_13394_MOESM6_ESM.zip › Supplementary Figure S5/Supplementary_Figure_S5_061.tif]

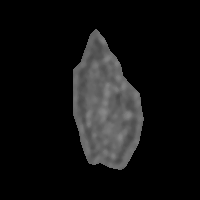

Supplement: Supplementary file 6 — Supplementary Information 6. [file 41598_2022_13394_MOESM6_ESM.zip › Supplementary Figure S5/Supplementary_Figure_S5_062.tif]

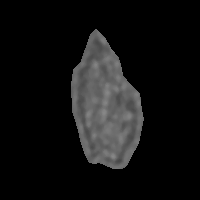

Supplement: Supplementary file 6 — Supplementary Information 6. [file 41598_2022_13394_MOESM6_ESM.zip › Supplementary Figure S5/Supplementary_Figure_S5_063.tif]

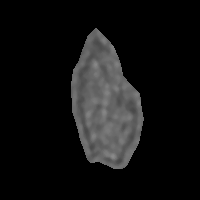

Supplement: Supplementary file 6 — Supplementary Information 6. [file 41598_2022_13394_MOESM6_ESM.zip › Supplementary Figure S5/Supplementary_Figure_S5_064.tif]

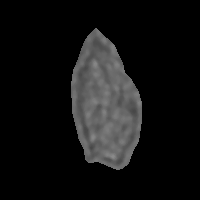

Supplement: Supplementary file 6 — Supplementary Information 6. [file 41598_2022_13394_MOESM6_ESM.zip › Supplementary Figure S5/Supplementary_Figure_S5_065.tif]

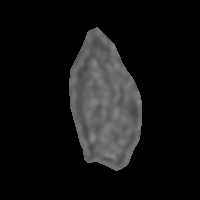

Supplement: Supplementary file 6 — Supplementary Information 6. [file 41598_2022_13394_MOESM6_ESM.zip › Supplementary Figure S5/Supplementary_Figure_S5_066.tif]

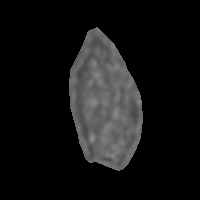

Supplement: Supplementary file 6 — Supplementary Information 6. [file 41598_2022_13394_MOESM6_ESM.zip › Supplementary Figure S5/Supplementary_Figure_S5_067.tif]

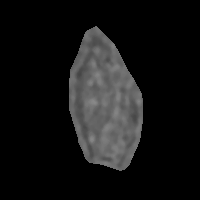

Supplement: Supplementary file 6 — Supplementary Information 6. [file 41598_2022_13394_MOESM6_ESM.zip › Supplementary Figure S5/Supplementary_Figure_S5_068.tif]

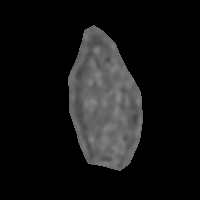

Supplement: Supplementary file 6 — Supplementary Information 6. [file 41598_2022_13394_MOESM6_ESM.zip › Supplementary Figure S5/Supplementary_Figure_S5_069.tif]

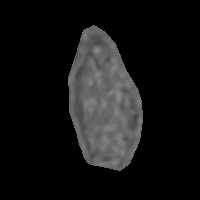

Supplement: Supplementary file 6 — Supplementary Information 6. [file 41598_2022_13394_MOESM6_ESM.zip › Supplementary Figure S5/Supplementary_Figure_S5_070.tif]

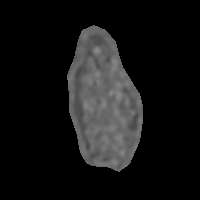

Supplement: Supplementary file 6 — Supplementary Information 6. [file 41598_2022_13394_MOESM6_ESM.zip › Supplementary Figure S5/Supplementary_Figure_S5_071.tif]

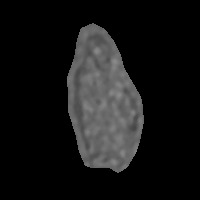

Supplement: Supplementary file 6 — Supplementary Information 6. [file 41598_2022_13394_MOESM6_ESM.zip › Supplementary Figure S5/Supplementary_Figure_S5_072.tif]

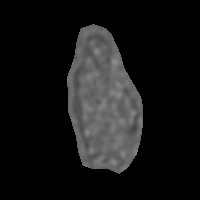

Supplement: Supplementary file 6 — Supplementary Information 6. [file 41598_2022_13394_MOESM6_ESM.zip › Supplementary Figure S5/Supplementary_Figure_S5_073.tif]

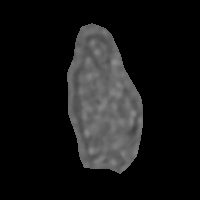

Supplement: Supplementary file 6 — Supplementary Information 6. [file 41598_2022_13394_MOESM6_ESM.zip › Supplementary Figure S5/Supplementary_Figure_S5_074.tif]

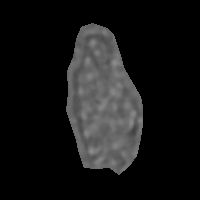

Supplement: Supplementary file 6 — Supplementary Information 6. [file 41598_2022_13394_MOESM6_ESM.zip › Supplementary Figure S5/Supplementary_Figure_S5_075.tif]

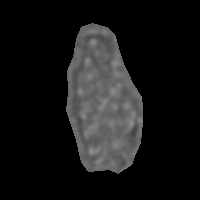

Supplement: Supplementary file 6 — Supplementary Information 6. [file 41598_2022_13394_MOESM6_ESM.zip › Supplementary Figure S5/Supplementary_Figure_S5_076.tif]

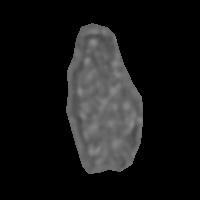

Supplement: Supplementary file 6 — Supplementary Information 6. [file 41598_2022_13394_MOESM6_ESM.zip › Supplementary Figure S5/Supplementary_Figure_S5_077.tif]

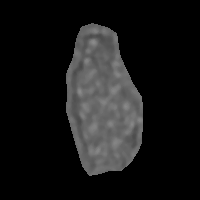

Supplement: Supplementary file 6 — Supplementary Information 6. [file 41598_2022_13394_MOESM6_ESM.zip › Supplementary Figure S5/Supplementary_Figure_S5_078.tif]

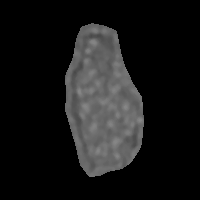

Supplement: Supplementary file 6 — Supplementary Information 6. [file 41598_2022_13394_MOESM6_ESM.zip › Supplementary Figure S5/Supplementary_Figure_S5_079.tif]

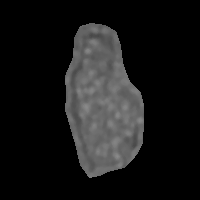

Supplement: Supplementary file 6 — Supplementary Information 6. [file 41598_2022_13394_MOESM6_ESM.zip › Supplementary Figure S5/Supplementary_Figure_S5_080.tif]

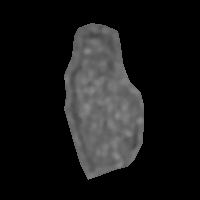

Supplement: Supplementary file 6 — Supplementary Information 6. [file 41598_2022_13394_MOESM6_ESM.zip › Supplementary Figure S5/Supplementary_Figure_S5_081.tif]

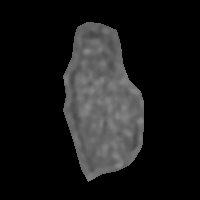

Supplement: Supplementary file 6 — Supplementary Information 6. [file 41598_2022_13394_MOESM6_ESM.zip › Supplementary Figure S5/Supplementary_Figure_S5_082.tif]

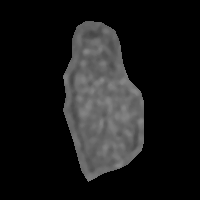

Supplement: Supplementary file 6 — Supplementary Information 6. [file 41598_2022_13394_MOESM6_ESM.zip › Supplementary Figure S5/Supplementary_Figure_S5_083.tif]

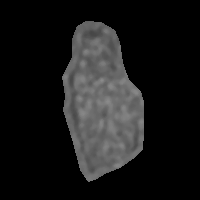

Supplement: Supplementary file 6 — Supplementary Information 6. [file 41598_2022_13394_MOESM6_ESM.zip › Supplementary Figure S5/Supplementary_Figure_S5_084.tif]

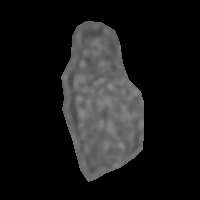

Supplement: Supplementary file 6 — Supplementary Information 6. [file 41598_2022_13394_MOESM6_ESM.zip › Supplementary Figure S5/Supplementary_Figure_S5_085.tif]

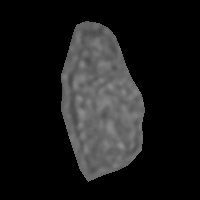

Supplement: Supplementary file 6 — Supplementary Information 6. [file 41598_2022_13394_MOESM6_ESM.zip › Supplementary Figure S5/Supplementary_Figure_S5_086.tif]

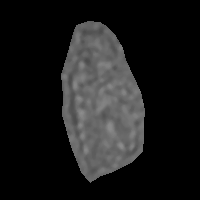

Supplement: Supplementary file 6 — Supplementary Information 6. [file 41598_2022_13394_MOESM6_ESM.zip › Supplementary Figure S5/Supplementary_Figure_S5_087.tif]

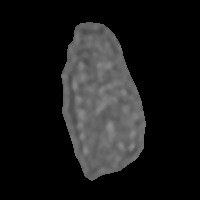

Supplement: Supplementary file 6 — Supplementary Information 6. [file 41598_2022_13394_MOESM6_ESM.zip › Supplementary Figure S5/Supplementary_Figure_S5_088.tif]

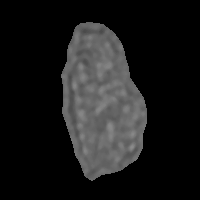

Supplement: Supplementary file 6 — Supplementary Information 6. [file 41598_2022_13394_MOESM6_ESM.zip › Supplementary Figure S5/Supplementary_Figure_S5_089.tif]

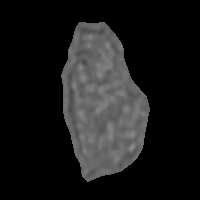

Supplement: Supplementary file 6 — Supplementary Information 6. [file 41598_2022_13394_MOESM6_ESM.zip › Supplementary Figure S5/Supplementary_Figure_S5_090.tif]

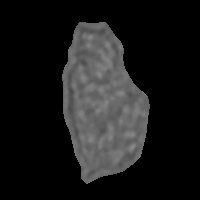

Supplement: Supplementary file 6 — Supplementary Information 6. [file 41598_2022_13394_MOESM6_ESM.zip › Supplementary Figure S5/Supplementary_Figure_S5_091.tif]

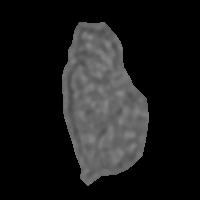

Supplement: Supplementary file 6 — Supplementary Information 6. [file 41598_2022_13394_MOESM6_ESM.zip › Supplementary Figure S5/Supplementary_Figure_S5_092.tif]

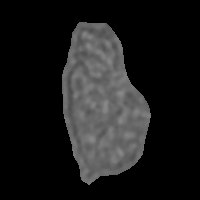

Supplement: Supplementary file 6 — Supplementary Information 6. [file 41598_2022_13394_MOESM6_ESM.zip › Supplementary Figure S5/Supplementary_Figure_S5_093.tif]

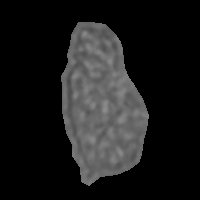

Supplement: Supplementary file 6 — Supplementary Information 6. [file 41598_2022_13394_MOESM6_ESM.zip › Supplementary Figure S5/Supplementary_Figure_S5_094.tif]

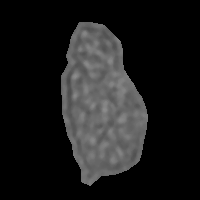

Supplement: Supplementary file 6 — Supplementary Information 6. [file 41598_2022_13394_MOESM6_ESM.zip › Supplementary Figure S5/Supplementary_Figure_S5_095.tif]

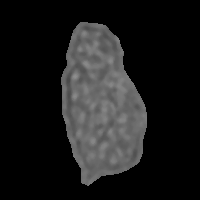

Supplement: Supplementary file 6 — Supplementary Information 6. [file 41598_2022_13394_MOESM6_ESM.zip › Supplementary Figure S5/Supplementary_Figure_S5_096.tif]

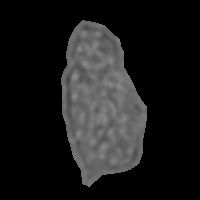

Supplement: Supplementary file 6 — Supplementary Information 6. [file 41598_2022_13394_MOESM6_ESM.zip › Supplementary Figure S5/Supplementary_Figure_S5_097.tif]

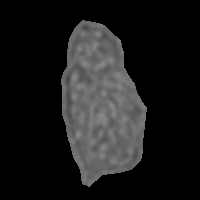

Supplement: Supplementary file 6 — Supplementary Information 6. [file 41598_2022_13394_MOESM6_ESM.zip › Supplementary Figure S5/Supplementary_Figure_S5_098.tif]
