# Supplementary figures and images for: Nondestructive cellular-level 3D observation of mouse kidney using laboratory-based X-ray microscopy with paraffin-mediated contrast enhancement (part 6 of 9)
Source: Sci Rep. 2022 Jun 8;12:9436. doi: 10.1038/s41598-022-13394-9 (PMC9177607; doi:10.1038/s41598-022-13394-9)

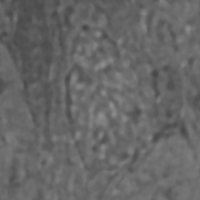

Supplement: Supplementary file 5 — Supplementary Information 5. [file 41598_2022_13394_MOESM5_ESM.zip › Supplementary Figure S4/Supplementary_Figure_S4_099.tif]

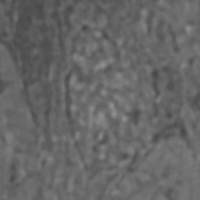

Supplement: Supplementary file 5 — Supplementary Information 5. [file 41598_2022_13394_MOESM5_ESM.zip › Supplementary Figure S4/Supplementary_Figure_S4_100.tif]

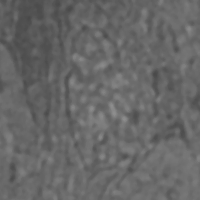

Supplement: Supplementary file 5 — Supplementary Information 5. [file 41598_2022_13394_MOESM5_ESM.zip › Supplementary Figure S4/Supplementary_Figure_S4_101.tif]

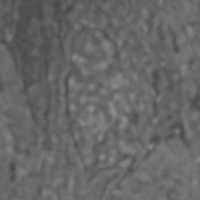

Supplement: Supplementary file 5 — Supplementary Information 5. [file 41598_2022_13394_MOESM5_ESM.zip › Supplementary Figure S4/Supplementary_Figure_S4_102.tif]

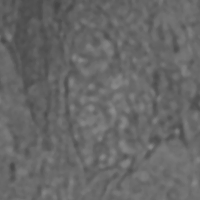

Supplement: Supplementary file 5 — Supplementary Information 5. [file 41598_2022_13394_MOESM5_ESM.zip › Supplementary Figure S4/Supplementary_Figure_S4_103.tif]

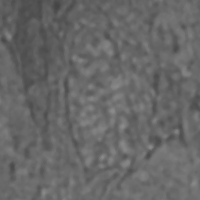

Supplement: Supplementary file 5 — Supplementary Information 5. [file 41598_2022_13394_MOESM5_ESM.zip › Supplementary Figure S4/Supplementary_Figure_S4_104.tif]

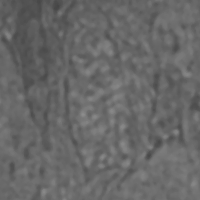

Supplement: Supplementary file 5 — Supplementary Information 5. [file 41598_2022_13394_MOESM5_ESM.zip › Supplementary Figure S4/Supplementary_Figure_S4_105.tif]

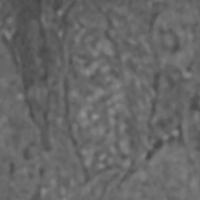

Supplement: Supplementary file 5 — Supplementary Information 5. [file 41598_2022_13394_MOESM5_ESM.zip › Supplementary Figure S4/Supplementary_Figure_S4_106.tif]

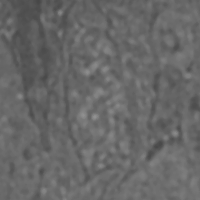

Supplement: Supplementary file 5 — Supplementary Information 5. [file 41598_2022_13394_MOESM5_ESM.zip › Supplementary Figure S4/Supplementary_Figure_S4_107.tif]

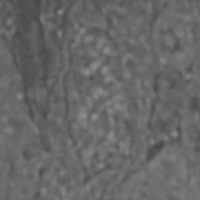

Supplement: Supplementary file 5 — Supplementary Information 5. [file 41598_2022_13394_MOESM5_ESM.zip › Supplementary Figure S4/Supplementary_Figure_S4_108.tif]

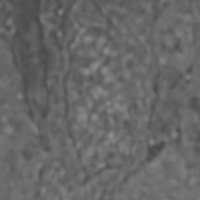

Supplement: Supplementary file 5 — Supplementary Information 5. [file 41598_2022_13394_MOESM5_ESM.zip › Supplementary Figure S4/Supplementary_Figure_S4_109.tif]

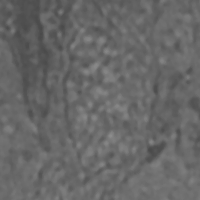

Supplement: Supplementary file 5 — Supplementary Information 5. [file 41598_2022_13394_MOESM5_ESM.zip › Supplementary Figure S4/Supplementary_Figure_S4_110.tif]

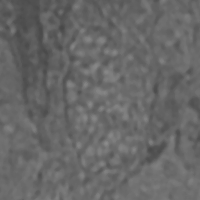

Supplement: Supplementary file 5 — Supplementary Information 5. [file 41598_2022_13394_MOESM5_ESM.zip › Supplementary Figure S4/Supplementary_Figure_S4_111.tif]

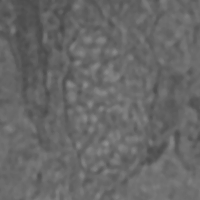

Supplement: Supplementary file 5 — Supplementary Information 5. [file 41598_2022_13394_MOESM5_ESM.zip › Supplementary Figure S4/Supplementary_Figure_S4_112.tif]

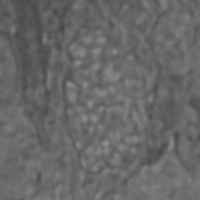

Supplement: Supplementary file 5 — Supplementary Information 5. [file 41598_2022_13394_MOESM5_ESM.zip › Supplementary Figure S4/Supplementary_Figure_S4_113.tif]

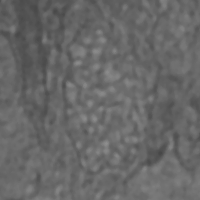

Supplement: Supplementary file 5 — Supplementary Information 5. [file 41598_2022_13394_MOESM5_ESM.zip › Supplementary Figure S4/Supplementary_Figure_S4_114.tif]

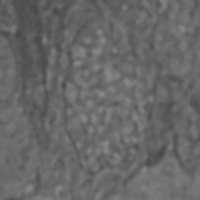

Supplement: Supplementary file 5 — Supplementary Information 5. [file 41598_2022_13394_MOESM5_ESM.zip › Supplementary Figure S4/Supplementary_Figure_S4_115.tif]

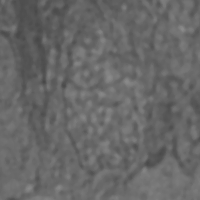

Supplement: Supplementary file 5 — Supplementary Information 5. [file 41598_2022_13394_MOESM5_ESM.zip › Supplementary Figure S4/Supplementary_Figure_S4_116.tif]

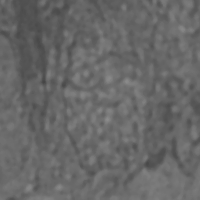

Supplement: Supplementary file 5 — Supplementary Information 5. [file 41598_2022_13394_MOESM5_ESM.zip › Supplementary Figure S4/Supplementary_Figure_S4_117.tif]

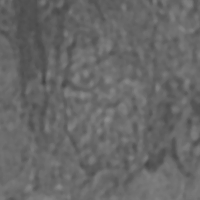

Supplement: Supplementary file 5 — Supplementary Information 5. [file 41598_2022_13394_MOESM5_ESM.zip › Supplementary Figure S4/Supplementary_Figure_S4_118.tif]

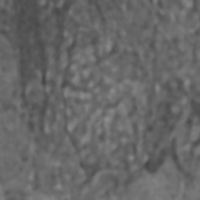

Supplement: Supplementary file 5 — Supplementary Information 5. [file 41598_2022_13394_MOESM5_ESM.zip › Supplementary Figure S4/Supplementary_Figure_S4_119.tif]

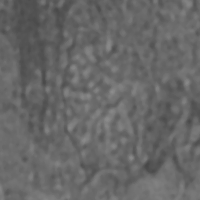

Supplement: Supplementary file 5 — Supplementary Information 5. [file 41598_2022_13394_MOESM5_ESM.zip › Supplementary Figure S4/Supplementary_Figure_S4_120.tif]

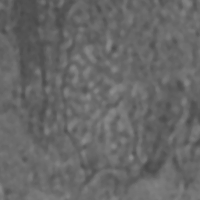

Supplement: Supplementary file 5 — Supplementary Information 5. [file 41598_2022_13394_MOESM5_ESM.zip › Supplementary Figure S4/Supplementary_Figure_S4_121.tif]

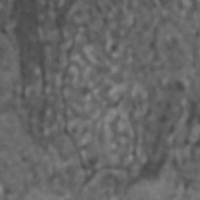

Supplement: Supplementary file 5 — Supplementary Information 5. [file 41598_2022_13394_MOESM5_ESM.zip › Supplementary Figure S4/Supplementary_Figure_S4_122.tif]

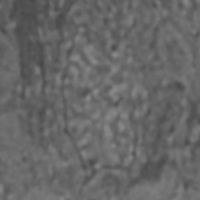

Supplement: Supplementary file 5 — Supplementary Information 5. [file 41598_2022_13394_MOESM5_ESM.zip › Supplementary Figure S4/Supplementary_Figure_S4_123.tif]

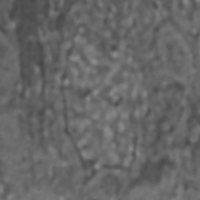

Supplement: Supplementary file 5 — Supplementary Information 5. [file 41598_2022_13394_MOESM5_ESM.zip › Supplementary Figure S4/Supplementary_Figure_S4_124.tif]

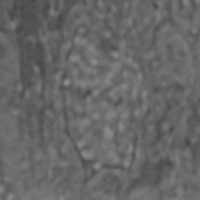

Supplement: Supplementary file 5 — Supplementary Information 5. [file 41598_2022_13394_MOESM5_ESM.zip › Supplementary Figure S4/Supplementary_Figure_S4_125.tif]

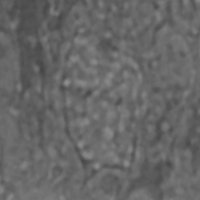

Supplement: Supplementary file 5 — Supplementary Information 5. [file 41598_2022_13394_MOESM5_ESM.zip › Supplementary Figure S4/Supplementary_Figure_S4_126.tif]

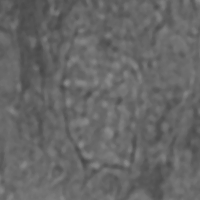

Supplement: Supplementary file 5 — Supplementary Information 5. [file 41598_2022_13394_MOESM5_ESM.zip › Supplementary Figure S4/Supplementary_Figure_S4_127.tif]

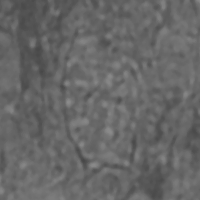

Supplement: Supplementary file 5 — Supplementary Information 5. [file 41598_2022_13394_MOESM5_ESM.zip › Supplementary Figure S4/Supplementary_Figure_S4_128.tif]

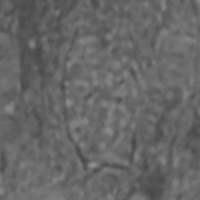

Supplement: Supplementary file 5 — Supplementary Information 5. [file 41598_2022_13394_MOESM5_ESM.zip › Supplementary Figure S4/Supplementary_Figure_S4_129.tif]

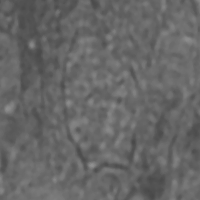

Supplement: Supplementary file 5 — Supplementary Information 5. [file 41598_2022_13394_MOESM5_ESM.zip › Supplementary Figure S4/Supplementary_Figure_S4_130.tif]

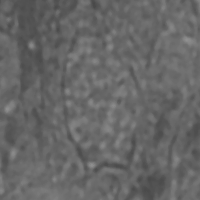

Supplement: Supplementary file 5 — Supplementary Information 5. [file 41598_2022_13394_MOESM5_ESM.zip › Supplementary Figure S4/Supplementary_Figure_S4_131.tif]

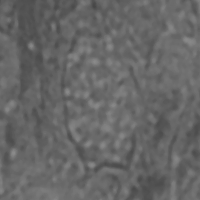

Supplement: Supplementary file 5 — Supplementary Information 5. [file 41598_2022_13394_MOESM5_ESM.zip › Supplementary Figure S4/Supplementary_Figure_S4_132.tif]

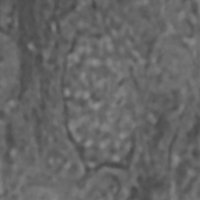

Supplement: Supplementary file 5 — Supplementary Information 5. [file 41598_2022_13394_MOESM5_ESM.zip › Supplementary Figure S4/Supplementary_Figure_S4_133.tif]

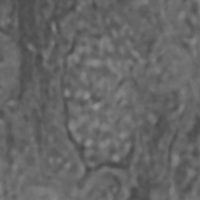

Supplement: Supplementary file 5 — Supplementary Information 5. [file 41598_2022_13394_MOESM5_ESM.zip › Supplementary Figure S4/Supplementary_Figure_S4_134.tif]

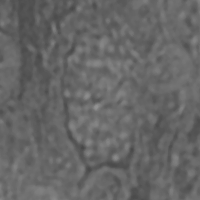

Supplement: Supplementary file 5 — Supplementary Information 5. [file 41598_2022_13394_MOESM5_ESM.zip › Supplementary Figure S4/Supplementary_Figure_S4_135.tif]

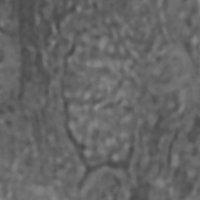

Supplement: Supplementary file 5 — Supplementary Information 5. [file 41598_2022_13394_MOESM5_ESM.zip › Supplementary Figure S4/Supplementary_Figure_S4_136.tif]

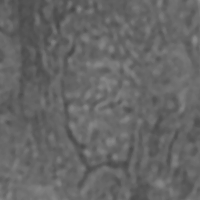

Supplement: Supplementary file 5 — Supplementary Information 5. [file 41598_2022_13394_MOESM5_ESM.zip › Supplementary Figure S4/Supplementary_Figure_S4_137.tif]

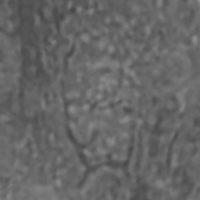

Supplement: Supplementary file 5 — Supplementary Information 5. [file 41598_2022_13394_MOESM5_ESM.zip › Supplementary Figure S4/Supplementary_Figure_S4_138.tif]

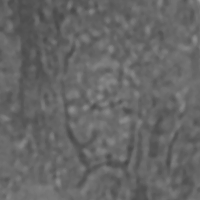

Supplement: Supplementary file 5 — Supplementary Information 5. [file 41598_2022_13394_MOESM5_ESM.zip › Supplementary Figure S4/Supplementary_Figure_S4_139.tif]

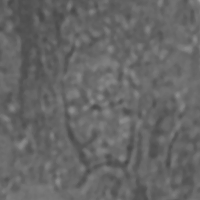

Supplement: Supplementary file 5 — Supplementary Information 5. [file 41598_2022_13394_MOESM5_ESM.zip › Supplementary Figure S4/Supplementary_Figure_S4_140.tif]

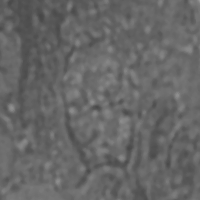

Supplement: Supplementary file 5 — Supplementary Information 5. [file 41598_2022_13394_MOESM5_ESM.zip › Supplementary Figure S4/Supplementary_Figure_S4_141.tif]

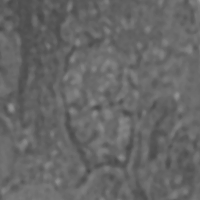

Supplement: Supplementary file 5 — Supplementary Information 5. [file 41598_2022_13394_MOESM5_ESM.zip › Supplementary Figure S4/Supplementary_Figure_S4_142.tif]

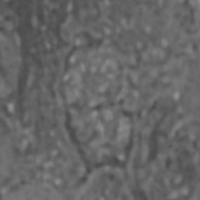

Supplement: Supplementary file 5 — Supplementary Information 5. [file 41598_2022_13394_MOESM5_ESM.zip › Supplementary Figure S4/Supplementary_Figure_S4_143.tif]

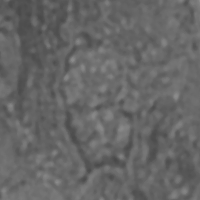

Supplement: Supplementary file 5 — Supplementary Information 5. [file 41598_2022_13394_MOESM5_ESM.zip › Supplementary Figure S4/Supplementary_Figure_S4_144.tif]

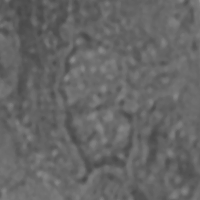

Supplement: Supplementary file 5 — Supplementary Information 5. [file 41598_2022_13394_MOESM5_ESM.zip › Supplementary Figure S4/Supplementary_Figure_S4_145.tif]

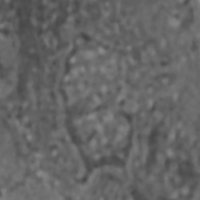

Supplement: Supplementary file 5 — Supplementary Information 5. [file 41598_2022_13394_MOESM5_ESM.zip › Supplementary Figure S4/Supplementary_Figure_S4_146.tif]

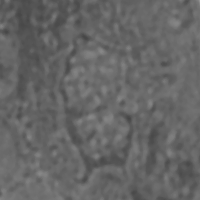

Supplement: Supplementary file 5 — Supplementary Information 5. [file 41598_2022_13394_MOESM5_ESM.zip › Supplementary Figure S4/Supplementary_Figure_S4_147.tif]

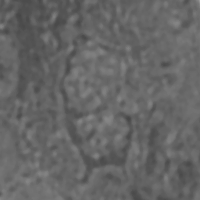

Supplement: Supplementary file 5 — Supplementary Information 5. [file 41598_2022_13394_MOESM5_ESM.zip › Supplementary Figure S4/Supplementary_Figure_S4_148.tif]

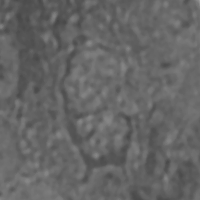

Supplement: Supplementary file 5 — Supplementary Information 5. [file 41598_2022_13394_MOESM5_ESM.zip › Supplementary Figure S4/Supplementary_Figure_S4_149.tif]

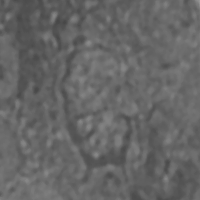

Supplement: Supplementary file 5 — Supplementary Information 5. [file 41598_2022_13394_MOESM5_ESM.zip › Supplementary Figure S4/Supplementary_Figure_S4_150.tif]

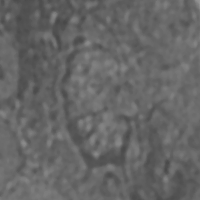

Supplement: Supplementary file 5 — Supplementary Information 5. [file 41598_2022_13394_MOESM5_ESM.zip › Supplementary Figure S4/Supplementary_Figure_S4_151.tif]

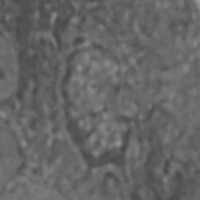

Supplement: Supplementary file 5 — Supplementary Information 5. [file 41598_2022_13394_MOESM5_ESM.zip › Supplementary Figure S4/Supplementary_Figure_S4_152.tif]

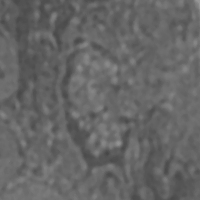

Supplement: Supplementary file 5 — Supplementary Information 5. [file 41598_2022_13394_MOESM5_ESM.zip › Supplementary Figure S4/Supplementary_Figure_S4_153.tif]

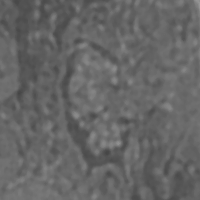

Supplement: Supplementary file 5 — Supplementary Information 5. [file 41598_2022_13394_MOESM5_ESM.zip › Supplementary Figure S4/Supplementary_Figure_S4_154.tif]

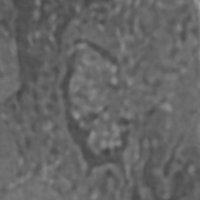

Supplement: Supplementary file 5 — Supplementary Information 5. [file 41598_2022_13394_MOESM5_ESM.zip › Supplementary Figure S4/Supplementary_Figure_S4_155.tif]

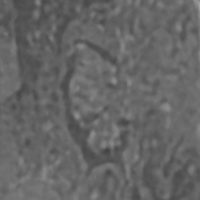

Supplement: Supplementary file 5 — Supplementary Information 5. [file 41598_2022_13394_MOESM5_ESM.zip › Supplementary Figure S4/Supplementary_Figure_S4_156.tif]

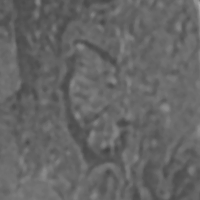

Supplement: Supplementary file 5 — Supplementary Information 5. [file 41598_2022_13394_MOESM5_ESM.zip › Supplementary Figure S4/Supplementary_Figure_S4_157.tif]

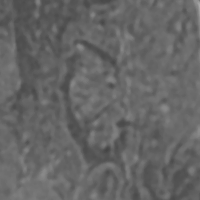

Supplement: Supplementary file 5 — Supplementary Information 5. [file 41598_2022_13394_MOESM5_ESM.zip › Supplementary Figure S4/Supplementary_Figure_S4_158.tif]

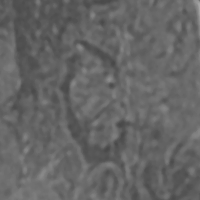

Supplement: Supplementary file 5 — Supplementary Information 5. [file 41598_2022_13394_MOESM5_ESM.zip › Supplementary Figure S4/Supplementary_Figure_S4_159.tif]

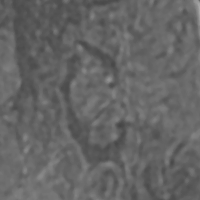

Supplement: Supplementary file 5 — Supplementary Information 5. [file 41598_2022_13394_MOESM5_ESM.zip › Supplementary Figure S4/Supplementary_Figure_S4_160.tif]

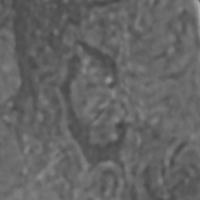

Supplement: Supplementary file 5 — Supplementary Information 5. [file 41598_2022_13394_MOESM5_ESM.zip › Supplementary Figure S4/Supplementary_Figure_S4_161.tif]

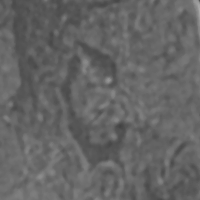

Supplement: Supplementary file 5 — Supplementary Information 5. [file 41598_2022_13394_MOESM5_ESM.zip › Supplementary Figure S4/Supplementary_Figure_S4_162.tif]

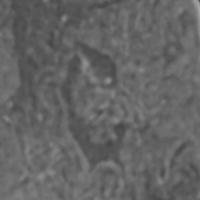

Supplement: Supplementary file 5 — Supplementary Information 5. [file 41598_2022_13394_MOESM5_ESM.zip › Supplementary Figure S4/Supplementary_Figure_S4_163.tif]

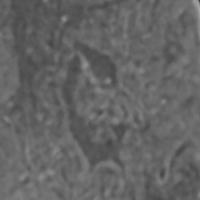

Supplement: Supplementary file 5 — Supplementary Information 5. [file 41598_2022_13394_MOESM5_ESM.zip › Supplementary Figure S4/Supplementary_Figure_S4_164.tif]

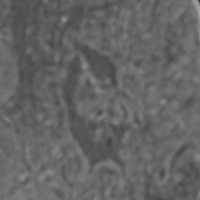

Supplement: Supplementary file 5 — Supplementary Information 5. [file 41598_2022_13394_MOESM5_ESM.zip › Supplementary Figure S4/Supplementary_Figure_S4_165.tif]

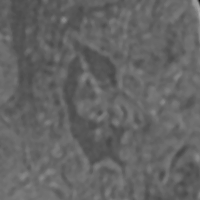

Supplement: Supplementary file 5 — Supplementary Information 5. [file 41598_2022_13394_MOESM5_ESM.zip › Supplementary Figure S4/Supplementary_Figure_S4_166.tif]

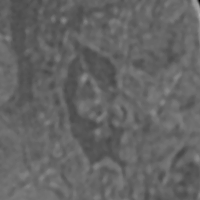

Supplement: Supplementary file 5 — Supplementary Information 5. [file 41598_2022_13394_MOESM5_ESM.zip › Supplementary Figure S4/Supplementary_Figure_S4_167.tif]

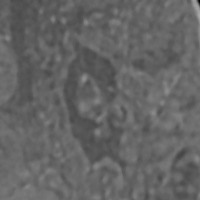

Supplement: Supplementary file 5 — Supplementary Information 5. [file 41598_2022_13394_MOESM5_ESM.zip › Supplementary Figure S4/Supplementary_Figure_S4_168.tif]

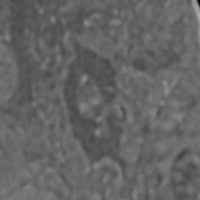

Supplement: Supplementary file 5 — Supplementary Information 5. [file 41598_2022_13394_MOESM5_ESM.zip › Supplementary Figure S4/Supplementary_Figure_S4_169.tif]

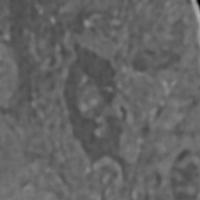

Supplement: Supplementary file 5 — Supplementary Information 5. [file 41598_2022_13394_MOESM5_ESM.zip › Supplementary Figure S4/Supplementary_Figure_S4_170.tif]

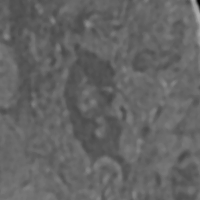

Supplement: Supplementary file 5 — Supplementary Information 5. [file 41598_2022_13394_MOESM5_ESM.zip › Supplementary Figure S4/Supplementary_Figure_S4_171.tif]

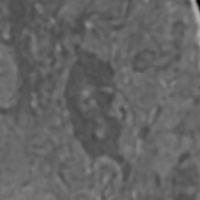

Supplement: Supplementary file 5 — Supplementary Information 5. [file 41598_2022_13394_MOESM5_ESM.zip › Supplementary Figure S4/Supplementary_Figure_S4_172.tif]

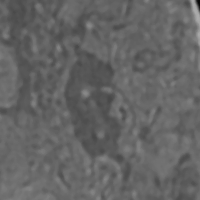

Supplement: Supplementary file 5 — Supplementary Information 5. [file 41598_2022_13394_MOESM5_ESM.zip › Supplementary Figure S4/Supplementary_Figure_S4_173.tif]

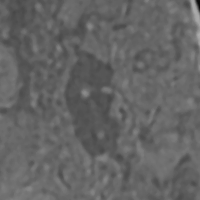

Supplement: Supplementary file 5 — Supplementary Information 5. [file 41598_2022_13394_MOESM5_ESM.zip › Supplementary Figure S4/Supplementary_Figure_S4_174.tif]

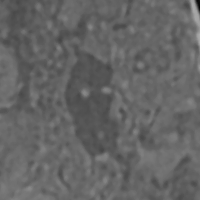

Supplement: Supplementary file 5 — Supplementary Information 5. [file 41598_2022_13394_MOESM5_ESM.zip › Supplementary Figure S4/Supplementary_Figure_S4_175.tif]

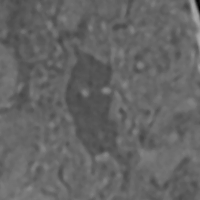

Supplement: Supplementary file 5 — Supplementary Information 5. [file 41598_2022_13394_MOESM5_ESM.zip › Supplementary Figure S4/Supplementary_Figure_S4_176.tif]

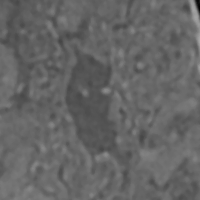

Supplement: Supplementary file 5 — Supplementary Information 5. [file 41598_2022_13394_MOESM5_ESM.zip › Supplementary Figure S4/Supplementary_Figure_S4_177.tif]

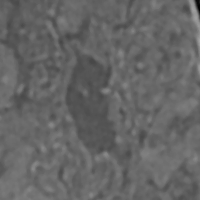

Supplement: Supplementary file 5 — Supplementary Information 5. [file 41598_2022_13394_MOESM5_ESM.zip › Supplementary Figure S4/Supplementary_Figure_S4_178.tif]

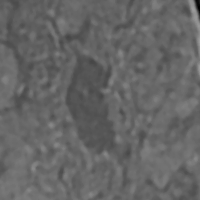

Supplement: Supplementary file 5 — Supplementary Information 5. [file 41598_2022_13394_MOESM5_ESM.zip › Supplementary Figure S4/Supplementary_Figure_S4_179.tif]

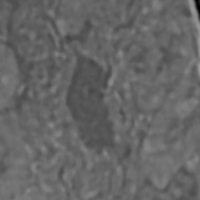

Supplement: Supplementary file 5 — Supplementary Information 5. [file 41598_2022_13394_MOESM5_ESM.zip › Supplementary Figure S4/Supplementary_Figure_S4_180.tif]

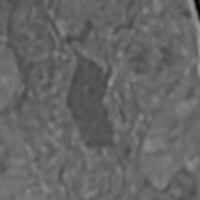

Supplement: Supplementary file 5 — Supplementary Information 5. [file 41598_2022_13394_MOESM5_ESM.zip › Supplementary Figure S4/Supplementary_Figure_S4_181.tif]

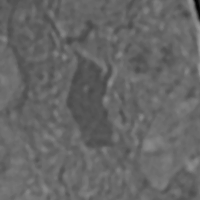

Supplement: Supplementary file 5 — Supplementary Information 5. [file 41598_2022_13394_MOESM5_ESM.zip › Supplementary Figure S4/Supplementary_Figure_S4_182.tif]

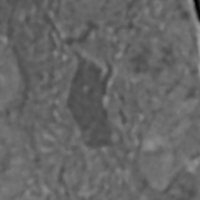

Supplement: Supplementary file 5 — Supplementary Information 5. [file 41598_2022_13394_MOESM5_ESM.zip › Supplementary Figure S4/Supplementary_Figure_S4_183.tif]

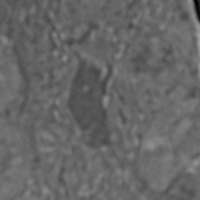

Supplement: Supplementary file 5 — Supplementary Information 5. [file 41598_2022_13394_MOESM5_ESM.zip › Supplementary Figure S4/Supplementary_Figure_S4_184.tif]

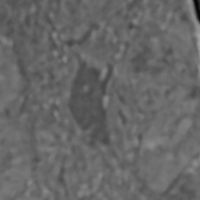

Supplement: Supplementary file 5 — Supplementary Information 5. [file 41598_2022_13394_MOESM5_ESM.zip › Supplementary Figure S4/Supplementary_Figure_S4_185.tif]

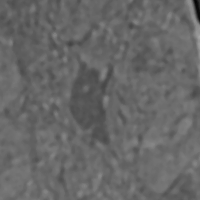

Supplement: Supplementary file 5 — Supplementary Information 5. [file 41598_2022_13394_MOESM5_ESM.zip › Supplementary Figure S4/Supplementary_Figure_S4_186.tif]

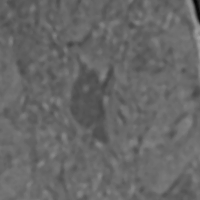

Supplement: Supplementary file 5 — Supplementary Information 5. [file 41598_2022_13394_MOESM5_ESM.zip › Supplementary Figure S4/Supplementary_Figure_S4_187.tif]

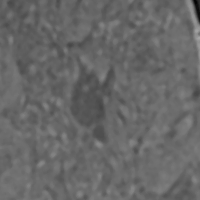

Supplement: Supplementary file 5 — Supplementary Information 5. [file 41598_2022_13394_MOESM5_ESM.zip › Supplementary Figure S4/Supplementary_Figure_S4_188.tif]

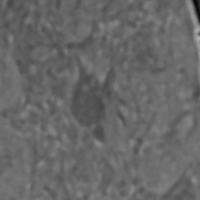

Supplement: Supplementary file 5 — Supplementary Information 5. [file 41598_2022_13394_MOESM5_ESM.zip › Supplementary Figure S4/Supplementary_Figure_S4_189.tif]

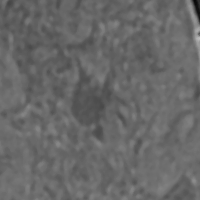

Supplement: Supplementary file 5 — Supplementary Information 5. [file 41598_2022_13394_MOESM5_ESM.zip › Supplementary Figure S4/Supplementary_Figure_S4_190.tif]

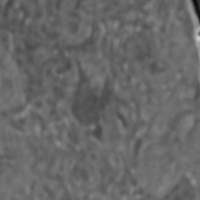

Supplement: Supplementary file 5 — Supplementary Information 5. [file 41598_2022_13394_MOESM5_ESM.zip › Supplementary Figure S4/Supplementary_Figure_S4_191.tif]

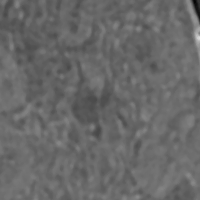

Supplement: Supplementary file 5 — Supplementary Information 5. [file 41598_2022_13394_MOESM5_ESM.zip › Supplementary Figure S4/Supplementary_Figure_S4_192.tif]

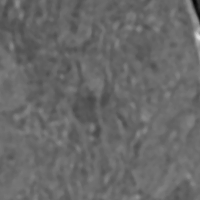

Supplement: Supplementary file 5 — Supplementary Information 5. [file 41598_2022_13394_MOESM5_ESM.zip › Supplementary Figure S4/Supplementary_Figure_S4_193.tif]

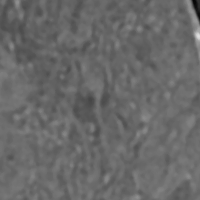

Supplement: Supplementary file 5 — Supplementary Information 5. [file 41598_2022_13394_MOESM5_ESM.zip › Supplementary Figure S4/Supplementary_Figure_S4_194.tif]

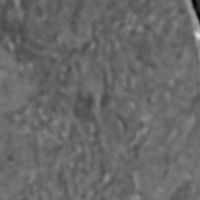

Supplement: Supplementary file 5 — Supplementary Information 5. [file 41598_2022_13394_MOESM5_ESM.zip › Supplementary Figure S4/Supplementary_Figure_S4_195.tif]

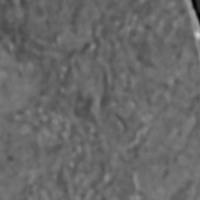

Supplement: Supplementary file 5 — Supplementary Information 5. [file 41598_2022_13394_MOESM5_ESM.zip › Supplementary Figure S4/Supplementary_Figure_S4_196.tif]

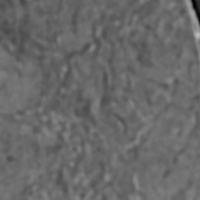

Supplement: Supplementary file 5 — Supplementary Information 5. [file 41598_2022_13394_MOESM5_ESM.zip › Supplementary Figure S4/Supplementary_Figure_S4_197.tif]

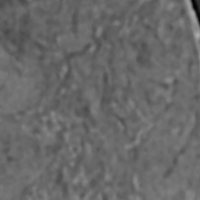

Supplement: Supplementary file 5 — Supplementary Information 5. [file 41598_2022_13394_MOESM5_ESM.zip › Supplementary Figure S4/Supplementary_Figure_S4_198.tif]
