# Supplementary figures and images for: Nondestructive cellular-level 3D observation of mouse kidney using laboratory-based X-ray microscopy with paraffin-mediated contrast enhancement (part 9 of 9)
Source: Sci Rep. 2022 Jun 8;12:9436. doi: 10.1038/s41598-022-13394-9 (PMC9177607; doi:10.1038/s41598-022-13394-9)

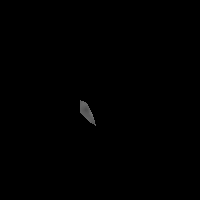

Supplement: Supplementary file 6 — Supplementary Information 6. [file 41598_2022_13394_MOESM6_ESM.zip › Supplementary Figure S5/Supplementary_Figure_S5_199.tif]

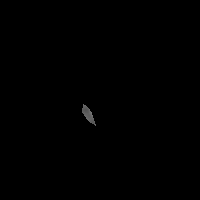

Supplement: Supplementary file 6 — Supplementary Information 6. [file 41598_2022_13394_MOESM6_ESM.zip › Supplementary Figure S5/Supplementary_Figure_S5_200.tif]
