# Supplementary figures and images for: Nondestructive cellular-level 3D observation of mouse kidney using laboratory-based X-ray microscopy with paraffin-mediated contrast enhancement (part 8 of 9)
Source: Sci Rep. 2022 Jun 8;12:9436. doi: 10.1038/s41598-022-13394-9 (PMC9177607; doi:10.1038/s41598-022-13394-9)

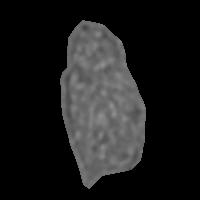

Supplement: Supplementary file 6 — Supplementary Information 6. [file 41598_2022_13394_MOESM6_ESM.zip › Supplementary Figure S5/Supplementary_Figure_S5_099.tif]

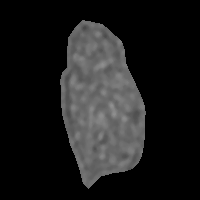

Supplement: Supplementary file 6 — Supplementary Information 6. [file 41598_2022_13394_MOESM6_ESM.zip › Supplementary Figure S5/Supplementary_Figure_S5_100.tif]

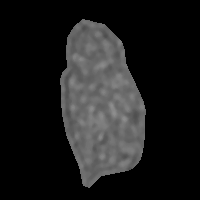

Supplement: Supplementary file 6 — Supplementary Information 6. [file 41598_2022_13394_MOESM6_ESM.zip › Supplementary Figure S5/Supplementary_Figure_S5_101.tif]

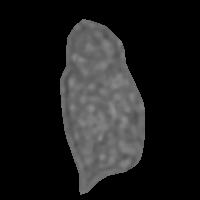

Supplement: Supplementary file 6 — Supplementary Information 6. [file 41598_2022_13394_MOESM6_ESM.zip › Supplementary Figure S5/Supplementary_Figure_S5_102.tif]

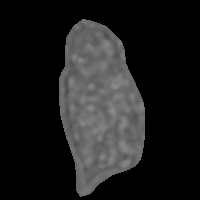

Supplement: Supplementary file 6 — Supplementary Information 6. [file 41598_2022_13394_MOESM6_ESM.zip › Supplementary Figure S5/Supplementary_Figure_S5_103.tif]

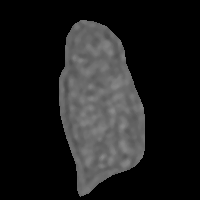

Supplement: Supplementary file 6 — Supplementary Information 6. [file 41598_2022_13394_MOESM6_ESM.zip › Supplementary Figure S5/Supplementary_Figure_S5_104.tif]

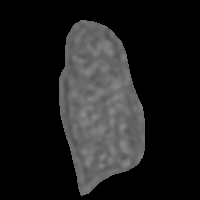

Supplement: Supplementary file 6 — Supplementary Information 6. [file 41598_2022_13394_MOESM6_ESM.zip › Supplementary Figure S5/Supplementary_Figure_S5_105.tif]

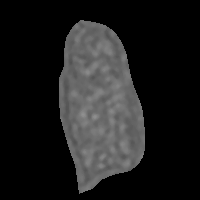

Supplement: Supplementary file 6 — Supplementary Information 6. [file 41598_2022_13394_MOESM6_ESM.zip › Supplementary Figure S5/Supplementary_Figure_S5_106.tif]

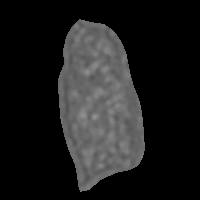

Supplement: Supplementary file 6 — Supplementary Information 6. [file 41598_2022_13394_MOESM6_ESM.zip › Supplementary Figure S5/Supplementary_Figure_S5_107.tif]

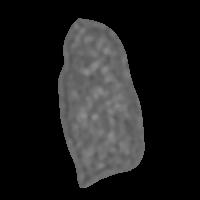

Supplement: Supplementary file 6 — Supplementary Information 6. [file 41598_2022_13394_MOESM6_ESM.zip › Supplementary Figure S5/Supplementary_Figure_S5_108.tif]

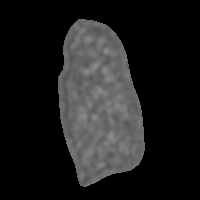

Supplement: Supplementary file 6 — Supplementary Information 6. [file 41598_2022_13394_MOESM6_ESM.zip › Supplementary Figure S5/Supplementary_Figure_S5_109.tif]

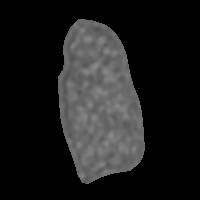

Supplement: Supplementary file 6 — Supplementary Information 6. [file 41598_2022_13394_MOESM6_ESM.zip › Supplementary Figure S5/Supplementary_Figure_S5_110.tif]

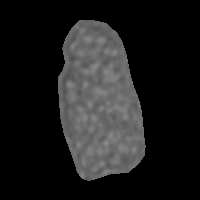

Supplement: Supplementary file 6 — Supplementary Information 6. [file 41598_2022_13394_MOESM6_ESM.zip › Supplementary Figure S5/Supplementary_Figure_S5_111.tif]

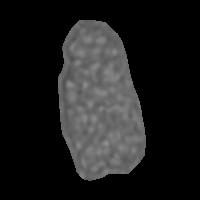

Supplement: Supplementary file 6 — Supplementary Information 6. [file 41598_2022_13394_MOESM6_ESM.zip › Supplementary Figure S5/Supplementary_Figure_S5_112.tif]

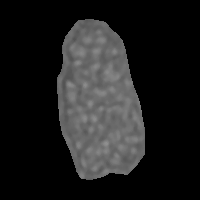

Supplement: Supplementary file 6 — Supplementary Information 6. [file 41598_2022_13394_MOESM6_ESM.zip › Supplementary Figure S5/Supplementary_Figure_S5_113.tif]

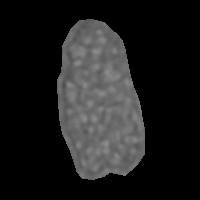

Supplement: Supplementary file 6 — Supplementary Information 6. [file 41598_2022_13394_MOESM6_ESM.zip › Supplementary Figure S5/Supplementary_Figure_S5_114.tif]

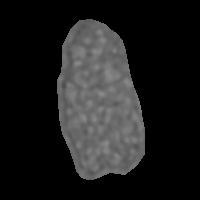

Supplement: Supplementary file 6 — Supplementary Information 6. [file 41598_2022_13394_MOESM6_ESM.zip › Supplementary Figure S5/Supplementary_Figure_S5_115.tif]

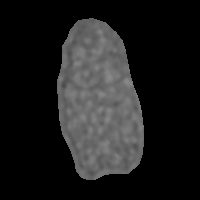

Supplement: Supplementary file 6 — Supplementary Information 6. [file 41598_2022_13394_MOESM6_ESM.zip › Supplementary Figure S5/Supplementary_Figure_S5_116.tif]

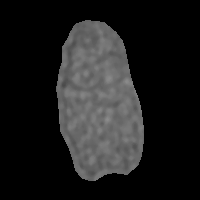

Supplement: Supplementary file 6 — Supplementary Information 6. [file 41598_2022_13394_MOESM6_ESM.zip › Supplementary Figure S5/Supplementary_Figure_S5_117.tif]

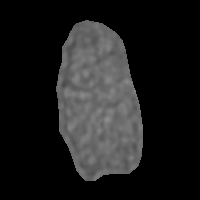

Supplement: Supplementary file 6 — Supplementary Information 6. [file 41598_2022_13394_MOESM6_ESM.zip › Supplementary Figure S5/Supplementary_Figure_S5_118.tif]

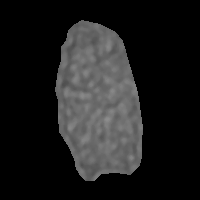

Supplement: Supplementary file 6 — Supplementary Information 6. [file 41598_2022_13394_MOESM6_ESM.zip › Supplementary Figure S5/Supplementary_Figure_S5_119.tif]

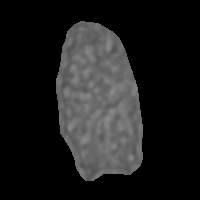

Supplement: Supplementary file 6 — Supplementary Information 6. [file 41598_2022_13394_MOESM6_ESM.zip › Supplementary Figure S5/Supplementary_Figure_S5_120.tif]

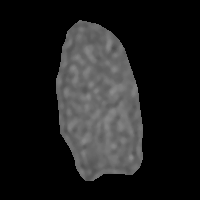

Supplement: Supplementary file 6 — Supplementary Information 6. [file 41598_2022_13394_MOESM6_ESM.zip › Supplementary Figure S5/Supplementary_Figure_S5_121.tif]

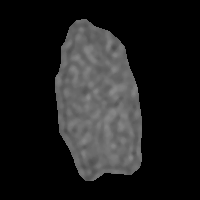

Supplement: Supplementary file 6 — Supplementary Information 6. [file 41598_2022_13394_MOESM6_ESM.zip › Supplementary Figure S5/Supplementary_Figure_S5_122.tif]

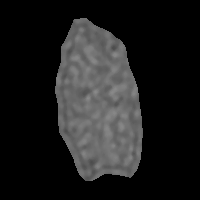

Supplement: Supplementary file 6 — Supplementary Information 6. [file 41598_2022_13394_MOESM6_ESM.zip › Supplementary Figure S5/Supplementary_Figure_S5_123.tif]

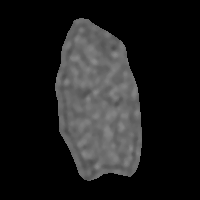

Supplement: Supplementary file 6 — Supplementary Information 6. [file 41598_2022_13394_MOESM6_ESM.zip › Supplementary Figure S5/Supplementary_Figure_S5_124.tif]

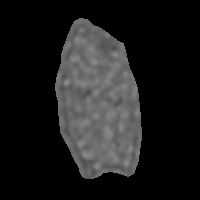

Supplement: Supplementary file 6 — Supplementary Information 6. [file 41598_2022_13394_MOESM6_ESM.zip › Supplementary Figure S5/Supplementary_Figure_S5_125.tif]

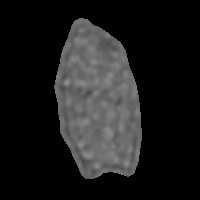

Supplement: Supplementary file 6 — Supplementary Information 6. [file 41598_2022_13394_MOESM6_ESM.zip › Supplementary Figure S5/Supplementary_Figure_S5_126.tif]

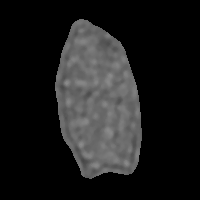

Supplement: Supplementary file 6 — Supplementary Information 6. [file 41598_2022_13394_MOESM6_ESM.zip › Supplementary Figure S5/Supplementary_Figure_S5_127.tif]

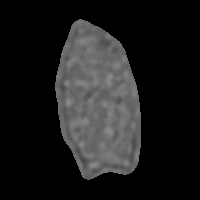

Supplement: Supplementary file 6 — Supplementary Information 6. [file 41598_2022_13394_MOESM6_ESM.zip › Supplementary Figure S5/Supplementary_Figure_S5_128.tif]

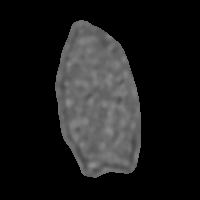

Supplement: Supplementary file 6 — Supplementary Information 6. [file 41598_2022_13394_MOESM6_ESM.zip › Supplementary Figure S5/Supplementary_Figure_S5_129.tif]

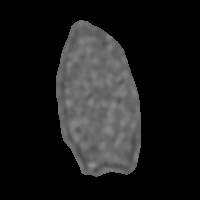

Supplement: Supplementary file 6 — Supplementary Information 6. [file 41598_2022_13394_MOESM6_ESM.zip › Supplementary Figure S5/Supplementary_Figure_S5_130.tif]

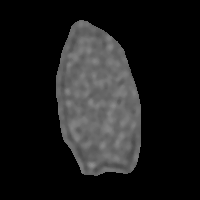

Supplement: Supplementary file 6 — Supplementary Information 6. [file 41598_2022_13394_MOESM6_ESM.zip › Supplementary Figure S5/Supplementary_Figure_S5_131.tif]

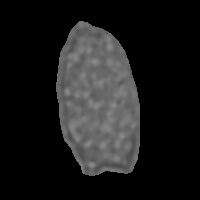

Supplement: Supplementary file 6 — Supplementary Information 6. [file 41598_2022_13394_MOESM6_ESM.zip › Supplementary Figure S5/Supplementary_Figure_S5_132.tif]

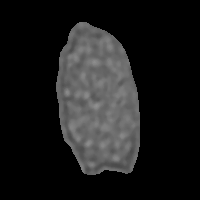

Supplement: Supplementary file 6 — Supplementary Information 6. [file 41598_2022_13394_MOESM6_ESM.zip › Supplementary Figure S5/Supplementary_Figure_S5_133.tif]

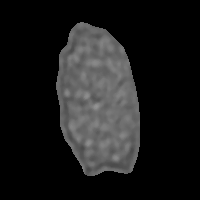

Supplement: Supplementary file 6 — Supplementary Information 6. [file 41598_2022_13394_MOESM6_ESM.zip › Supplementary Figure S5/Supplementary_Figure_S5_134.tif]

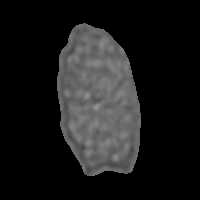

Supplement: Supplementary file 6 — Supplementary Information 6. [file 41598_2022_13394_MOESM6_ESM.zip › Supplementary Figure S5/Supplementary_Figure_S5_135.tif]

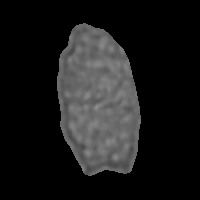

Supplement: Supplementary file 6 — Supplementary Information 6. [file 41598_2022_13394_MOESM6_ESM.zip › Supplementary Figure S5/Supplementary_Figure_S5_136.tif]

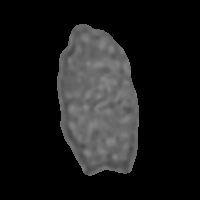

Supplement: Supplementary file 6 — Supplementary Information 6. [file 41598_2022_13394_MOESM6_ESM.zip › Supplementary Figure S5/Supplementary_Figure_S5_137.tif]

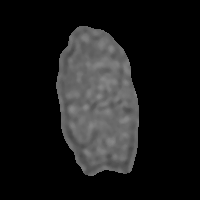

Supplement: Supplementary file 6 — Supplementary Information 6. [file 41598_2022_13394_MOESM6_ESM.zip › Supplementary Figure S5/Supplementary_Figure_S5_138.tif]

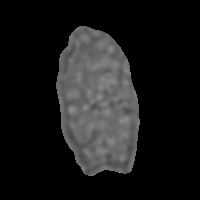

Supplement: Supplementary file 6 — Supplementary Information 6. [file 41598_2022_13394_MOESM6_ESM.zip › Supplementary Figure S5/Supplementary_Figure_S5_139.tif]

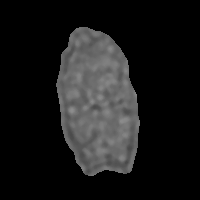

Supplement: Supplementary file 6 — Supplementary Information 6. [file 41598_2022_13394_MOESM6_ESM.zip › Supplementary Figure S5/Supplementary_Figure_S5_140.tif]

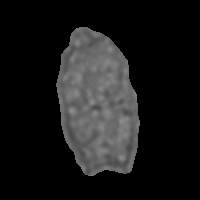

Supplement: Supplementary file 6 — Supplementary Information 6. [file 41598_2022_13394_MOESM6_ESM.zip › Supplementary Figure S5/Supplementary_Figure_S5_141.tif]

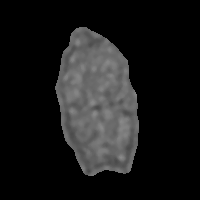

Supplement: Supplementary file 6 — Supplementary Information 6. [file 41598_2022_13394_MOESM6_ESM.zip › Supplementary Figure S5/Supplementary_Figure_S5_142.tif]

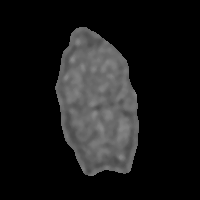

Supplement: Supplementary file 6 — Supplementary Information 6. [file 41598_2022_13394_MOESM6_ESM.zip › Supplementary Figure S5/Supplementary_Figure_S5_143.tif]

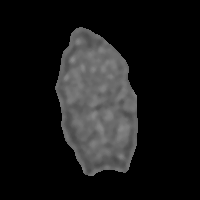

Supplement: Supplementary file 6 — Supplementary Information 6. [file 41598_2022_13394_MOESM6_ESM.zip › Supplementary Figure S5/Supplementary_Figure_S5_144.tif]

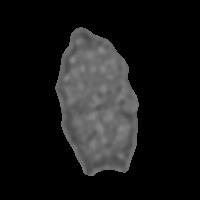

Supplement: Supplementary file 6 — Supplementary Information 6. [file 41598_2022_13394_MOESM6_ESM.zip › Supplementary Figure S5/Supplementary_Figure_S5_145.tif]

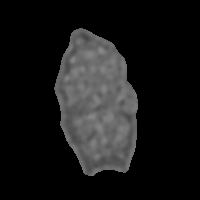

Supplement: Supplementary file 6 — Supplementary Information 6. [file 41598_2022_13394_MOESM6_ESM.zip › Supplementary Figure S5/Supplementary_Figure_S5_146.tif]

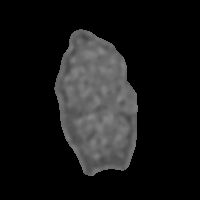

Supplement: Supplementary file 6 — Supplementary Information 6. [file 41598_2022_13394_MOESM6_ESM.zip › Supplementary Figure S5/Supplementary_Figure_S5_147.tif]

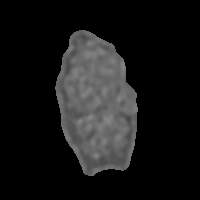

Supplement: Supplementary file 6 — Supplementary Information 6. [file 41598_2022_13394_MOESM6_ESM.zip › Supplementary Figure S5/Supplementary_Figure_S5_148.tif]

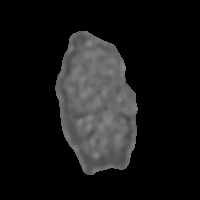

Supplement: Supplementary file 6 — Supplementary Information 6. [file 41598_2022_13394_MOESM6_ESM.zip › Supplementary Figure S5/Supplementary_Figure_S5_149.tif]

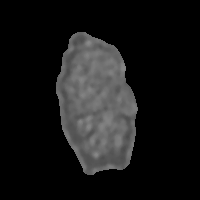

Supplement: Supplementary file 6 — Supplementary Information 6. [file 41598_2022_13394_MOESM6_ESM.zip › Supplementary Figure S5/Supplementary_Figure_S5_150.tif]

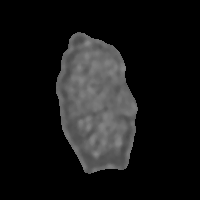

Supplement: Supplementary file 6 — Supplementary Information 6. [file 41598_2022_13394_MOESM6_ESM.zip › Supplementary Figure S5/Supplementary_Figure_S5_151.tif]

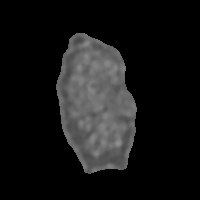

Supplement: Supplementary file 6 — Supplementary Information 6. [file 41598_2022_13394_MOESM6_ESM.zip › Supplementary Figure S5/Supplementary_Figure_S5_152.tif]

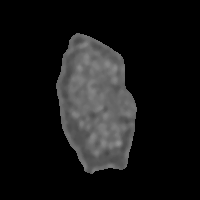

Supplement: Supplementary file 6 — Supplementary Information 6. [file 41598_2022_13394_MOESM6_ESM.zip › Supplementary Figure S5/Supplementary_Figure_S5_153.tif]

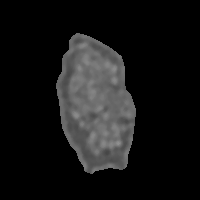

Supplement: Supplementary file 6 — Supplementary Information 6. [file 41598_2022_13394_MOESM6_ESM.zip › Supplementary Figure S5/Supplementary_Figure_S5_154.tif]

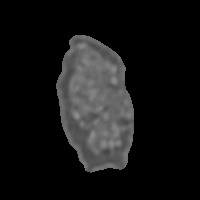

Supplement: Supplementary file 6 — Supplementary Information 6. [file 41598_2022_13394_MOESM6_ESM.zip › Supplementary Figure S5/Supplementary_Figure_S5_155.tif]

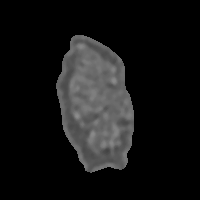

Supplement: Supplementary file 6 — Supplementary Information 6. [file 41598_2022_13394_MOESM6_ESM.zip › Supplementary Figure S5/Supplementary_Figure_S5_156.tif]

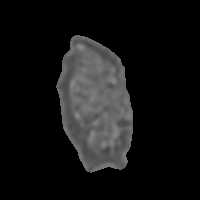

Supplement: Supplementary file 6 — Supplementary Information 6. [file 41598_2022_13394_MOESM6_ESM.zip › Supplementary Figure S5/Supplementary_Figure_S5_157.tif]

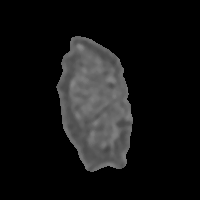

Supplement: Supplementary file 6 — Supplementary Information 6. [file 41598_2022_13394_MOESM6_ESM.zip › Supplementary Figure S5/Supplementary_Figure_S5_158.tif]

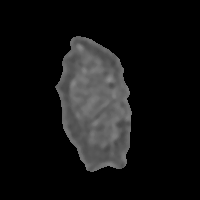

Supplement: Supplementary file 6 — Supplementary Information 6. [file 41598_2022_13394_MOESM6_ESM.zip › Supplementary Figure S5/Supplementary_Figure_S5_159.tif]

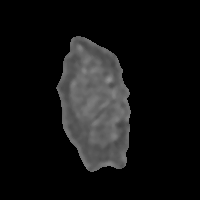

Supplement: Supplementary file 6 — Supplementary Information 6. [file 41598_2022_13394_MOESM6_ESM.zip › Supplementary Figure S5/Supplementary_Figure_S5_160.tif]

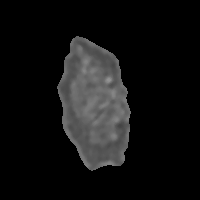

Supplement: Supplementary file 6 — Supplementary Information 6. [file 41598_2022_13394_MOESM6_ESM.zip › Supplementary Figure S5/Supplementary_Figure_S5_161.tif]

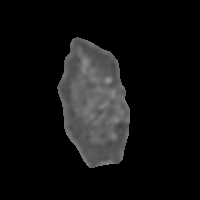

Supplement: Supplementary file 6 — Supplementary Information 6. [file 41598_2022_13394_MOESM6_ESM.zip › Supplementary Figure S5/Supplementary_Figure_S5_162.tif]

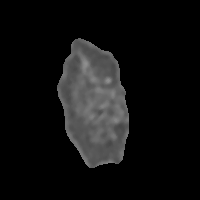

Supplement: Supplementary file 6 — Supplementary Information 6. [file 41598_2022_13394_MOESM6_ESM.zip › Supplementary Figure S5/Supplementary_Figure_S5_163.tif]

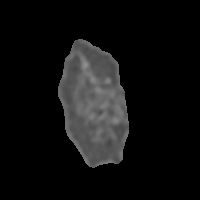

Supplement: Supplementary file 6 — Supplementary Information 6. [file 41598_2022_13394_MOESM6_ESM.zip › Supplementary Figure S5/Supplementary_Figure_S5_164.tif]

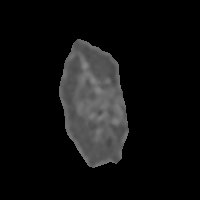

Supplement: Supplementary file 6 — Supplementary Information 6. [file 41598_2022_13394_MOESM6_ESM.zip › Supplementary Figure S5/Supplementary_Figure_S5_165.tif]

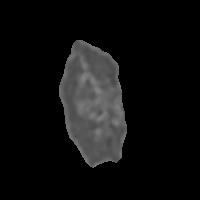

Supplement: Supplementary file 6 — Supplementary Information 6. [file 41598_2022_13394_MOESM6_ESM.zip › Supplementary Figure S5/Supplementary_Figure_S5_166.tif]

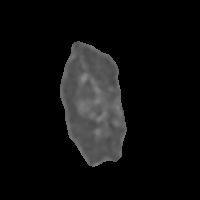

Supplement: Supplementary file 6 — Supplementary Information 6. [file 41598_2022_13394_MOESM6_ESM.zip › Supplementary Figure S5/Supplementary_Figure_S5_167.tif]

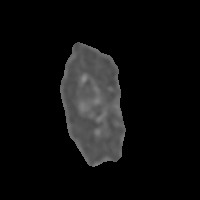

Supplement: Supplementary file 6 — Supplementary Information 6. [file 41598_2022_13394_MOESM6_ESM.zip › Supplementary Figure S5/Supplementary_Figure_S5_168.tif]

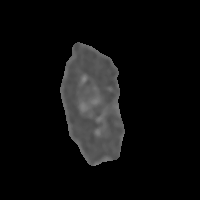

Supplement: Supplementary file 6 — Supplementary Information 6. [file 41598_2022_13394_MOESM6_ESM.zip › Supplementary Figure S5/Supplementary_Figure_S5_169.tif]

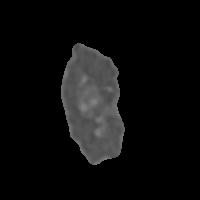

Supplement: Supplementary file 6 — Supplementary Information 6. [file 41598_2022_13394_MOESM6_ESM.zip › Supplementary Figure S5/Supplementary_Figure_S5_170.tif]

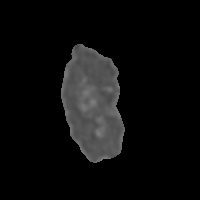

Supplement: Supplementary file 6 — Supplementary Information 6. [file 41598_2022_13394_MOESM6_ESM.zip › Supplementary Figure S5/Supplementary_Figure_S5_171.tif]

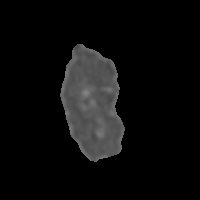

Supplement: Supplementary file 6 — Supplementary Information 6. [file 41598_2022_13394_MOESM6_ESM.zip › Supplementary Figure S5/Supplementary_Figure_S5_172.tif]

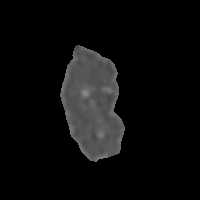

Supplement: Supplementary file 6 — Supplementary Information 6. [file 41598_2022_13394_MOESM6_ESM.zip › Supplementary Figure S5/Supplementary_Figure_S5_173.tif]

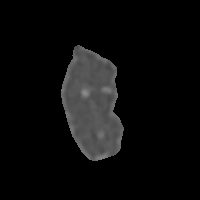

Supplement: Supplementary file 6 — Supplementary Information 6. [file 41598_2022_13394_MOESM6_ESM.zip › Supplementary Figure S5/Supplementary_Figure_S5_174.tif]

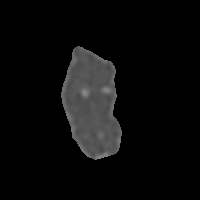

Supplement: Supplementary file 6 — Supplementary Information 6. [file 41598_2022_13394_MOESM6_ESM.zip › Supplementary Figure S5/Supplementary_Figure_S5_175.tif]

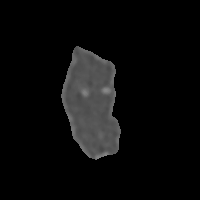

Supplement: Supplementary file 6 — Supplementary Information 6. [file 41598_2022_13394_MOESM6_ESM.zip › Supplementary Figure S5/Supplementary_Figure_S5_176.tif]

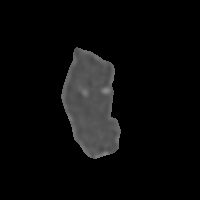

Supplement: Supplementary file 6 — Supplementary Information 6. [file 41598_2022_13394_MOESM6_ESM.zip › Supplementary Figure S5/Supplementary_Figure_S5_177.tif]

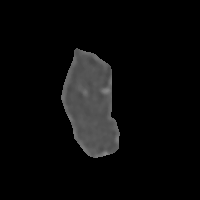

Supplement: Supplementary file 6 — Supplementary Information 6. [file 41598_2022_13394_MOESM6_ESM.zip › Supplementary Figure S5/Supplementary_Figure_S5_178.tif]

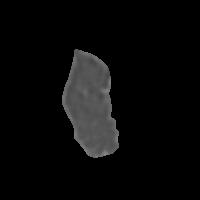

Supplement: Supplementary file 6 — Supplementary Information 6. [file 41598_2022_13394_MOESM6_ESM.zip › Supplementary Figure S5/Supplementary_Figure_S5_179.tif]

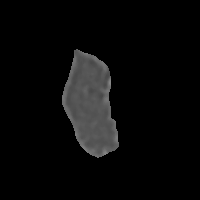

Supplement: Supplementary file 6 — Supplementary Information 6. [file 41598_2022_13394_MOESM6_ESM.zip › Supplementary Figure S5/Supplementary_Figure_S5_180.tif]

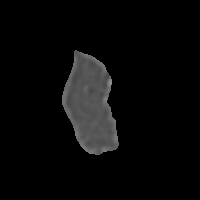

Supplement: Supplementary file 6 — Supplementary Information 6. [file 41598_2022_13394_MOESM6_ESM.zip › Supplementary Figure S5/Supplementary_Figure_S5_181.tif]

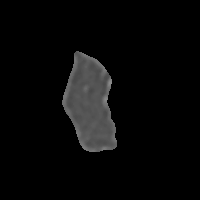

Supplement: Supplementary file 6 — Supplementary Information 6. [file 41598_2022_13394_MOESM6_ESM.zip › Supplementary Figure S5/Supplementary_Figure_S5_182.tif]

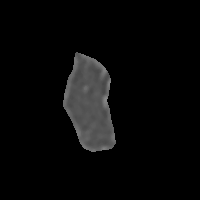

Supplement: Supplementary file 6 — Supplementary Information 6. [file 41598_2022_13394_MOESM6_ESM.zip › Supplementary Figure S5/Supplementary_Figure_S5_183.tif]

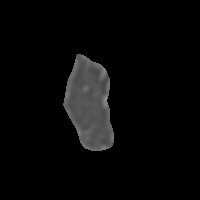

Supplement: Supplementary file 6 — Supplementary Information 6. [file 41598_2022_13394_MOESM6_ESM.zip › Supplementary Figure S5/Supplementary_Figure_S5_184.tif]

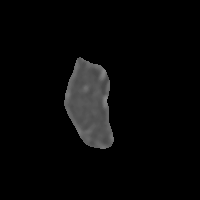

Supplement: Supplementary file 6 — Supplementary Information 6. [file 41598_2022_13394_MOESM6_ESM.zip › Supplementary Figure S5/Supplementary_Figure_S5_185.tif]

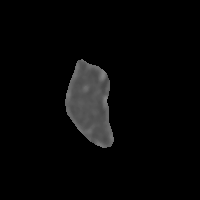

Supplement: Supplementary file 6 — Supplementary Information 6. [file 41598_2022_13394_MOESM6_ESM.zip › Supplementary Figure S5/Supplementary_Figure_S5_186.tif]

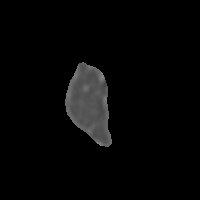

Supplement: Supplementary file 6 — Supplementary Information 6. [file 41598_2022_13394_MOESM6_ESM.zip › Supplementary Figure S5/Supplementary_Figure_S5_187.tif]

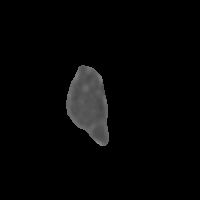

Supplement: Supplementary file 6 — Supplementary Information 6. [file 41598_2022_13394_MOESM6_ESM.zip › Supplementary Figure S5/Supplementary_Figure_S5_188.tif]

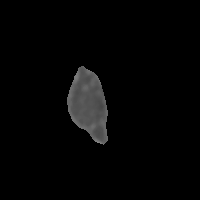

Supplement: Supplementary file 6 — Supplementary Information 6. [file 41598_2022_13394_MOESM6_ESM.zip › Supplementary Figure S5/Supplementary_Figure_S5_189.tif]

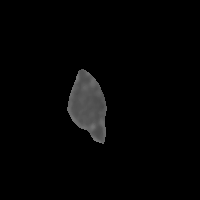

Supplement: Supplementary file 6 — Supplementary Information 6. [file 41598_2022_13394_MOESM6_ESM.zip › Supplementary Figure S5/Supplementary_Figure_S5_190.tif]

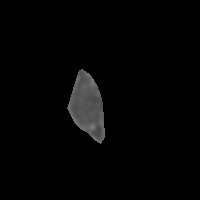

Supplement: Supplementary file 6 — Supplementary Information 6. [file 41598_2022_13394_MOESM6_ESM.zip › Supplementary Figure S5/Supplementary_Figure_S5_191.tif]

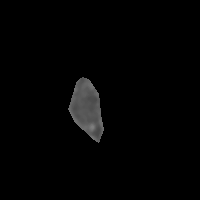

Supplement: Supplementary file 6 — Supplementary Information 6. [file 41598_2022_13394_MOESM6_ESM.zip › Supplementary Figure S5/Supplementary_Figure_S5_192.tif]

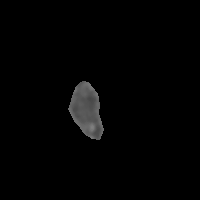

Supplement: Supplementary file 6 — Supplementary Information 6. [file 41598_2022_13394_MOESM6_ESM.zip › Supplementary Figure S5/Supplementary_Figure_S5_193.tif]

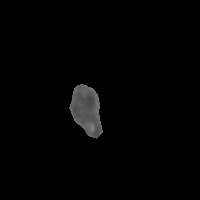

Supplement: Supplementary file 6 — Supplementary Information 6. [file 41598_2022_13394_MOESM6_ESM.zip › Supplementary Figure S5/Supplementary_Figure_S5_194.tif]

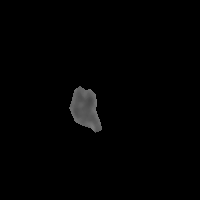

Supplement: Supplementary file 6 — Supplementary Information 6. [file 41598_2022_13394_MOESM6_ESM.zip › Supplementary Figure S5/Supplementary_Figure_S5_195.tif]

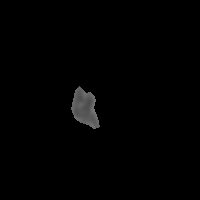

Supplement: Supplementary file 6 — Supplementary Information 6. [file 41598_2022_13394_MOESM6_ESM.zip › Supplementary Figure S5/Supplementary_Figure_S5_196.tif]

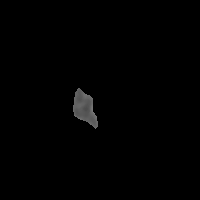

Supplement: Supplementary file 6 — Supplementary Information 6. [file 41598_2022_13394_MOESM6_ESM.zip › Supplementary Figure S5/Supplementary_Figure_S5_197.tif]

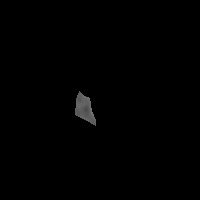

Supplement: Supplementary file 6 — Supplementary Information 6. [file 41598_2022_13394_MOESM6_ESM.zip › Supplementary Figure S5/Supplementary_Figure_S5_198.tif]
